# An Exploration of Heat Tolerance in Mice Utilizing mRNA and microRNA Expression Analysis

**DOI:** 10.1371/journal.pone.0072258

**Published:** 2013-08-15

**Authors:** Aminul Islam, Patricia A. Deuster, Joseph M. Devaney, Svetlana Ghimbovschi, Yifan Chen

**Affiliations:** 1 Department of Military and Emergency Medicine, Uniformed Services University of the Health Sciences, Bethesda, Maryland, United States of America; 2 Children’s National Medical Center, Department of Integrative Systems Biology, Washington DC, United States of America; Kyushu Institute of Technology, Japan

## Abstract

**Background:**

Individuals who rapidly develop hyperthermia during heat exposure (heat-intolerant) are vulnerable to heat associated illness and injury. We recently reported that heat intolerant mice exhibit complex alterations in stress proteins in response to heat exposure. In the present study, we further explored the role of genes and molecular networks associated with heat tolerance in mice.

**Methodology:**

Heat-induced physiological and biochemical changes were assessed to determine heat tolerance levels in mice. We performed RNA and microRNA expression profiling on mouse gastrocnemius muscle tissue samples to determine novel biological pathways associated with heat tolerance.

**Principal Findings:**

Mice (n = 18) were assigned to heat-tolerant (TOL) and heat-intolerant (INT) groups based on peak core temperatures during heat exposures. This was followed by biochemical assessments (Hsp40, Hsp72, Hsp90 and Hsf1 protein levels). Microarray analysis identified a total of 3,081 mRNA transcripts that were significantly misregulated in INT compared to TOL mice (p<0.05). Among them, Hspa1a, Dnajb1 and Hspb7 were differentially expressed by more than two-fold under these conditions. Furthermore, we identified 61 distinct microRNA (miRNA) sequences significantly associated with TOL compared to INT mice; eight miRNAs corresponded to target sites in seven genes identified as being associated with heat tolerance pathways (Hspa1a, Dnajb1, Dnajb4, Dnajb6, Hspa2, Hspb3 and Hspb7).

**Conclusions:**

The combination of mRNA and miRNA data from the skeletal muscle of adult mice following heat stress provides new insights into the pathophysiology of thermoregulatory disturbances of heat intolerance.

## Introduction

Excessive heat or physical exertion in hot climates can bring about heat exhaustion or heatstroke in individuals. This is becoming more apparent in recent years possibly due to a combination of factors including climate change and an ageing population [1]. It is evident that certain individuals seem to be more vulnerable to excessive physiological strain, and greater build-up of metabolic heat, which leads to higher core body temperatures and eventual heat exhaustion or heatstroke [2]. This group of individuals can be referred to as heat intolerant, and as a consequence may suffer debilitating physical effects or life-threatening complications [3].

Skeletal muscle is the largest of the major body tissues compared to heart and brown adipose tissue, which contributes significantly to the thermogenic process via central nervous system (CNS) thermoregulatory network stimulation [4]. Repeated muscle contraction results in net heat production as a result of inefficient energy utilization from processes such as calcium ion sequestration, ATP production from fuel substrate oxidation and cross-bridge cycling [5]. At the cellular level heat shock proteins (Hsps) serve crucial roles in counteracting the undesired effects of heat stress or heatstroke. It is thought that through their molecular chaperone role and subsequent auto-immune responses, Hsps provide protection and maintain protein homeostasis within the cell [6], [7]. In addition, Hsp transcription factors, such as heat shock factor-1 (Hsf1) are also reported to be up-regulated during heat stress, which thereby implies the involvement of transcriptional regulation in the various pathways responsible for cellular maintenance and defense against heat stress [8]. Numerous studies have identified Hsp70 to be very important in the processes contributing to heat tolerance [9]–[11]. However, cell culture experiments have shown that Hsp70 is not the sole mediator of heat tolerance [12], [13]. As a result other pathways and proteins must also exist that are able to confer heat tolerance in the absence of Hsp70.

Currently the pathogenesis of heat stress or heatstroke is poorly understood. Moreover the molecular mechanisms contributing to the process of heat tolerance is even less clear. Therefore, it is important to fully characterize the mechanisms of heat sensitivity and ultimately develop strategies to both detect and possibly prevent heat intolerance. We hypothesized that the predisposition of heat intolerance is a multi-factorial episode involving dysregulation of both genetic and adaptive biochemical stress responses. The expression cascade of mRNAs and micro RNAs (miRNAs) specific to heat tolerance remain largely unknown in mammalian skeletal muscle. Understanding such pathways would also be important to begin understanding rhabdomyolysis, a severe condition characterized by skeletal muscle degeneration and muscle enzyme leakage that develops as a result of exercise-induced heat injuries and malignant hyperthermia [14], [15]. To provide some insight into the associated mechanisms of heat tolerance, we utilized our mouse model of heat intolerance [16] combined with whole genome expression and miRNA analyses. In this work, we showed that acute heat exposure induces more extensive stress responses in various tissues of INT mice than TOL mice in terms of Hsps and corticosterone. Our current study presents targeted pathways that could possibly be activated or suppressed pharmacologically to prevent the negative effects of heat exhaustion/stroke.

## Materials and Methods

### Animal Studies

The experiments were conducted using 18 adult male C57BL/6J mice (Jackson Laboratories, Bar Harbor, ME). The mice were 10–12 weeks old and weighed 22–26 g, when heat tests were performed. They were maintained in conventional animal facilities (∼21°C) with *ad libitum* food and water at the Uniformed Services University (USU) Laboratory Animal Medicine facility. The USU Institutional Animal Care and Use Committee approved all procedures performed on animals.

All experimental mice were surgically implanted with a temperature transponder (Model G2 E-Mitter, Mini Mitter Corp, Bend, OR), as previously reported [16]. At least two weeks were allowed for recovery. At the time of the experimental protocols, all mice were healthy as evidenced by body weight gains (≥presurgical levels), normal behavior and no sign of infection. Heat tests were conducted in an environmental chamber (Model 3950, Thermo Forma, Marietta, OH). Mice were placed in the chamber at ∼21°C (relative humidity: ∼22–30%) a day before experimentation. Heat exposures began the following morning after stable baseline data were obtained. Food and water were removed from cages before exposure. All heat tests and telemetry measurements made to identify TOL and INT mice were performed as described [16].

### Collection and Processing of Tissues

Collection of tissues was performed under anesthesia ∼18–22 hours following the heat test. Subsequently, liver, heart (left ventricle) and gastrocnemius muscle were removed, cleaned in ice-cold PBS, frozen immediately in liquid nitrogen and stored at −80°C. Tissue samples were homogenized and further processed before analyses. Briefly, frozen tissues were placed into polypropylene test tubes containing 1 ml of ice-cold PBS and homogenized (5–10 seconds) with a Tissue Tearor homogenizer (Bartlesville, OK). The Tissue Tearor was cleaned in a series of fresh PBS filled beakers before homogenizing each sample tissue. Homogenates were centrifuged at 14,000 RPM for 3 min. The supernatants were obtained and placed into new 1.5 ml Eppendorf tubes and stored at −80°C. The remaining pellets were evaporated to dryness and weighed for data correction.

### Enzyme-linked Immunosorbant Assay (ELISA)

Tissue homogenate supernatants were measured in duplicates using commercial ELISA kits sensitive to murine samples. The following ELISA kits were used for: Hsp72 (Stressgen, Ann Arbor, MI), Hsp40, Hsp90 (TSZ ELISA, Framingham, MA) and Hsf1 (Enzo Life Sciences, Plymouth, PA) as per the manufacturers’ instructions. The aspirating and washing cycles were completed by using an automatic microplate washer (Tecan Group Ltd, Switzerland). Samples were analyzed using the Magellan Data Analysis System (Tecan, Austria) and normalized to dry tissue weight (dw). Sensitivity of the assays were; 35, 100, 200 and 60 pg/ml respectively. Intra- and inter-assay coefficients of variation for ELISA concentrations were less than 5% per assay.

### Western Blot Analysis

Homogenized tissue samples (25 µl, equivalent to 50 µg protein) were subjected to denaturing and reducing gel electrophoresis for 45 minutes in BioRad Tris glycine/SDS buffer (25 mM Tris, 192 mM glycine and 0.1% SDS) on BioRad 4–15% Tris–HCl (10 well/50 µl) precast Mini-Protean TGX gel cassettes by using a BioRad Mini-Protean Tetra Cell module at 200 V. This was followed by electrophoretic blotting onto a BioRad nitrocellulose membrane (0.2 µm) by using a BioRad Trans-Blot Turbo transfer system (Hercules, CA). Hsp40, Hsp72, Hsp90, and Hsf1 proteins were detected using primary mouse anti -Hsp40, -Hsp72, -Hsp90 and -Hsf1 antibodies (Santa Cruz, CA) diluted 1∶200 respectively and a horseradish peroxidase-conjugated goat anti-mouse IgG secondary antibody (GE Life Science, NJ) diluted 1∶1000. Selected blots were re-probed using a β-tubulin monoclonal antibody diluted 1∶200 (Santa Cruz, CA) to assess gel well loading efficiency.

### Microarray and Data Analysis

Total RNA was extracted from gastrocnemius muscle tissue by using a polytron homogenizer (Brinkman, Westbury, NY). MicroRNA was extracted from total RNA with a miRVana isolation kit (Ambion, Austin, TX) according to manufacturer’s instructions; quality was assessed using Agilent 2100 Bioanalyzer (Santa Clara, CA).

For mRNA expression, cDNA synthesis and amplification were completed as described by the manufacturer (Illumina Inc., San Diego, CA). Messenger RNA microarrays were performed using Illumina Gene Expression BeadChip Arrays (MouseWG-6v2) technology. Arrays were scanned using the HiScanSQ system and decoded images analyzed by GenomeStudio gene expression module (Illumina Inc.). Genomics Suite 6.5 (Partek Inc., St. Louis, MO) was used for statistical analyses and data visualization, and this software automatically applies Robust Multi-array Analysis (RMA) normalization algorithm and performs *log2* transformation for the generated expression values. Additionally, the GenomeStudio report table was used in Hierarchical Clustering Explorer 3.0 (HCEv3) for probe-set filtering, power analysis and Chip-based unsupervised clustering [17].

For miRNA expression, microarrays were performed using Affymetrix GeneChip miRNA 2.0 Arrays kit as described by the manufacturer (Affymetrix, Santa Clara, CA). The arrays were washed and stained on the Affymetrix Fluidics station 450 and scanned with an Affymetrix gene chip scanner 3000 7 G and analysis performed with Affymetrix® miRNA QC tool 1.1.1.0 (Affymetrix, Santa Clara, CA) for data summarization, normalization and microarrays quality control. Expression values were analyzed using Affymetrix Expression Console Summarization probe-set algorithm for miRNA using RMA and Detection Above the Background method. The signal values were filtered based on absent/present calls. Only miRNAs with present calls >10% were accepted for further analyses.

Partek Genomics Suite 6.5 was used for the statistics and data visualization analyses for differentially expressed genes. Partek Integration Tool (Partek Inc., St. Louis, MO) for mRNA and miRNA integration was used to determine mRNA targets for miRNA seed sequences. For all microarray data one-way ANOVA and *t*-test were applied to verify significance of the comparative results with p≤0.05 being considered significant for further analyses. All original microarray data are deposited in the NCBI GEO database (accession number: GSE48271).

### Pathway and Network Analysis

To determine significant molecular pathways and networks we used Ingenuity Pathways Analysis (IPA) software tool (Ingenuity systems Inc.). IPA generates networks for differentially expressed genes that can be related to previously known associations between genes or proteins. Every resulting gene interaction has supporting literature findings available online. IPA computes a score for each network according to the fit of the user’s set of significant genes. The core analysis function was used to interpret mRNA and miRNA statistically significant microarray data from Partek in the context of biological processes, pathways, and networks. Moreover, mRNA and miRNA integration networks were created and analyzed.

### Quantitative RT-PCR

RNA was isolated from tissue using RNeasy Mini Kit and QIAshredder (Qiagen, Valencia, CA), and cDNA templates were prepared with the Maloney murine leukemia virus reverse transcriptase directed iScript One-Step RT-PCR system (BioRad, Hercules,CA). PCR primers for Hspa1a (5′-TGGTGCAGTCCGACATGAAG-3′ and 5′-GCTGAGAGTCGTTGAAGTAGGC-3′), Dnajb1 (5′-TTCGACCGCTATGGAGAGGAA-3′ and 5′-CACCGAAGAACTCAGCAAACA-3′), Dnajb4 (5′-AAAGAGGTCGCAGAAGCGTAT-3′ and 5′-TCTCCGTGGAAAGTGTACCTG-3′), Dnajb6 (5′-CCGAGGAAATAGAAGCCGAGG-3′ and 5′-ACCTAGTGACCCAAATGGAGT-3′), Hspa2 (5′-GCGTGGGGGTATTCCAACAT-3′ and 5′-TGAGACGCTCGGTGTCAGT-3′), Hspb3 (5′-GACCCCAGTGCGTTATCAGG-3′ and 5′-GGCTTTACTCAGGTCCTCGAT-3′) and Hspb7 (5′-GAGCATGTTTTCAGACGACTTTG-3′ and 5′-CCGAGGGTCTTGATGTTTCCTT-3′) were synthesized by the Genomics Core at the Biomedical Instrumentation Center (USU, MD) and utilized for quantitative RT-PCR. Relevant TaqMan miRNA Assay probes were obtained from Life Technologies and used per manufacturers’ instructions (Grand Island, NY). Real time RT-PCR was performed for 40 cycles using the Bio-Rad iCycler iQ real time PCR thermocycler and iScript SYBR green PCR supermix (Hercules, CA). Quantification of the RT-PCR products normalized to glyceraldehyde-3-phosphate dehydrogenase (GAPDH) expression was performed using iCycler iQ data analysis software and comparative CT method.

### Data Processing and Statistical Analysis

Non-microarray data are expressed as mean ± SEM. Data were analyzed by a paired Student’s t-test. The results were considered significant at p≤0.05.

## Results

### Temperature and Biochemical Profile of Heat Tolerance in Mice

**Figure 1:**
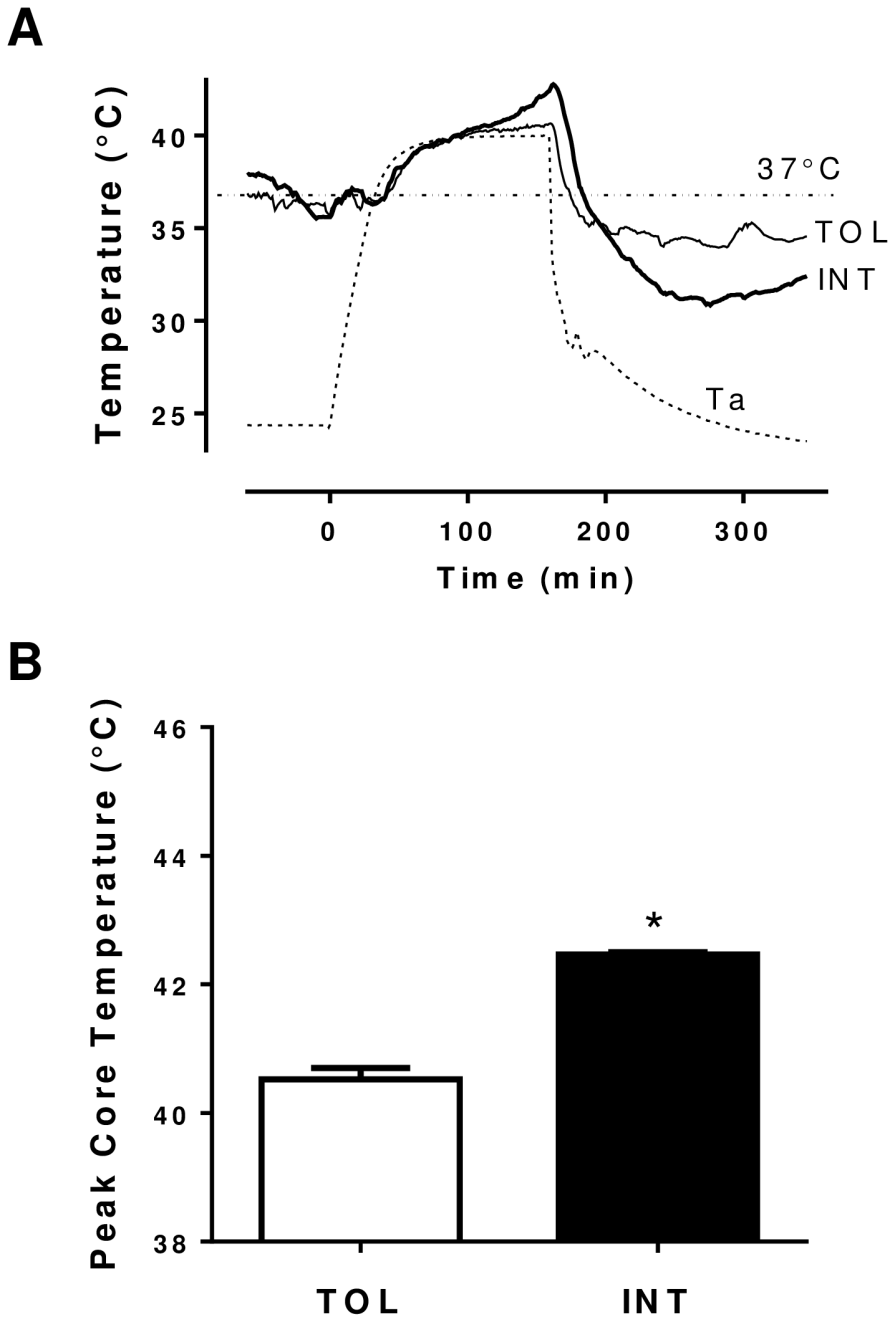
Temperature profiles associated with TOL and INT mice in response to heat exposure. (**A**) Representative tracings of animal core temperature before, during and after heat exposure. The signals were recorded simultaneously in real-time from a single experiment. The ambient temperature (Ta) was obtained inside an environmental chamber. (**B**) Average peak core temperature of TOL and INT mice during heat exposure experiments. *p<0.05 for differences (mean ± SEM, n = 6) between TOL and INT mice groups.

**Figure 2:**
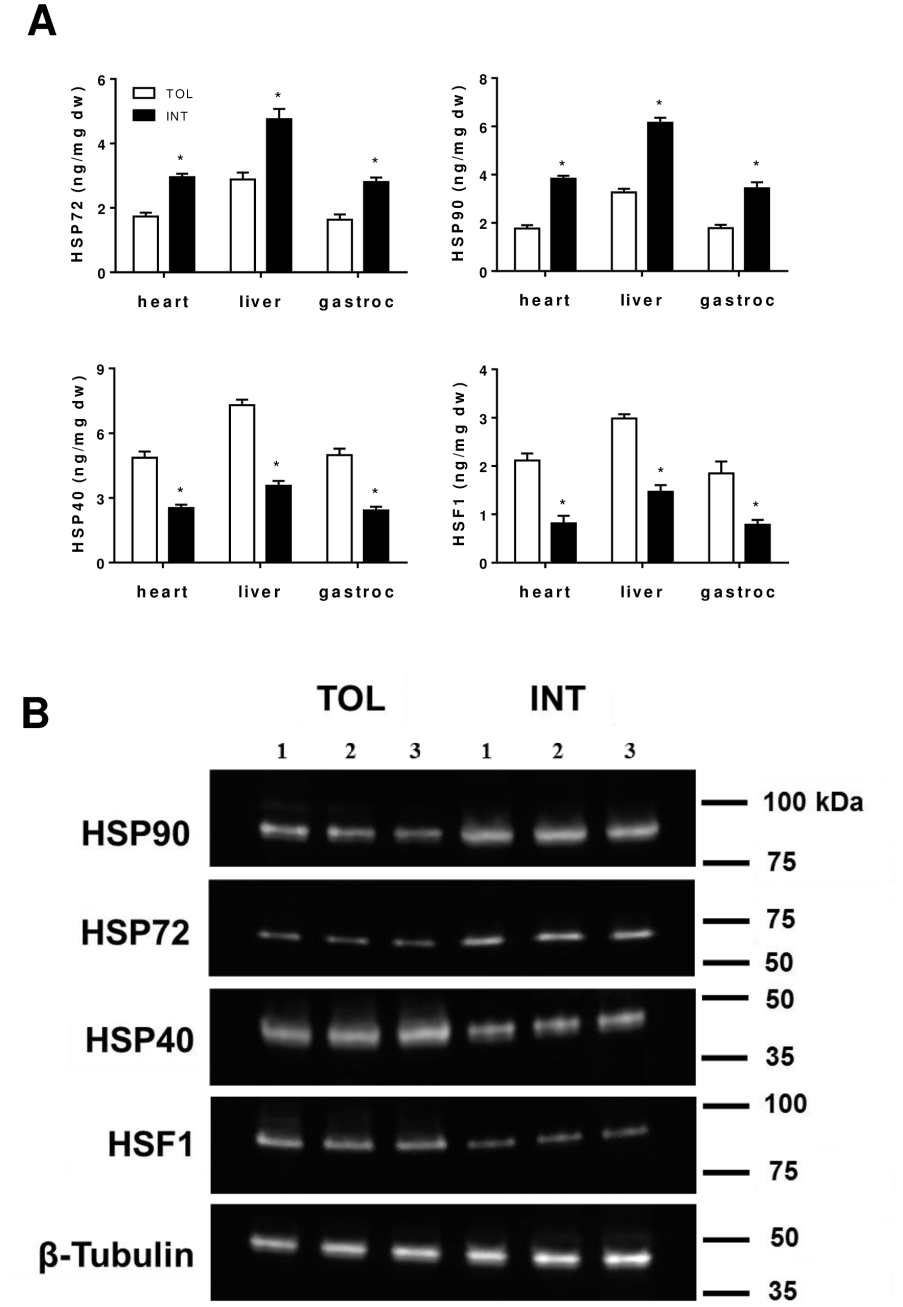
Hsp90, Hsp72, Hsp40 and Hsf1 protein levels in heart, liver and gastrocnemius muscle tissues of TOL and INT mice following heat stress experiment. (**A**) Analysis performed using ELISA. *p<0.05 for differences (mean ± SEM, n = 6) between TOL and INT mice groups. (**B**) Illustrative Western blot image comparing protein expression in gastrocnemius muscle tissue of TOL and INT mice (n = 3).

TOL and INT mice were identified as previously described using our heat exposure test [16]. From the 18 mice tested we identified six TOL and six INT mice, which exhibited specific group core body temperature profiles during the heat test as shown in Figure 1A and B. The remaining six mice were classified as moderately tolerant (MT) based on their thermal responses. Overall the TOL mice group had a slower hyperthermic rate and significantly (p<0.05) lower peak core temperature than the INT mice (Figure 1A and B). To further verify stress-related changes associated with heat tolerance, protein levels of Hsp90, Hsp72, Hsp40, and Hsf1 were selectively compared in heart, liver and gastrocnemius muscle of TOL and INT mice 18–22 hours following heat exposure. As determined by ELISA (Figure 2A) TOL mice had significantly lower Hsp90 and Hsp72 protein levels in heart, liver and gastrocnemius muscle respectively (Hsp90: 54, 47 and 48%, p<0.05; Hsp72: 41, 39 and 42%, p<0.05), relative to INT mice. In contrast, TOL mice had significantly higher Hsp40 and Hsf1 protein levels (Hsp40: 92, 104 and 106%, p<0.05; Hsf1: 159, 103 and 137%, p<0.05) in heart, liver and gastrocnemius muscle respectively relative to INT mice. These patterns of Hsp90, Hsp72, Hsp40 and Hsf1 protein expression were also confirmed by Western blot analysis in gastrocnemius muscle tissue samples (Figure 2B).

### Microarray Expression Analysis of Heat Tolerance in Mice

**Figure 3:**
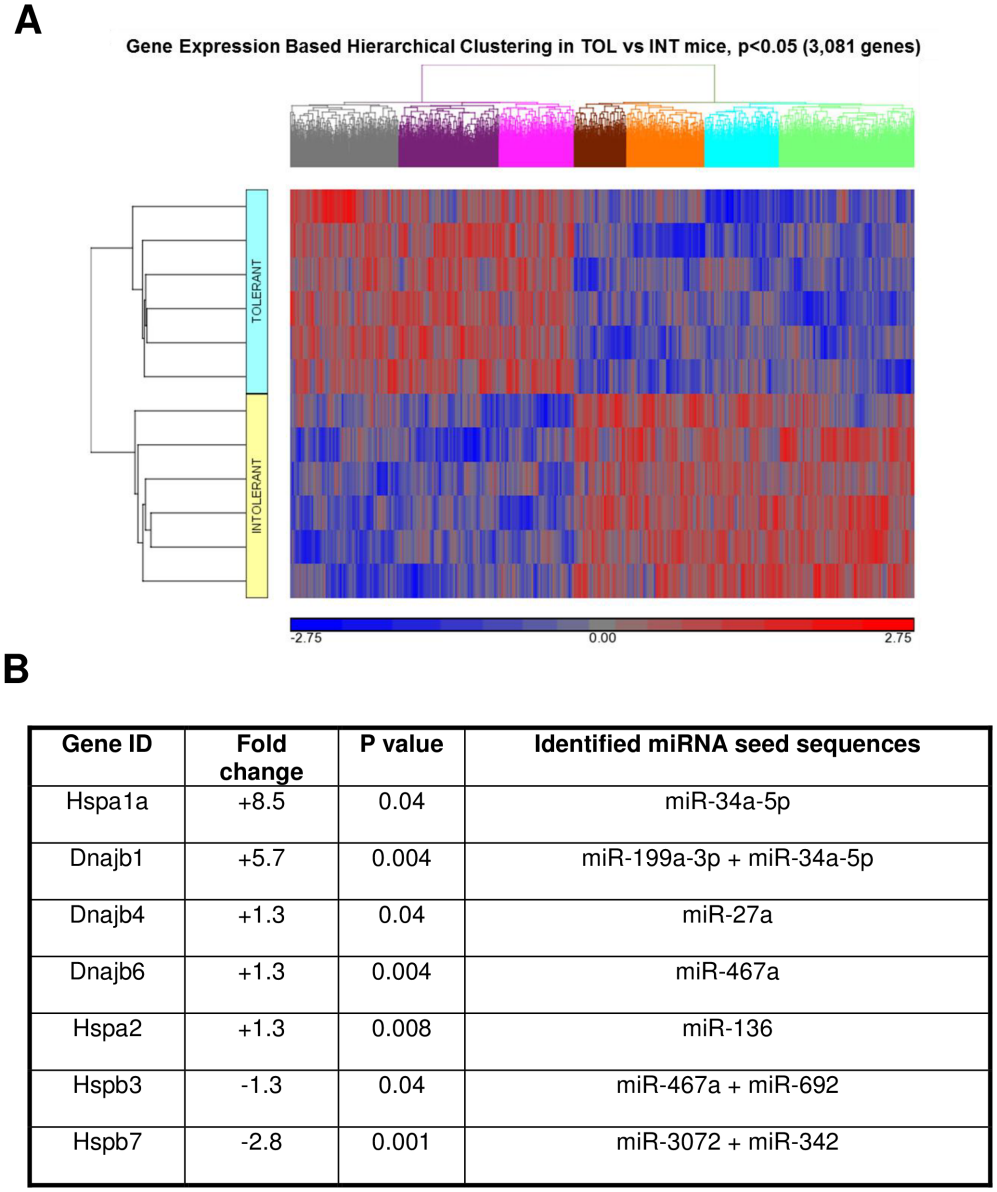
Gene expression profiles in gastrocnemius muscle tissue of TOL vs. INT mice. (**A**) Cluster analysis of microarray data from TOL (blue) and INT (yellow) mice groups. Here all genes that significantly changed were included in the analysis (p<0.05, n = 3,081). Results were generated using Partek Genomics Suite. The color code for the signal strength in the classification scheme is shown in the panel below. Induced genes are indicated by shades of red and repressed genes are indicated by shades of blue. (**B**) List of most highly changed genes with fold change and p-value significance relevant to heat tolerance pathways in TOL mice and their associated miRNA seed sequences as determined using microarray data analysis (p<0.05, n = 61).

**Figure 4:**
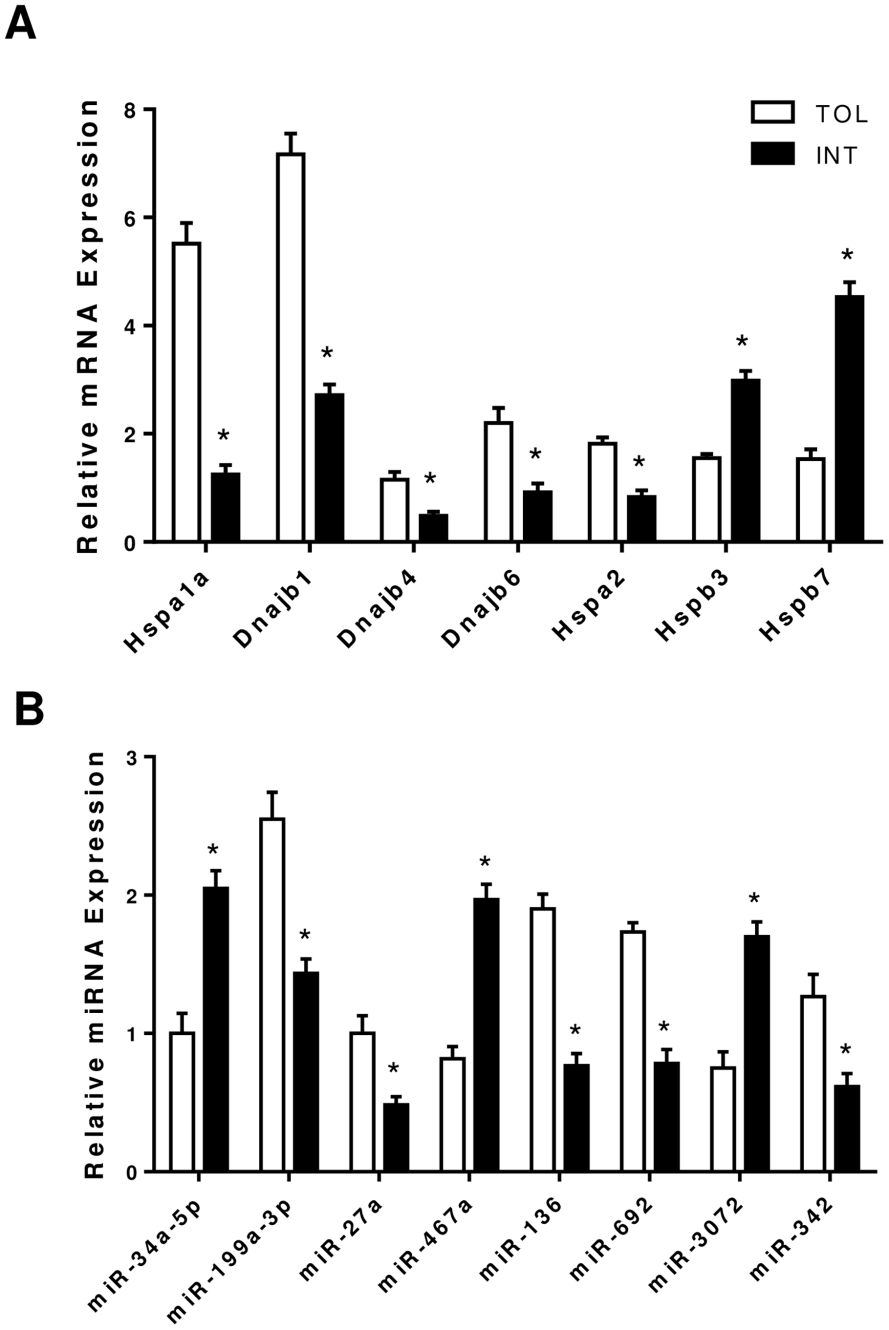
Validation of differential gene expression in gastrocnemius muscle tissue using quantitative RT-PCR. (**A**) mRNA expression of genes selected to be important in the heat tolerance pathways of TOL versus INT mice. *p<0.05 for differences (mean ± SEM, n = 6) between TOL and INT mice groups. (**B**) miRNA expression of target seed sequences determined to be important in the heat tolerance pathways of TOL mice. *p<0.05 for differences (mean ± SEM, n = 6) between TOL and INT mice groups.

A microarray analysis comparing gastrocnemius muscle tissue from TOL and INT mice groups (n = 6 per group) was conducted, and 3,081 genes were identified as being significantly (p<0.05) different in TOL mice compared to INT mice (Figure 3A and Table S1). The majority of the genes (91%) had less than a 1.5-fold difference and only about 1% of genes had more than a two-fold difference in expression levels. However seven genes with links to the thermal response were selected based on fold change and significance (Figure 3B). From these seven genes only the mRNA levels of three genes (Hspa1a, Dnajb1 and Hspb7) for the TOL group showed at least a two-fold difference compared to INT mice. All seven selected candidate genes identified using microarray data were validated using real-time quantitative RT-PCR (Figure 4A). In addition all three genes: Hspa1a, Dnajb1 and Hspb7 were validated to have mRNA levels greater than a two-fold difference between TOL and INT mice, as shown initially by microarray data. We also examined miRNA expression using microarrays. Sixty-one distinct miRNA seed sequences were identified as being significantly (p<0.05) different for TOL compared to INT mice (Table S2). All miRNA had less than 1.3-fold difference in expression levels. Combined mRNA and miRNA microarray data integration identified eight miRNA seed sequences corresponding significantly (p<0.05) to target sites of selected genes associated with the thermotolerance process (Figure 3B). In particular miRNA seed sequences miR-199a-3p and miR-34a-5p were associated with highest-ranking fold change genes Hspa1a and Dnajb1. These eight miRNA seed sequences were validated using real-time quantitative RT-PCR (Figure 4B).

### Analysis of Molecular Pathways in Heat Tolerant Mice

**Figure 5:**
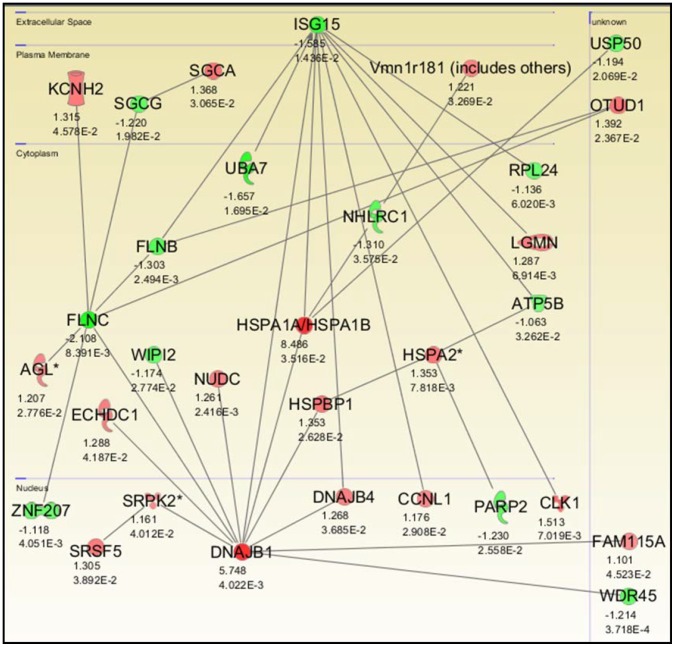
Significant gene networks in gastrocnemius muscle tissue associated with TOL mice. IPA-generated pathways important for heat tolerance with individual gene fold change and p-value significance. Both upregulated (red) and downregulated (green) genes were included in the analysis. Relationships are primarily due to co-expression, but may include phosphorylation/dephosphorylation, proteolysis, transcription, binding, inhibition, activation/deactivation, and biochemical modification. Only Hspa1a and Dnajb1 are differentially expressed by more than 2.5-fold within this network.

**Figure 6:**
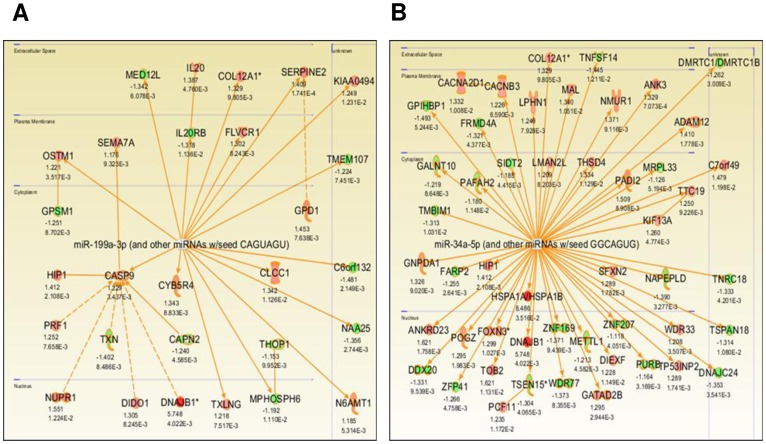
Gene networks in gastrocnemius muscle tissue associated with miRNA relevant to heat tolerance. Illustrated IPA-generated pathways associated with miRNA seed sequences (**A**) miR-199a-3p and (**B**) miR-34a-5p. Both upregulated (red) and downregulated (green) genes with fold change and p-value significance are shown. Only Dnajb1 (**A**) and both Hspa1a and Dnajb1 (**B**) are differentially expressed by more than 2-fold within their respective networks.

Figure 5 shows the genetic networks associated with TOL mice as defined by pathway analysis on the 3,081 genes found to be differentially expressed. The pathway analysis performed on our study data showed that the genes Hspa1a and Dnajb1 were significantly (p<0.05) up-regulated and to the greatest extent within the network by more than eight- and five- fold, respectively (Figure 5). In addition, pathway analysis was performed on the two miRNA seed sequences most relevant to TOL mice. Figure 6 shows the IPA-generated networks associated with miRNA seed sequences miR-199a-3p and miR-34a-5p. Dnajb1 was associated with seed sequence miR-199a-3p (Figure 6A) whereas both Dnajb1 and Hspa1a were associated with seed sequence miR-34a-5p (Figure 6B).

## Discussion

Inflammatory cytokines and their associated proteins together with Hsps serve a significant role in mediating the body’s response to heat stress and subsequent prognosis [18]. Skeletal muscle, which comprises about 40% of body weight, is very important for maintaining thermal homeostasis [4]. We recently reported that INT mice (categorized based on their overall thermal responses to heat stress) have significantly higher Hsp72 and Hsp90 proteins in skeletal muscle 18–22 hours following heat exposure, compared to TOL mice [16]. The rapid synthesis of heat shock proteins is a primary cellular defense against acute heat/inflammatory insults [6], [8]. Differential activation of muscle stress proteins between TOL and INT mice, which likely is one of the mechanisms for developing cellular inflammatory resistance and heat tolerance, remains poorly understood. In the present study, we compared gene mRNA and miRNA expression profiles in skeletal muscle of TOL mice and INT mice to identify possible molecular networks and pathways contributing to heat tolerance.

It is generally understood that under heat stress, Hsf1 mediates induction of Hsp gene expression [19], [20]. However, differential expression of other pathways and proteins that regulate Hsps might explain the dissimilar physiological and biochemical profiles of TOL and INT mice [21]. We demonstrate that heat intolerance in mice is associated with differential expression of Hsp72, Hsp40, Hsp90 and Hsf1 protein levels in major organs/tissues following heat exposure as well as higher peak core temperatures during heat exposure (see Figures 1 and 2) [16]. This suggests that under heat stress TOL mice may possibly have a novel gene-specific transcriptional regulation of Hsps that produces a higher thermogenic threshold compared to INT mice. Microarray data shown in Figure 3 identified 3,081 genes differentially expressed in TOL mice compared to INT mice (Table S1). However, only 1% of these genes differed more than two-fold in expression levels. To further refine this list of genes associated with heat tolerance we focused on genes known to interact biologically in thermoregulatory pathways, namely Hsps [13], [21]. This produced a list of three high ranking genes (Hspa1a, Dnajb1 and Hspb7), with two of these genes (Hspa1a and Dnajb1) having the highest fold-change signal of the entire pool, as determined by microarray analysis. Other genes from the Hsp category were also differentially expressed in TOL mice compared to INT mice, albeit to a lesser extent (<1.5-fold), and quantitative RT-PCR demonstrated up-regulation of Hspa1a and Dnajb1 and down-regulation of Hspb7 in TOL mice relative to INT mice. Our search to find networks describing functional relationships between gene products based on known interactions reported in the literature demonstrated that only Hspa1a and Dnajb1 are differentially expressed by more than 2.5-fold. The major contribution of these two genes to heat tolerance mechanisms have been previously reported, but have not been directly linked to processes involving skeletal muscle [8], [12], [13], [21]. Further studies will be needed to investigate these genes in more detail for their direct influence on mechanisms contributing to heat tolerance/thermoregulation.

The Hspa1a gene is the key component of the Hsp70 family, which is both expressed under normal conditions and substantively induced after heat stress following Hsf1 stimulated transcription of the gene [22], [23]. Immediate increases in the molecular chaperone Hsp70 are supposed to provide protection from the effects of heat stress [8], [9]. Our microarray data show that Hspa1a is the most preferentially up-regulated gene in skeletal muscle tissue of TOL mice. However INT mice have higher prolonged Hsp72 protein levels. This would suggest both the expression levels and altered processing of this gene might account for the observed differences between TOL and INT mice. Overall, abnormal regulation of these networks and genes could have important biological consequences in the skeletal muscle of heat sensitive individuals.

Genetic variations in the Hspa1a gene have been previously described to affect Hspa1a protein synthesis and produce susceptibility towards certain diseases such as hypertension [24], ischemic stroke [25] and coronary heart disease [26]. Thus genetic variations in the Hspa1a gene or its transcription factor Hsf1 (polymorphisms of the regulatory region and/or epigenetic differences) could account for the differences in heat tolerance between TOL and INT mice. Additional studies will be required to address this issue.

Members of the Hsp40 family of genes are specifically up-regulated in TOL compared to INT mice, and our data demonstrate that Dnajb1 has the second highest fold change value overall. Dnajb1 is a major Hsp40 member protein, which serves as a co-chaperone by interacting with and regulating Hsp70 function [27]. Dnajb1 recognizes substrate proteins and facilitates the ATPase activity of Hsp70 proteins within the cytosol [28]. Thus increased expression of Dnajb1 proteins would be beneficial by promoting Hsp70 activation and protection against heat stress, as is the case with TOL mice. Interestingly, other studies have associated Dnajb1expression to tumor suppression in certain types of lung cancer [29].

Small Hsps, such as the Hspb family, are thought to be involved in cellular pathways accommodating protein folding and degradation [30], [31]. Members of the Hspb family interact with Hsp70 proteins and mainly serve as chaperones and/or protectors of the cytoskeleton [30], [32]. Differential expression of Hspb7 proteins, which localize within the cytosol and associate with myofibrils in skeletal and cardiac muscle cells [33], [34], has been associated with conditions such as sporadic heart failure [35] and acute coronary syndrome [36]. In the present study we showed Hspb7 gene expression to be preferentially down-regulated in skeletal muscle of TOL mice. As demonstrated previously, heat stress is associated with a tremendous increase in cardiac workload [16]. Thus, it is possible that Hspb7 expression in the heart may have a role in heat tolerance. Examining the hearts of TOL and INT mice for shared biochemical pathways as we did for skeletal muscle would be needed to clarify this possibility.

Single stranded, non-protein coding, small RNAs known as miRNAs have emerged as critical regulators of cell differentiation, identity and maintenance [37], [38]. As part of the RNA-induced silencing complex (RISC) miRNAs target mRNA transcripts mainly within the 3′UTR to promote mRNA degradation and/or translational repression [39]. Nucleotides 2–8 from the 5′ end of the mature miRNA (seed sequence region) are important for targeting mRNA [40]. Each miRNA can target up to hundreds (or thousands) of mRNAs *in vivo* and therefore potentially regulate multiple biological pathways [41]. Recent research studies have confirmed that in certain conditions miRNAs have the ability to regulate the expression levels of Hsps such as Hsp60 and Hsp70 in cardiomyocytes and skeletal muscle respectively [42], [43]. As such we conducted miRNA expression microarrays on our gastrocnemius muscle tissue samples to examine other mechanisms involved in regulating heat tolerance. Sixty-one distinct miRNAs differed significantly between TOL and INT mice (Table S2). However, all miRNA had less than a 1.3-fold difference in expression. Integration of these data (Partek analysis) with the mRNA microarray data identified 8 miRNA seed sequences that corresponded significantly to the Hsps listed in Figure 3B. Only seed sequences for miR-199a-3p and miR-34a-5p targeted the top two ranked genes Hspa1a and Dnajb1 and, their respective up-regulation and down-regulation in TOL mice were validated using quantitative RT-PCR. Previous studies have linked miR-199a-3p dysregulation to tumor progression in breast and liver carcinoma [44], [45] and susceptibility to hepatocyte injury [46], whereas miR-34a-5p dysregulation has been associated with the muscle condition Myotonic Dystrophy [47]. To fully evaluate how these miRNAs specifically relate to heat tolerance/thermoregulation in skeletal muscle, their individual roles within the pathways must be studied. Examples of the pathways important for heat tolerance are shown in Figures 5 and 6.

In conclusion, our study provides a detailed investigation into the differentially expressed genes and gene networks likely to be responsible for heat tolerance/thermoregulation in skeletal muscle *in vivo*. Genes such as Hspa1a, Dnajb1 and Hspb7 have been determined to contribute the most to heat tolerance within our system. Further studies are needed to confirm the importance of such genes and their respective miRNAs in the regulation of heat tolerance. Where these same molecular pathways would generalize to human thermoregulation is under investigation. Ultimately, such information will lead to the targeted design of more effective strategies for both diagnosing and treating heat sensitive individuals pre-disposed to heat-related injuries.

## Supporting Information

Table S1 **List of 3,081 genes identified using microarray to be differentially expressed in TOL mice compared to INT mice with respective fold change and p value of significance.**
(DOCX)

Table S2 **List of 61 miRNA seed sequences identified using microarray to be differentially expressed in TOL mice compared to INT mice with respective fold change and p value of significance.**
(DOCX)
