# Supplementary material for: An Exploration of Heat Tolerance in Mice Utilizing mRNA and microRNA Expression Analysis
Source: PLoS One. 2013 Aug 15;8(8):e72258. doi: 10.1371/journal.pone.0072258 (PMC3744453; doi:10.1371/journal.pone.0072258)
Supplement: Table S1 — List of 3,081 genes identified using microarray to be differentially expressed in TOL mice compared to INT mice with respective fold change and p value of significance. (DOCX) [file pone.0072258.s001.docx]

**Table S1. List of 3,081 genes identified using microarray to be differentially expressed in TOL mice compared to INT mice with respective fold change and p value of significance.**

| **Gene ID** | **Microarray fold change** | **P value** | **Gene ID** | **Microarray fold change** | **P value** |
| --- | --- | --- | --- | --- | --- |
| \| HSPA1A \| \| --- \| \| DNAJB1 \| \| EGR2 \| \| GM1157 \| \| SMOX \| \| ERRFI1 \| \| SMOX \| \| ZFP36 \| \| SLC25A25 \| \| SMOX \| \| CYR61 \| \| SMOX \| \| SMOX \| \| ABRA \| \| IGFBP5 \| \| KCNG4 \| \| EG435391 \| \| ARC \| \| AXUD1 \| \| AMD2 \| \| PPP1R10 \| \| PPP1R10 \| \| DUPD1 \| \| LOC382058 \| \| SH3KBP1 \| \| JUN \| \| CYR61 \| \| SH3KBP1 \| \| USH2A \| \| AQP4 \| \| IGFBP5 \| \| UBG \| \| SH3KBP1 \| \| KCNG4 \| \| CYP2J6 \| \| PPARGC1A \| \| LOC100047905 \| \| PER1 \| \| TCFCP2L1 \| \| EG435391 \| \| FOSB \| \| LOC100048105 \| \| TBX15 \| \| TOB2 \| \| ANKRD23 \| \| PLK3 \| \| 8430426J06RIK \| \| IGFBP5 \| \| CD83 \| \| SNCA \| \| IL4I1 \| \| EDN3 \| \| LOC100048105 \| \| ESRRG \| \| CTGF \| \| FBN1 \| \| TAS2R121 \| \| BC064033 \| \| LIN52 \| \| PITPNC1 \| \| PPIL5 \| \| MPP3 \| \| COQ10B \| \| FN1 \| \| SLC25A30 \| \| NUPR1 \| \| C030018L16RIK \| \| TIMP1 \| \| LOC100044702 \| \| PER1 \| \| LOC100048105 \| \| LOC100041585 \| \| SLC5A8 \| \| COL1A2 \| \| NUAK2 \| \| MFAP4 \| \| ACTR3B \| \| ATP8A1 \| \| CASQ1 \| \| CLK1 \| \| CLEC5A \| \| ORC1L \| \| TIMP1 \| \| PADI2 \| \| 4632413C10RIK \| \| 8030451F13RIK \| \| AS3MT \| \| IGH-6 \| \| 7420416P09RIK \| \| LOC212813 \| \| 4833441J24RIK \| \| RSPH1 \| \| TIAM1 \| \| PI16 \| \| COL16A1 \| \| MPP3 \| \| GNAS \| \| ICA1L \| \| SLC8A3 \| \| PANX2 \| \| AA536749 \| \| FSTL1 \| \| SORL1 \| \| C230015M01RIK \| \| MAT2A \| \| PER2 \| \| 9830123M21RIK \| \| 3110062M04RIK \| \| PCDHB19 \| \| PDE7A \| \| DBN1 \| \| UBASH3A \| \| C330011K17RIK \| \| LOC224532 \| \| FAM132B \| \| IGFBP6 \| \| TMPO \| \| PER2 \| \| UPF3B \| \| SCMH1 \| \| LRRC52 \| \| RAD51L3 \| \| EG546894 \| \| KIF21A \| \| DUSP8 \| \| SNF1LK \| \| SERF1 \| \| ELA2A \| \| LOC333331 \| \| ATL2 \| \| B230217C12RIK \| \| KIF7 \| \| F11 \| \| COL6A1 \| \| OLFR386 \| \| MFAP5 \| \| EG434858 \| \| GPD1 \| \| ATP8A1 \| \| ASB14 \| \| GPR82 \| \| 4930534P07RIK \| \| LOC638935 \| \| OLFR1469 \| \| ODZ3 \| \| A730081H18RIK \| \| GPT2 \| \| IL1B \| \| MAT2A \| \| D0H4S114 \| \| 4931431C16RIK \| \| MED13 \| \| HNRNPA2B1 \| \| PPM1L \| \| BC064033 \| \| 4921538N17RIK \| \| SYNE1 \| \| SNURF \| \| 2310003H01RIK \| \| 2310005C01RIK \| \| PRSS27 \| \| OLFR47 \| \| TTC30A2 \| \| ARRB1 \| \| PTP4A2 \| \| LAG3 \| \| VASN \| \| LIPK \| \| ADK \| \| RNF122 \| \| LOC671209 \| \| CDC2L6 \| \| PHKA1 \| \| FGF1 \| \| V1RE10 \| \| A830006N08RIK \| \| CPB2 \| \| OTTMUSG00000017827 \| \| CDH19 \| \| RPGRIP1 \| \| COL6A1 \| \| GPR137B \| \| GDAP1 \| \| A030011M19 \| \| IQCA \| \| LOC245533 \| \| HIP-1 \| \| ADAM12 \| \| STBD1 \| \| SERPINE2 \| \| ATL2 \| \| MYLK2 \| \| A530098C11RIK \| \| RPS13 \| \| TECTA \| \| TSPAN11 \| \| SLC25A20 \| \| AP1M2 \| \| B230308N11RIK \| \| PPP2R5C \| \| TILZ3C \| \| A430107P09RIK \| \| FBXO3 \| \| BAI2 \| \| PCSK6 \| \| ITPR1 \| \| OLFR1138 \| \| ITGBL1 \| \| OTUD1 \| \| KCNA7 \| \| NUP54 \| \| PHKG1 \| \| NDUFS1 \| \| NUSAP1 \| \| DDX5 \| \| RGS18 \| \| PHKA1 \| \| SCL0003425.1_488 \| \| SCL000981.1_40 \| \| IL20 \| \| GRB14 \| \| GUF1 \| \| V1RC32 \| \| ABI3BP \| \| ERAP1 \| \| A930001N09RIK \| \| AR \| \| RCAN2 \| \| TCTEX1D1 \| \| EVX2 \| \| PCSK6 \| \| A630056H20RIK \| \| MAD \| \| A830093I24RIK \| \| ZXDC \| \| CSPG5 \| \| D330037H01RIK \| \| KNSL5 \| \| RNF138 \| \| B2M \| \| LOC100044165 \| \| JMJD2A \| \| ITGAM \| \| ADAM4 \| \| 2610103J23RIK \| \| RSRC2 \| \| IGH-VJ558 \| \| LOC381220 \| \| TIPIN \| \| FBXO30 \| \| 6430601O08RIK \| \| HRASLS \| \| NMUR1 \| \| LOC637776 \| \| PTH2R \| \| SGCA \| \| COVA1 \| \| A230057G18RIK \| \| PPAPDC3 \| \| BNIP2 \| \| 2310065F04RIK \| \| NUSAP1 \| \| SYNPO2 \| \| PIGO \| \| IL7R \| \| 2610301N02RIK \| \| 6330411D24RIK \| \| FBLN2 \| \| SYTL3 \| \| DEFB34 \| \| 4832428D23RIK \| \| ARG2 \| \| MAP2K6 \| \| FBXL10 \| \| ACSS3 \| \| TNFRSF9 \| \| LOC235444 \| \| PDE9A \| \| ANK2 \| \| MSI2 \| \| COL2A1 \| \| PDLIM5 \| \| RGS19IP3 \| \| A730054J21RIK \| \| CLTA \| \| IGSF1 \| \| ABCB9 \| \| KRT75 \| \| MESDC2 \| \| 1500019G21RIK \| \| CDC42SE2 \| \| HSPA2 \| \| PIM1 \| \| V1RD17 \| \| 6430544A07RIK \| \| PDS5A \| \| LOC100047082 \| \| CRY1 \| \| NEURL \| \| SCL0002690.1_1 \| \| RELN \| \| COL3A1 \| \| SLC2A4 \| \| IGHV1S120_1S120_8 \| \| 6430407L02RIK \| \| 4933407L23RIK \| \| KRT4 \| \| F630025I20RIK \| \| LOC100042952 \| \| PADI2 \| \| AFMID \| \| FBXL5 \| \| C330006D17RIK \| \| 9430028L06RIK \| \| EG433923 \| \| CYB5R4 \| \| LOC380881 \| \| LOC232885 \| \| CLCC1 \| \| PCCA \| \| SPAG9 \| \| ENAM \| \| LOC100047353 \| \| MAL \| \| DUSP7 \| \| 2410003P15RIK \| \| TMPO \| \| ZFP422-RS1 \| \| LOC384448 \| \| CLK4 \| \| EPC1 \| \| C4BP \| \| CD53 \| \| LOC100048703 \| \| PDCD10 \| \| PPBP \| \| B930059J09RIK \| \| OLFR1200 \| \| RAB11FIP5 \| \| CBFB \| \| CHPT1 \| \| LAMC3 \| \| ROCK2 \| \| V1RG5 \| \| GANC \| \| DNAJB6 \| \| LOC208080 \| \| GOSR2 \| \| THSD4 \| \| KLRB1B \| \| TOP1 \| \| 3110001A13RIK \| \| LOC381282 \| \| OXCT1 \| \| D630021C08RIK \| \| OLFM3 \| \| LOC100045678 \| \| AI747448 \| \| GDAP1 \| \| OLFR30 \| \| CACNA2D1 \| \| E330037M01RIK \| \| 1700020C11RIK \| \| 9530082I15RIK \| \| NECL1-PENDING \| \| C130064E22RIK \| \| COL12A1 \| \| ANK3 \| \| FGF21 \| \| MBD2 \| \| LOC384688 \| \| LOC100046843 \| \| ATL2 \| \| RBM14 \| \| SERPINE2 \| \| GNPDA1 \| \| CEBPE \| \| LOC240669 \| \| E130307A14RIK \| \| OLFR605 \| \| 4930573H18RIK \| \| SLC17A4 \| \| ES1 \| \| GAN \| \| RGS3 \| \| V1RC14 \| \| PAQR9 \| \| LOC380992 \| \| XRCC1 \| \| SPT1 \| \| LOC381635 \| \| B230378H13RIK \| \| STXBP5L \| \| 9530084K20RIK \| \| BCAT1 \| \| SLC25A15 \| \| CTSK \| \| CDR2 \| \| MUC10 \| \| GM410 \| \| SLC1A4 \| \| LOC329824 \| \| 3110012M05RIK \| \| IL1RL1L \| \| ANP32A \| \| LOC245600 \| \| ZFP703 \| \| 1700084C01RIK \| \| JMJD1C \| \| MYNN \| \| 9330175B01RIK \| \| FAAH \| \| CBFB \| \| SH3BGR \| \| 9630011E01RIK \| \| SETD8 \| \| LOC382069 \| \| CD44 \| \| DAPK1 \| \| MERG \| \| SNX17 \| \| HBP1 \| \| KLF12 \| \| ARL2BP \| \| STAR \| \| OSM \| \| NUP133 \| \| ANLN \| \| 2310040G07RIK \| \| OLFR1180 \| \| KRTDAP \| \| FURIN \| \| 5430434G16RIK \| \| CTF1 \| \| NR4A2 \| \| SUCLG2 \| \| DEFA1 \| \| EIF5A2 \| \| IGFALS \| \| 4632425I16RIK \| \| GGN \| \| ZFP97 \| \| B930046C15RIK \| \| TRPM7 \| \| METT5D1 \| \| LOC665566 \| \| GSTCD \| \| RNF11 \| \| DIDO1 \| \| HIVEP3 \| \| LOC383926 \| \| SPINT1 \| \| LOC676748 \| \| SFRS5 \| \| LOC100047934 \| \| KCNMA1 \| \| 9330182L06RIK \| \| ARL2BP \| \| OLFR545 \| \| 9830165L15RIK \| \| KDELR2 \| \| GYS1 \| \| LOC670389 \| \| CORO6 \| \| LRRC39 \| \| SERPINB2 \| \| RALGPS2 \| \| 6030429G01RIK \| \| GM318 \| \| 9830004G04RIK \| \| LOC272417 \| \| ZZZ3 \| \| CHES1 \| \| TCFAP2B \| \| SCL0002785.1_49 \| \| METTL6 \| \| 4732462K23RIK \| \| 4930528G09RIK \| \| AMPH \| \| GALC \| \| LOC633072 \| \| SERPINF1 \| \| CPE \| \| GM944 \| \| PPT1 \| \| EG332993 \| \| 4930542C16RIK \| \| D6ERTD474E \| \| A330104J06RIK \| \| IGHG1_1_792 \| \| 9130017K11RIK \| \| 1200003E11RIK \| \| SLC9A2 \| \| COPS3 \| \| LOC673135 \| \| AURKA \| \| C430014D17RIK \| \| 2310042D19RIK \| \| POGZ \| \| 1700113I22RIK \| \| ASH1L \| \| C230064E22RIK \| \| PALLD \| \| 1110049L02RIK \| \| 1700021P22RIK \| \| GPRASP1 \| \| ALDH1L2 \| \| ZFP473 \| \| CACNG6 \| \| EPB4.1L5 \| \| MOBKL2C \| \| 2310043I08RIK \| \| TIAF2 \| \| ENTPD4 \| \| TNFRSF6 \| \| HIST2H2BB \| \| GNAS \| \| SFT2D1 \| \| OLFR809 \| \| OLFR623 \| \| DDX6 \| \| SFXN2 \| \| TRP53INP2 \| \| FAS \| \| G630024G08RIK \| \| ANKS6 \| \| GHSR \| \| LOC637227 \| \| USP15 \| \| TAS2R140 \| \| ECHDC1 \| \| MSH3 \| \| RP1L1 \| \| GPRC5C \| \| JMJD2C \| \| COL22A1 \| \| LGMN \| \| BPNT1 \| \| LOC383602 \| \| D030017L14RIK \| \| BCKDK \| \| EHMT2 \| \| ITGA10 \| \| CAPZB \| \| ASB10 \| \| MYO10 \| \| A130064M08RIK \| \| 1700096P03RIK \| \| CYP27A1 \| \| ASF1A \| \| OLFR118 \| \| PMFBP1 \| \| GPRC5C \| \| GTLF3B \| \| LOC330074 \| \| TTC18 \| \| CDH13 \| \| OLFR684 \| \| 4932415M13RIK \| \| KLF8 \| \| 1700016B15RIK \| \| LOC233710 \| \| SEC15L1 \| \| EPHB4 \| \| SCL000490.1_983 \| \| WHRN \| \| LOC383022 \| \| ZDHHC19 \| \| DDAH1 \| \| NODAL \| \| JAK2 \| \| LUC7L2 \| \| TRRP4 \| \| SCL000530.1_2 \| \| ARSJ \| \| D130004I16RIK \| \| NUCKS1 \| \| PIGY \| \| CCDC127 \| \| A430069M06RIK \| \| 1110006E14RIK \| \| D030020N12RIK \| \| LOC239727 \| \| TMEM88 \| \| ZADH2 \| \| MTCP1 \| \| KLHL24 \| \| ARHGEF15 \| \| TCFAP2A \| \| AW551984 \| \| A130009C12RIK \| \| ATP6V1G1 \| \| ACSS2 \| \| TNFRSF11A \| \| D430007A19RIK \| \| V1RC24 \| \| AV320801 \| \| RASSF5 \| \| OLFR820 \| \| LNPEP \| \| SNN \| \| LOC381562 \| \| RAN \| \| C330024D21RIK \| \| FOXO4 \| \| GNG7 \| \| CKMT1 \| \| WWP1 \| \| ADK \| \| XKR5 \| \| SLC13A5 \| \| LOC633936 \| \| LHFP \| \| OLFR1058 \| \| DEPDC6 \| \| G630014P10RIK \| \| DNAJB4 \| \| 2310014D11RIK \| \| SPINK4 \| \| D430043M02RIK \| \| FABP1 \| \| FOXN3 \| \| LOC100045522 \| \| LOC277385 \| \| OLFR1312 \| \| LOC100048696 \| \| HMGCL \| \| BC024659 \| \| LOC676606 \| \| FN1 \| \| CNP \| \| DFFB \| \| 2610024B07RIK \| \| C85492 \| \| COMP \| \| SDCBP \| \| LOC383828 \| \| 2810423A18RIK \| \| LOC669660 \| \| PPP4R1 \| \| FMR1 \| \| EG620899 \| \| ANXA8 \| \| RNF138 \| \| MTHFD2 \| \| ZFP217 \| \| ADAM22 \| \| 8030486A12RIK \| \| 4930402F06RIK \| \| FGF17 \| \| EGLN1 \| \| PCDHA9 \| \| MSN \| \| ODF2 \| \| BCLP2 \| \| 9430032E06RIK \| \| ATP5J \| \| LOC100045185 \| \| NUDC \| \| 1110004P21RIK \| \| EEF1A2 \| \| NRBP1 \| \| V1RC26 \| \| LOC386423 \| \| TYMS \| \| CYB5B \| \| AHCYL1 \| \| LOC232065 \| \| GM884 \| \| C030032F19RIK \| \| KIF13A \| \| 1300010M03RIK \| \| IL15 \| \| VRK1 \| \| DAPP1 \| \| ABCC4 \| \| LONRF1 \| \| HEXIM1 \| \| A730013G04 \| \| EG624138 \| \| NTN4 \| \| NPHS1 \| \| ABCB8 \| \| H2-DMA \| \| NFE2L2 \| \| SCL000031.1_6_COMP \| \| WDR1 \| \| SSPO \| \| PROC \| \| LOC100048042 \| \| JAK2 \| \| SPAM1 \| \| SLC41A3 \| \| 3930401K13RIK \| \| FCER2A \| \| SSX2IP \| \| GTRGEO22 \| \| EGLN1 \| \| MFAP2 \| \| OLFR1316 \| \| LOC383005 \| \| RASEF \| \| PALLD \| \| DPCR1 \| \| AK162044 \| \| RASGRP1 \| \| PITPNM3 \| \| LOC383057 \| \| ORAI3 \| \| DAPK1 \| \| MAPK6 \| \| 1700019H03RIK \| \| LOC381340 \| \| OLFR1361 \| \| PPM1L \| \| BTBD10 \| \| CHD1 \| \| KLHL8 \| \| ALS2CR13 \| \| A530052K23RIK \| \| NOS1 \| \| PRF1 \| \| GNB5 \| \| LOC381089 \| \| TMEM65 \| \| EG622384 \| \| AGXT2L1 \| \| PIK3CD \| \| MIER3 \| \| SCL0001883.1_112 \| \| SAE1 \| \| C230094L10RIK \| \| A530060J09RIK \| \| ABCD3 \| \| EPHB1 \| \| TTC19 \| \| GTSE1 \| \| AKAP2 \| \| LOC268800 \| \| BRD2 \| \| 4732418C07RIK \| \| DNAJC27 \| \| GCC1 \| \| MAML1 \| \| 4930511I11RIK \| \| LOC100047856 \| \| TUBD1 \| \| LOC100046690 \| \| 6430573H23RIK \| \| CSF3R \| \| ABCF2 \| \| MAPK6 \| \| PCDHA7 \| \| ASCL1 \| \| PFKM \| \| LOC546994 \| \| MB \| \| LOC384742 \| \| AW822252 \| \| LOC236251 \| \| 2810409K11RIK \| \| GRM7 \| \| A330021D07RIK \| \| RBMS3 \| \| H13 \| \| 6230424H07RIK \| \| SIRT1 \| \| PRMT7 \| \| CLEC5A \| \| EG631624 \| \| LOC382493 \| \| SLC12A7 \| \| LOC100038898 \| \| LPHN1 \| \| 9630015D15RIK \| \| TSSC4 \| \| TSPAN18 \| \| TINAG \| \| LOC671784 \| \| SLC13A4 \| \| D0H8S2298E \| \| PPFIA1 \| \| ZFP780B \| \| A830007N20RIK \| \| CCR5 \| \| GJC2 \| \| ARHGEF6 \| \| SPRYD4 \| \| AHSG \| \| PABPC4 \| \| 5230401M06RIK \| \| KCNQ3 \| \| SCUBE2 \| \| D230017B08RIK \| \| OLFR98 \| \| APLP2 \| \| TOR1AIP1 \| \| FBN1 \| \| OLFR1373 \| \| OLFR934 \| \| ST3GAL1 \| \| SEL1L2 \| \| HOXA1 \| \| LHFPL3 \| \| 5330439C02RIK \| \| 1110059G10RIK \| \| A030007L17RIK \| \| TUBB4 \| \| LOC100046163 \| \| 2310044D20RIK \| \| OGT \| \| LOC385630 \| \| LOC225924 \| \| HIF1AN \| \| 2610021K21RIK \| \| PIASG-PENDING \| \| AGXT \| \| DNAJC27 \| \| EEF2K \| \| BHLHB8 \| \| LOC100048163 \| \| SCL0001534.1_16 \| \| LOC382405 \| \| OLFR1104 \| \| MKIAA0864 \| \| TRMT2B \| \| VIT \| \| PPOX \| \| SURF6 \| \| D830007F02RIK \| \| CHD5 \| \| GABRG3 \| \| 5730417B17RIK \| \| COL12A1 \| \| PYCR1 \| \| KCNN3 \| \| OLFR707 \| \| DGKK \| \| EG386506 \| \| SNTG1 \| \| 4930471G03RIK \| \| JAK3 \| \| SCL0002066.1_0 \| \| SERPINA3H \| \| LPHN3 \| \| TPK1 \| \| MIA3 \| \| ARRB1 \| \| BC038156 \| \| B4GALT6 \| \| HDAC9 \| \| PNRC2 \| \| GALNT1 \| \| 9330160M17RIK \| \| SBF2 \| \| CPEB3 \| \| 1110017D15RIK \| \| ADAMTS12 \| \| GM732 \| \| SCL0003176.1_43 \| \| CASP9 \| \| DCC \| \| EGLN1 \| \| IREBF1 \| \| 9530080O11RIK \| \| CKMT2 \| \| INSIG2 \| \| UBE2N \| \| 4921523A10RIK \| \| A730008I18RIK \| \| AA408296 \| \| MJ-5000-153_4699 \| \| HPN \| \| PDSS2 \| \| KLF13 \| \| D430018F24RIK \| \| ENPP4 \| \| JMJD2A \| \| EGFLAM \| \| KCNJ15 \| \| 9330210B09RIK \| \| TMEM23 \| \| 1700095J19RIK \| \| 1810012K16RIK \| \| SCL0001416.1_21 \| \| RXRG \| \| HPCAL1 \| \| PELI2 \| \| SCN3A \| \| LOC100045737 \| \| FUNDC2 \| \| ADAMTSL2 \| \| CACNB3 \| \| RAPSN \| \| SLC6A4 \| \| TRMU \| \| 8430401F14RIK \| \| LOC385953 \| \| LRRC20 \| \| 2210016L21RIK \| \| 1200003I07RIK \| \| ADAMTS14 \| \| LYNX1 \| \| E130012A19RIK \| \| BB128963 \| \| CXCR5 \| \| TUBB2B \| \| PRL8A9 \| \| 4631405J19RIK \| \| V1RD7 \| \| SCL0003073.1_164 \| \| 9430029L20RIK \| \| GULP1 \| \| MPDZ \| \| TBC1D9 \| \| 4930452B06RIK \| \| LRIG1 \| \| GM867 \| \| GPR133 \| \| SNF1LK2 \| \| LOC384313 \| \| OSTM1 \| \| V1RD18 \| \| STIM1 \| \| D930007B01RIK \| \| 6430537F04 \| \| PIP4K2B \| \| D030070I18RIK \| \| 9030003C19RIK \| \| CCNT2 \| \| HIF3A \| \| LOC386416 \| \| GALNT1 \| \| LOC386553 \| \| EG545886 \| \| GPRC2A-RS5 \| \| LOC242235 \| \| 2310046A06RIK \| \| 6820406G21RIK \| \| OLFR281 \| \| LOC380916 \| \| OLA1 \| \| BAT2 \| \| LOC229494 \| \| COL4A4 \| \| 4932702M20RIK \| \| PIWIL1 \| \| CDC37 \| \| TIMP3 \| \| 2410013I23RIK \| \| 4933434E20RIK \| \| 4931440L10RIK \| \| LOC237195 \| \| RBBP2 \| \| A930023F05RIK \| \| AI593442 \| \| NKAIN2 \| \| SNRPD3 \| \| MIF4GD \| \| ERC1 \| \| OLFR702 \| \| COL9A2 \| \| JMJD6 \| \| ACAD9 \| \| DGKB \| \| BC026996 \| \| TAGAP1 \| \| SOX11 \| \| MCL1 \| \| ZFP575 \| \| PDHA1 \| \| CNOT1 \| \| 6430578G21RIK \| \| TTC32 \| \| 1500005C15RIK \| \| RNF138 \| \| PDZK1 \| \| WNT2 \| \| EXOC4 \| \| ALDH1L2 \| \| LMAN2L \| \| A730041D04RIK \| \| LOC385361 \| \| PCDH20 \| \| 4933421E11RIK \| \| LOC100044683 \| \| LOC240672 \| \| CDKN2AIP \| \| MOR, GPCR \| \| MRPL48 \| \| LOC380854 \| \| SETD8 \| \| RCAN2 \| \| LOC676222 \| \| LMAN2L \| \| 1110001N06RIK \| \| 3930401E15RIK \| \| LOC268782 \| \| EG625660 \| \| ZFP185 \| \| B230353O14RIK \| \| ZCCHC14 \| \| TIGD4 \| \| AGL \| \| ZFP46 \| \| LOC384649 \| \| OLFR951 \| \| TRIM69 \| \| GLS \| \| ADCY2 \| \| CD247 \| \| 5730585A16RIK \| \| LAMP2 \| \| FASL \| \| 9630055A16RIK \| \| 4921517D16RIK \| \| NCOA3 \| \| FKBP9 \| \| REEP1 \| \| GIT2 \| \| DOCK10 \| \| SCL0001199.1_86 \| \| AY358078 \| \| LOC383630 \| \| CLASP1 \| \| B3GALNT2 \| \| INSIG2 \| \| CRHR1 \| \| A630043I21RIK \| \| BMP1 \| \| TCFE2A \| \| 9330176N13RIK \| \| 9030625A04RIK \| \| E430021P16RIK \| \| D4WSU132E \| \| BC024868 \| \| KLHDC5 \| \| LOC621968 \| \| CD19 \| \| ZFP597 \| \| RBM3 \| \| STRA13 \| \| LOC385718 \| \| ITK \| \| DST \| \| SPINLW1 \| \| ERN1 \| \| TTC19 \| \| PRKCZ \| \| LOC381395 \| \| NDFIP1 \| \| D630003M21RIK \| \| NUDT12 \| \| KREMEN-PENDING \| \| SLC45A1 \| \| MSRB2 \| \| SKAP1 \| \| CENPM \| \| PRKCQ \| \| LOC245187 \| \| MCCC2 \| \| C130069I09RIK \| \| D630008I10RIK \| \| LOC380934 \| \| LOC385742 \| \| FCGR2B \| \| GALNT1 \| \| IL27RA \| \| E430003H02RIK \| \| TRIM35 \| \| 4930402E16RIK \| \| 9330134C04RIK \| \| POMT1 \| \| 2900011L18RIK \| \| SYNGR1 \| \| ZFP787 \| \| ATP10A \| \| SCL0002720.1_68 \| \| CD19 \| \| RASSF2 \| \| MED26 \| \| HERPUD1 \| \| LYPD5 \| \| LOC382065 \| \| GRIK1 \| \| SH3BP1 \| \| UNC5C \| \| AQP1 \| \| SETDB1 \| \| MED14 \| \| SCL000650.1_5 \| \| ETV5 \| \| MAF \| \| LOC380641 \| \| URM1 \| \| MRPL48 \| \| LOC432945 \| \| IL15RA \| \| FBXL5 \| \| OLFR215 \| \| A730085F06RIK \| \| C87414 \| \| A430076E10RIK \| \| ENSMUSG00000043795 \| \| LOC384280 \| \| 0710001D07RIK \| \| 9626978_121 \| \| ANKRD22 \| \| SEMA3B \| \| AGPAT1 \| \| LPCAT3 \| \| LOC100045284 \| \| RILPL1 \| \| ANUBL1 \| \| DOLPP1 \| \| SMAD5 \| \| FASTK \| \| LMO7 \| \| BCKDK \| \| IGHV1S114_205 \| \| UBE2I \| \| HERPUD1 \| \| TUSC5 \| \| LOC100047260 \| \| HAPLN4 \| \| ACO2 \| \| PEX7 \| \| FNIP1 \| \| RAB4A \| \| TBL1XR1 \| \| TAC1 \| \| CCDC50 \| \| N6AMT1 \| \| 2410004N05RIK \| \| 5830403M04RIK \| \| RWDD2 \| \| HS6ST1 \| \| IRAK \| \| 2500001D14RIK \| \| PPFIA1 \| \| A230097C02 \| \| NQO2 \| \| LRIG3 \| \| OLFR1475 \| \| PPP2R1A \| \| WWP1 \| \| RSDR1-PENDING \| \| 6330411I15RIK \| \| SMURF1 \| \| CLDN2 \| \| BC003993 \| \| NANOS3 \| \| MTRR \| \| PAPD4 \| \| BXDC5 \| \| SCN3B \| \| LOC381353 \| \| LOC100040573 \| \| LRRC43 \| \| CAMK2D \| \| ACE2 \| \| 9330186A19RIK \| \| ITGA4 \| \| TUBB2A \| \| FGFR2 \| \| DAPK1 \| \| E030016N13RIK \| \| BIRC2 \| \| LOC100047043 \| \| 1110001N06RIK \| \| MAP2K4 \| \| SKIV2L \| \| SCL0002064.1_2 \| \| DMTF1 \| \| SEMA7A \| \| A330084H10RIK \| \| KATNAL1 \| \| GSS \| \| NDUFS4 \| \| LSM14B \| \| SFRS14 \| \| DHRS9 \| \| TRPV5 \| \| GGNBP2 \| \| NSUN4 \| \| MAST4 \| \| ZFP354B \| \| LOC100036521 \| \| CEP135 \| \| LIPG \| \| CAST \| \| EPHA6 \| \| A730037L19RIK \| \| CKMT2 \| \| A830054O07RIK \| \| XPO7 \| \| 1110049B09RIK \| \| LOC100048101 \| \| RAI14 \| \| PARP1 \| \| RNMT \| \| OGFOD2 \| \| B230337C21RIK \| \| CHD9 \| \| NCKIPSD \| \| ELOVL1 \| \| SCUBE1 \| \| GANC \| \| GARNL4 \| \| RNF126 \| \| EG668725 \| \| AMBRA1 \| \| 1110031I02RIK \| \| LCE1C \| \| UTP6 \| \| ATP8B1 \| \| DPH3 \| \| 2610209A20RIK \| \| LOC384737 \| \| HOMER1 \| \| OLFR1154 \| \| DOCK1 \| \| POLR3A \| \| HSPA2 \| \| LOC210245 \| \| AUH \| \| TM7SF3 \| \| BC046331 \| \| EIF3B \| \| STK17B \| \| DHRS7B \| \| ERGIC3 \| \| OCRL \| \| PITPNA \| \| DGCR6 \| \| SCL0004029.1_17 \| \| SNURF \| \| 5830431I15RIK \| \| HMOX2 \| \| RBX1 \| \| PTBP2 \| \| AGL \| \| D030074E01RIK \| \| TRIM2 \| \| MYCBP \| \| 2010109I03RIK \| \| 9330154M19RIK \| \| PTGDS2 \| \| 0610037L13RIK \| \| LOC380623 \| \| UBR1 \| \| ANK \| \| ANK2 \| \| MFSD11 \| \| MYO5A \| \| 2410025L10RIK \| \| SCN7A \| \| 6430704M03RIK \| \| 1700015E05RIK \| \| 6430567L13RIK \| \| FPR-RS7 \| \| CACNA1A \| \| ATG10 \| \| SUFU \| \| BC048546 \| \| EPC1 \| \| FIS1 \| \| CRRY \| \| TMPRSS5 \| \| DUSP21 \| \| PAK1 \| \| CCNT1 \| \| DCAKD \| \| LOC384888 \| \| DSEL \| \| NRF1 \| \| 1300018J18RIK \| \| SHD \| \| RHEB \| \| VDAC1 \| \| MPP1 \| \| SFRS12IP1 \| \| LOC381431 \| \| OLFR549 \| \| LOC381589 \| \| SCL0001489.1_43 \| \| RAB3C \| \| SRPK2 \| \| SCL0002130.1_20 \| \| RNF5 \| \| TEX13 \| \| LOC217591 \| \| WINS2 \| \| D030051N19RIK \| \| AI854703 \| \| LOC386079 \| \| A430082A11RIK \| \| B230107K20RIK \| \| SCOC \| \| BC057079 \| \| EG623818 \| \| ERC1 \| \| RAB11FIP4 \| \| DNM1L \| \| GPR125 \| \| OLFR907 \| \| CLEC4N \| \| LOC100041835 \| \| PET112L \| \| AVPI1 \| \| FDX1 \| \| ZCCHC2 \| \| PCCB \| \| DYRK3 \| \| HBP1 \| \| GANC \| \| C130035G06RIK \| \| MID1 \| \| 0610010F05RIK \| \| TRIM66 \| \| 4933411D12RIK \| \| RPA2 \| \| VPS16 \| \| HOXC9 \| \| SLC30A8 \| \| E330019N15RIK \| \| RASA2 \| \| LRRK2 \| \| USP52 \| \| GBL \| \| LOC667185 \| \| COX7A1 \| \| MTCP1 \| \| MFN2 \| \| LOC383388 \| \| 5730453I16RIK \| \| LOC386567 \| \| LOC244219 \| \| OXR1 \| \| NUPL1 \| \| ZC3H7B \| \| 3830406C13RIK \| \| EDG2 \| \| A230010G09RIK \| \| PSAP \| \| TCF25 \| \| PARP1 \| \| CCDC56 \| \| OLFR639 \| \| 4931408A02RIK \| \| DUSP11 \| \| OGFOD1 \| \| NFAT5 \| \| TAF12 \| \| KHDRBS1 \| \| LOC620678 \| \| FOXM1 \| \| 2310005E10RIK \| \| E130307D12 \| \| SCL0001546.1_9 \| \| GTF2H2 \| \| NDUFC1 \| \| TTN \| \| CSF3R \| \| E430026H10RIK \| \| B130065L01 \| \| PDCD5 \| \| 8430415N23RIK \| \| D15ERTD621E \| \| RAB5A \| \| NDUFA4 \| \| TAOK1 \| \| B130038B15RIK \| \| WBP2 \| \| 0710001C05RIK \| \| EIF4G1 \| \| EG623230 \| \| V1RC12 \| \| MYOM1 \| \| A930009E11RIK \| \| SFXN5 \| \| KIF9 \| \| POLG2 \| \| LOC100045950 \| \| FAHD2A \| \| PPP4R1 \| \| CST11 \| \| SNTB1 \| \| 3321401G04RIK \| \| 4933433P14RIK \| \| FRS2 \| \| NDUFA2 \| \| MINK1 \| \| UQCRC2 \| \| TMEM9B \| \| MAPBPIP-PENDING \| \| RNF8 \| \| CRBN \| \| OLFR1436 \| \| ATP6V0B \| \| 2810407C02RIK \| \| ATP5B \| \| ZDHHC6 \| \| RPL23 \| \| PCNP \| \| MED27 \| \| ORC5L \| \| LOC383364 \| \| P4HB \| \| LOC100047155 \| \| PLEC1 \| \| CDYL \| \| LOC100048445 \| \| AURKAIP1 \| \| ABCA3 \| \| ZBTB8OS \| \| RUVBL1 \| \| LOC623568 \| \| NCOA5 \| \| EIF4ENIF1 \| \| 6446580_669 \| \| 1300010F03RIK \| \| RPL34 \| \| 3110045I18RIK \| \| LOC230253 \| \| EG226601 \| \| 9330134C04RIK \| \| FBXO31 \| \| 4632408I12RIK \| \| GIMAP4 \| \| CLCN4-2 \| \| PPP2R5D \| \| LOC632022 \| \| POLL \| \| ILF3 \| \| RAI12 \| \| A430005L14RIK \| \| SILG111 \| \| A530041M22RIK \| \| MYEOV2 \| \| TSPAN8 \| \| ZFP207 \| \| GRPEL1 \| \| 1810020D17RIK \| \| RPS26 \| \| MED4 \| \| FXC1 \| \| PSMB4 \| \| 1110029L17RIK \| \| TSGA10 \| \| LLPH \| \| LOC100039649 \| \| RPL41 \| \| COPE \| \| WDR46 \| \| MRPL33 \| \| A130004B21RIK \| \| MIA3 \| \| SREBF2 \| \| SNRPA \| \| LOC385905 \| \| HS6ST1 \| \| SUPT4H1 \| \| USP47 \| \| ELAC1 \| \| 0610011I19RIK \| \| RTP4 \| \| MGAT3 \| \| LOC672866 \| \| LRRC26 \| \| OLFR744 \| \| CLPP \| \| ABCF3 \| \| LOC100045802 \| \| 1700054E11RIK \| \| AI597468 \| \| TRAM1 \| \| 6620401K05RIK \| \| FRS3 \| \| RFTN2 \| \| LOC100048803 \| \| CDH15 \| \| DPH1 \| \| EAPP \| \| RPL24 \| \| GM1587 \| \| PSMA2 \| \| 4632427E13RIK \| \| LOC232890 \| \| 1500011H22RIK \| \| VRK3 \| \| RPL28 \| \| CCDC32 \| \| BEAN \| \| DNAJC1 \| \| AA408296 \| \| MON1B \| \| TSEN2 \| \| TMEM205 \| \| A230028O05RIK \| \| MED15 \| \| PNMA1 \| \| IMPDH2 \| \| EXOSC8 \| \| UBAP2L \| \| PROM1 \| \| 9330104G04RIK \| \| LOC381046 \| \| MAN1B1 \| \| NAALADL1 \| \| 1110049F12RIK \| \| UBR7 \| \| 2700046G09RIK \| \| SCL0001544.1_68 \| \| RNF26 \| \| IPO13 \| \| IGHV10S3_IG_10S3_9 \| \| POLD4 \| \| LOC381212 \| \| LOC386121 \| \| SCL0001651.1_229 \| \| DDX5 \| \| RNF181 \| \| C230046E07RIK \| \| 2810055G20RIK \| \| ECE2 \| \| RPS19 \| \| DES \| \| B3GNT3 \| \| UNCX4.1 \| \| BRP16 \| \| D12ERTD647E \| \| 6720456B07RIK \| \| MRPS30 \| \| ARHGEF2 \| \| 1810026J23RIK \| \| LOC385395 \| \| AP1M2 \| \| SERINC2 \| \| ZSCAN21 \| \| 2310079F23RIK \| | \| 8.5 \| \| --- \| \| 5.7 \| \| 3.8 \| \| 3.1 \| \| 2.9 \| \| 2.7 \| \| 2.6 \| \| 2.5 \| \| 2.5 \| \| 2.5 \| \| 2.5 \| \| 2.3 \| \| 2.3 \| \| 2.2 \| \| 2.1 \| \| 2.1 \| \| 2.0 \| \| 2.0 \| \| 2.0 \| \| 2.0 \| \| 2.0 \| \| 1.9 \| \| 1.9 \| \| 1.9 \| \| 1.9 \| \| 1.9 \| \| 1.8 \| \| 1.8 \| \| 1.8 \| \| 1.8 \| \| 1.8 \| \| 1.8 \| \| 1.8 \| \| 1.8 \| \| 1.8 \| \| 1.7 \| \| 1.7 \| \| 1.7 \| \| 1.7 \| \| 1.7 \| \| 1.6 \| \| 1.6 \| \| 1.6 \| \| 1.6 \| \| 1.6 \| \| 1.6 \| \| 1.6 \| \| 1.6 \| \| 1.6 \| \| 1.6 \| \| 1.6 \| \| 1.6 \| \| 1.6 \| \| 1.6 \| \| 1.6 \| \| 1.6 \| \| 1.6 \| \| 1.6 \| \| 1.6 \| \| 1.6 \| \| 1.6 \| \| 1.6 \| \| 1.6 \| \| 1.6 \| \| 1.6 \| \| 1.6 \| \| 1.5 \| \| 1.5 \| \| 1.5 \| \| 1.5 \| \| 1.5 \| \| 1.5 \| \| 1.5 \| \| 1.5 \| \| 1.5 \| \| 1.5 \| \| 1.5 \| \| 1.5 \| \| 1.5 \| \| 1.5 \| \| 1.5 \| \| 1.5 \| \| 1.5 \| \| 1.5 \| \| 1.5 \| \| 1.5 \| \| 1.5 \| \| 1.5 \| \| 1.5 \| \| 1.5 \| \| 1.5 \| \| 1.5 \| \| 1.5 \| \| 1.5 \| \| 1.5 \| \| 1.5 \| \| 1.5 \| \| 1.5 \| \| 1.5 \| \| 1.5 \| \| 1.5 \| \| 1.5 \| \| 1.5 \| \| 1.5 \| \| 1.5 \| \| 1.5 \| \| 1.5 \| \| 1.5 \| \| 1.5 \| \| 1.5 \| \| 1.5 \| \| 1.5 \| \| 1.5 \| \| 1.5 \| \| 1.5 \| \| 1.5 \| \| 1.5 \| \| 1.5 \| \| 1.5 \| \| 1.5 \| \| 1.5 \| \| 1.5 \| \| 1.5 \| \| 1.5 \| \| 1.5 \| \| 1.5 \| \| 1.5 \| \| 1.5 \| \| 1.5 \| \| 1.5 \| \| 1.5 \| \| 1.5 \| \| 1.5 \| \| 1.5 \| \| 1.5 \| \| 1.5 \| \| 1.5 \| \| 1.5 \| \| 1.5 \| \| 1.5 \| \| 1.5 \| \| 1.4 \| \| 1.4 \| \| 1.4 \| \| 1.4 \| \| 1.4 \| \| 1.4 \| \| 1.4 \| \| 1.4 \| \| 1.4 \| \| 1.4 \| \| 1.4 \| \| 1.4 \| \| 1.4 \| \| 1.4 \| \| 1.4 \| \| 1.4 \| \| 1.4 \| \| 1.4 \| \| 1.4 \| \| 1.4 \| \| 1.4 \| \| 1.4 \| \| 1.4 \| \| 1.4 \| \| 1.4 \| \| 1.4 \| \| 1.4 \| \| 1.4 \| \| 1.4 \| \| 1.4 \| \| 1.4 \| \| 1.4 \| \| 1.4 \| \| 1.4 \| \| 1.4 \| \| 1.4 \| \| 1.4 \| \| 1.4 \| \| 1.4 \| \| 1.4 \| \| 1.4 \| \| 1.4 \| \| 1.4 \| \| 1.4 \| \| 1.4 \| \| 1.4 \| \| 1.4 \| \| 1.4 \| \| 1.4 \| \| 1.4 \| \| 1.4 \| \| 1.4 \| \| 1.4 \| \| 1.4 \| \| 1.4 \| \| 1.4 \| \| 1.4 \| \| 1.4 \| \| 1.4 \| \| 1.4 \| \| 1.4 \| \| 1.4 \| \| 1.4 \| \| 1.4 \| \| 1.4 \| \| 1.4 \| \| 1.4 \| \| 1.4 \| \| 1.4 \| \| 1.4 \| \| 1.4 \| \| 1.4 \| \| 1.4 \| \| 1.4 \| \| 1.4 \| \| 1.4 \| \| 1.4 \| \| 1.4 \| \| 1.4 \| \| 1.4 \| \| 1.4 \| \| 1.4 \| \| 1.4 \| \| 1.4 \| \| 1.4 \| \| 1.4 \| \| 1.4 \| \| 1.4 \| \| 1.4 \| \| 1.4 \| \| 1.4 \| \| 1.4 \| \| 1.4 \| \| 1.4 \| \| 1.4 \| \| 1.4 \| \| 1.4 \| \| 1.4 \| \| 1.4 \| \| 1.4 \| \| 1.4 \| \| 1.4 \| \| 1.4 \| \| 1.4 \| \| 1.4 \| \| 1.4 \| \| 1.4 \| \| 1.4 \| \| 1.4 \| \| 1.4 \| \| 1.4 \| \| 1.4 \| \| 1.4 \| \| 1.4 \| \| 1.4 \| \| 1.4 \| \| 1.4 \| \| 1.4 \| \| 1.4 \| \| 1.4 \| \| 1.4 \| \| 1.4 \| \| 1.4 \| \| 1.4 \| \| 1.4 \| \| 1.4 \| \| 1.4 \| \| 1.4 \| \| 1.4 \| \| 1.4 \| \| 1.4 \| \| 1.4 \| \| 1.4 \| \| 1.4 \| \| 1.4 \| \| 1.4 \| \| 1.4 \| \| 1.4 \| \| 1.4 \| \| 1.4 \| \| 1.4 \| \| 1.4 \| \| 1.4 \| \| 1.4 \| \| 1.4 \| \| 1.4 \| \| 1.4 \| \| 1.4 \| \| 1.4 \| \| 1.4 \| \| 1.4 \| \| 1.4 \| \| 1.4 \| \| 1.4 \| \| 1.3 \| \| 1.3 \| \| 1.3 \| \| 1.3 \| \| 1.3 \| \| 1.3 \| \| 1.3 \| \| 1.3 \| \| 1.3 \| \| 1.3 \| \| 1.3 \| \| 1.3 \| \| 1.3 \| \| 1.3 \| \| 1.3 \| \| 1.3 \| \| 1.3 \| \| 1.3 \| \| 1.3 \| \| 1.3 \| \| 1.3 \| \| 1.3 \| \| 1.3 \| \| 1.3 \| \| 1.3 \| \| 1.3 \| \| 1.3 \| \| 1.3 \| \| 1.3 \| \| 1.3 \| \| 1.3 \| \| 1.3 \| \| 1.3 \| \| 1.3 \| \| 1.3 \| \| 1.3 \| \| 1.3 \| \| 1.3 \| \| 1.3 \| \| 1.3 \| \| 1.3 \| \| 1.3 \| \| 1.3 \| \| 1.3 \| \| 1.3 \| \| 1.3 \| \| 1.3 \| \| 1.3 \| \| 1.3 \| \| 1.3 \| \| 1.3 \| \| 1.3 \| \| 1.3 \| \| 1.3 \| \| 1.3 \| \| 1.3 \| \| 1.3 \| \| 1.3 \| \| 1.3 \| \| 1.3 \| \| 1.3 \| \| 1.3 \| \| 1.3 \| \| 1.3 \| \| 1.3 \| \| 1.3 \| \| 1.3 \| \| 1.3 \| \| 1.3 \| \| 1.3 \| \| 1.3 \| \| 1.3 \| \| 1.3 \| \| 1.3 \| \| 1.3 \| \| 1.3 \| \| 1.3 \| \| 1.3 \| \| 1.3 \| \| 1.3 \| \| 1.3 \| \| 1.3 \| \| 1.3 \| \| 1.3 \| \| 1.3 \| \| 1.3 \| \| 1.3 \| \| 1.3 \| \| 1.3 \| \| 1.3 \| \| 1.3 \| \| 1.3 \| \| 1.3 \| \| 1.3 \| \| 1.3 \| \| 1.3 \| \| 1.3 \| \| 1.3 \| \| 1.3 \| \| 1.3 \| \| 1.3 \| \| 1.3 \| \| 1.3 \| \| 1.3 \| \| 1.3 \| \| 1.3 \| \| 1.3 \| \| 1.3 \| \| 1.3 \| \| 1.3 \| \| 1.3 \| \| 1.3 \| \| 1.3 \| \| 1.3 \| \| 1.3 \| \| 1.3 \| \| 1.3 \| \| 1.3 \| \| 1.3 \| \| 1.3 \| \| 1.3 \| \| 1.3 \| \| 1.3 \| \| 1.3 \| \| 1.3 \| \| 1.3 \| \| 1.3 \| \| 1.3 \| \| 1.3 \| \| 1.3 \| \| 1.3 \| \| 1.3 \| \| 1.3 \| \| 1.3 \| \| 1.3 \| \| 1.3 \| \| 1.3 \| \| 1.3 \| \| 1.3 \| \| 1.3 \| \| 1.3 \| \| 1.3 \| \| 1.3 \| \| 1.3 \| \| 1.3 \| \| 1.3 \| \| 1.3 \| \| 1.3 \| \| 1.3 \| \| 1.3 \| \| 1.3 \| \| 1.3 \| \| 1.3 \| \| 1.3 \| \| 1.3 \| \| 1.3 \| \| 1.3 \| \| 1.3 \| \| 1.3 \| \| 1.3 \| \| 1.3 \| \| 1.3 \| \| 1.3 \| \| 1.3 \| \| 1.3 \| \| 1.3 \| \| 1.3 \| \| 1.3 \| \| 1.3 \| \| 1.3 \| \| 1.3 \| \| 1.3 \| \| 1.3 \| \| 1.3 \| \| 1.3 \| \| 1.3 \| \| 1.3 \| \| 1.3 \| \| 1.3 \| \| 1.3 \| \| 1.3 \| \| 1.3 \| \| 1.3 \| \| 1.3 \| \| 1.3 \| \| 1.3 \| \| 1.3 \| \| 1.3 \| \| 1.3 \| \| 1.3 \| \| 1.3 \| \| 1.3 \| \| 1.3 \| \| 1.3 \| \| 1.3 \| \| 1.3 \| \| 1.3 \| \| 1.3 \| \| 1.3 \| \| 1.3 \| \| 1.3 \| \| 1.3 \| \| 1.3 \| \| 1.3 \| \| 1.3 \| \| 1.3 \| \| 1.3 \| \| 1.3 \| \| 1.3 \| \| 1.3 \| \| 1.3 \| \| 1.3 \| \| 1.3 \| \| 1.3 \| \| 1.3 \| \| 1.3 \| \| 1.3 \| \| 1.3 \| \| 1.3 \| \| 1.3 \| \| 1.3 \| \| 1.3 \| \| 1.3 \| \| 1.3 \| \| 1.3 \| \| 1.3 \| \| 1.3 \| \| 1.3 \| \| 1.3 \| \| 1.3 \| \| 1.3 \| \| 1.3 \| \| 1.3 \| \| 1.3 \| \| 1.3 \| \| 1.3 \| \| 1.3 \| \| 1.3 \| \| 1.3 \| \| 1.3 \| \| 1.3 \| \| 1.3 \| \| 1.3 \| \| 1.3 \| \| 1.3 \| \| 1.3 \| \| 1.3 \| \| 1.3 \| \| 1.3 \| \| 1.3 \| \| 1.3 \| \| 1.3 \| \| 1.3 \| \| 1.3 \| \| 1.3 \| \| 1.3 \| \| 1.3 \| \| 1.3 \| \| 1.3 \| \| 1.3 \| \| 1.3 \| \| 1.3 \| \| 1.3 \| \| 1.3 \| \| 1.3 \| \| 1.3 \| \| 1.3 \| \| 1.3 \| \| 1.3 \| \| 1.3 \| \| 1.3 \| \| 1.3 \| \| 1.3 \| \| 1.3 \| \| 1.3 \| \| 1.3 \| \| 1.3 \| \| 1.3 \| \| 1.3 \| \| 1.3 \| \| 1.3 \| \| 1.3 \| \| 1.3 \| \| 1.3 \| \| 1.3 \| \| 1.3 \| \| 1.3 \| \| 1.3 \| \| 1.3 \| \| 1.3 \| \| 1.3 \| \| 1.3 \| \| 1.3 \| \| 1.3 \| \| 1.3 \| \| 1.3 \| \| 1.3 \| \| 1.3 \| \| 1.3 \| \| 1.3 \| \| 1.3 \| \| 1.3 \| \| 1.3 \| \| 1.3 \| \| 1.3 \| \| 1.3 \| \| 1.3 \| \| 1.3 \| \| 1.3 \| \| 1.3 \| \| 1.3 \| \| 1.3 \| \| 1.3 \| \| 1.3 \| \| 1.3 \| \| 1.3 \| \| 1.3 \| \| 1.3 \| \| 1.3 \| \| 1.3 \| \| 1.3 \| \| 1.3 \| \| 1.3 \| \| 1.3 \| \| 1.3 \| \| 1.3 \| \| 1.3 \| \| 1.3 \| \| 1.3 \| \| 1.3 \| \| 1.3 \| \| 1.3 \| \| 1.3 \| \| 1.3 \| \| 1.3 \| \| 1.3 \| \| 1.3 \| \| 1.3 \| \| 1.3 \| \| 1.3 \| \| 1.3 \| \| 1.3 \| \| 1.3 \| \| 1.3 \| \| 1.3 \| \| 1.3 \| \| 1.3 \| \| 1.3 \| \| 1.3 \| \| 1.3 \| \| 1.3 \| \| 1.3 \| \| 1.3 \| \| 1.3 \| \| 1.3 \| \| 1.3 \| \| 1.3 \| \| 1.3 \| \| 1.3 \| \| 1.3 \| \| 1.3 \| \| 1.3 \| \| 1.3 \| \| 1.3 \| \| 1.3 \| \| 1.3 \| \| 1.3 \| \| 1.3 \| \| 1.3 \| \| 1.3 \| \| 1.3 \| \| 1.3 \| \| 1.3 \| \| 1.3 \| \| 1.3 \| \| 1.3 \| \| 1.3 \| \| 1.3 \| \| 1.3 \| \| 1.3 \| \| 1.3 \| \| 1.3 \| \| 1.3 \| \| 1.3 \| \| 1.3 \| \| 1.3 \| \| 1.3 \| \| 1.3 \| \| 1.3 \| \| 1.3 \| \| 1.3 \| \| 1.3 \| \| 1.3 \| \| 1.3 \| \| 1.3 \| \| 1.3 \| \| 1.3 \| \| 1.3 \| \| 1.3 \| \| 1.3 \| \| 1.3 \| \| 1.3 \| \| 1.3 \| \| 1.3 \| \| 1.3 \| \| 1.3 \| \| 1.3 \| \| 1.3 \| \| 1.3 \| \| 1.3 \| \| 1.3 \| \| 1.3 \| \| 1.3 \| \| 1.3 \| \| 1.3 \| \| 1.3 \| \| 1.3 \| \| 1.3 \| \| 1.3 \| \| 1.3 \| \| 1.3 \| \| 1.3 \| \| 1.3 \| \| 1.3 \| \| 1.3 \| \| 1.3 \| \| 1.3 \| \| 1.3 \| \| 1.3 \| \| 1.3 \| \| 1.3 \| \| 1.3 \| \| 1.3 \| \| 1.3 \| \| 1.3 \| \| 1.3 \| \| 1.3 \| \| 1.3 \| \| 1.3 \| \| 1.3 \| \| 1.2 \| \| 1.2 \| \| 1.2 \| \| 1.2 \| \| 1.2 \| \| 1.2 \| \| 1.2 \| \| 1.2 \| \| 1.2 \| \| 1.2 \| \| 1.2 \| \| 1.2 \| \| 1.2 \| \| 1.2 \| \| 1.2 \| \| 1.2 \| \| 1.2 \| \| 1.2 \| \| 1.2 \| \| 1.2 \| \| 1.2 \| \| 1.2 \| \| 1.2 \| \| 1.2 \| \| 1.2 \| \| 1.2 \| \| 1.2 \| \| 1.2 \| \| 1.2 \| \| 1.2 \| \| 1.2 \| \| 1.2 \| \| 1.2 \| \| 1.2 \| \| 1.2 \| \| 1.2 \| \| 1.2 \| \| 1.2 \| \| 1.2 \| \| 1.2 \| \| 1.2 \| \| 1.2 \| \| 1.2 \| \| 1.2 \| \| 1.2 \| \| 1.2 \| \| 1.2 \| \| 1.2 \| \| 1.2 \| \| 1.2 \| \| 1.2 \| \| 1.2 \| \| 1.2 \| \| 1.2 \| \| 1.2 \| \| 1.2 \| \| 1.2 \| \| 1.2 \| \| 1.2 \| \| 1.2 \| \| 1.2 \| \| 1.2 \| \| 1.2 \| \| 1.2 \| \| 1.2 \| \| 1.2 \| \| 1.2 \| \| 1.2 \| \| 1.2 \| \| 1.2 \| \| 1.2 \| \| 1.2 \| \| 1.2 \| \| 1.2 \| \| 1.2 \| \| 1.2 \| \| 1.2 \| \| 1.2 \| \| 1.2 \| \| 1.2 \| \| 1.2 \| \| 1.2 \| \| 1.2 \| \| 1.2 \| \| 1.2 \| \| 1.2 \| \| 1.2 \| \| 1.2 \| \| 1.2 \| \| 1.2 \| \| 1.2 \| \| 1.2 \| \| 1.2 \| \| 1.2 \| \| 1.2 \| \| 1.2 \| \| 1.2 \| \| 1.2 \| \| 1.2 \| \| 1.2 \| \| 1.2 \| \| 1.2 \| \| 1.2 \| \| 1.2 \| \| 1.2 \| \| 1.2 \| \| 1.2 \| \| 1.2 \| \| 1.2 \| \| 1.2 \| \| 1.2 \| \| 1.2 \| \| 1.2 \| \| 1.2 \| \| 1.2 \| \| 1.2 \| \| 1.2 \| \| 1.2 \| \| 1.2 \| \| 1.2 \| \| 1.2 \| \| 1.2 \| \| 1.2 \| \| 1.2 \| \| 1.2 \| \| 1.2 \| \| 1.2 \| \| 1.2 \| \| 1.2 \| \| 1.2 \| \| 1.2 \| \| 1.2 \| \| 1.2 \| \| 1.2 \| \| 1.2 \| \| 1.2 \| \| 1.2 \| \| 1.2 \| \| 1.2 \| \| 1.2 \| \| 1.2 \| \| 1.2 \| \| 1.2 \| \| 1.2 \| \| 1.2 \| \| 1.2 \| \| 1.2 \| \| 1.2 \| \| 1.2 \| \| 1.2 \| \| 1.2 \| \| 1.2 \| \| 1.2 \| \| 1.2 \| \| 1.2 \| \| 1.2 \| \| 1.2 \| \| 1.2 \| \| 1.2 \| \| 1.2 \| \| 1.2 \| \| 1.2 \| \| 1.2 \| \| 1.2 \| \| 1.2 \| \| 1.2 \| \| 1.2 \| \| 1.2 \| \| 1.2 \| \| 1.2 \| \| 1.2 \| \| 1.2 \| \| 1.2 \| \| 1.2 \| \| 1.2 \| \| 1.2 \| \| 1.2 \| \| 1.2 \| \| 1.2 \| \| 1.2 \| \| 1.2 \| \| 1.2 \| \| 1.2 \| \| 1.2 \| \| 1.2 \| \| 1.2 \| \| 1.2 \| \| 1.2 \| \| 1.2 \| \| 1.2 \| \| 1.2 \| \| 1.2 \| \| 1.2 \| \| 1.2 \| \| 1.2 \| \| 1.2 \| \| 1.2 \| \| 1.2 \| \| 1.2 \| \| 1.2 \| \| 1.2 \| \| 1.2 \| \| 1.2 \| \| 1.2 \| \| 1.2 \| \| 1.2 \| \| 1.2 \| \| 1.2 \| \| 1.2 \| \| 1.2 \| \| 1.2 \| \| 1.2 \| \| 1.2 \| \| 1.2 \| \| 1.2 \| \| 1.2 \| \| 1.2 \| \| 1.2 \| \| 1.2 \| \| 1.2 \| \| 1.2 \| \| 1.2 \| \| 1.2 \| \| 1.2 \| \| 1.2 \| \| 1.2 \| \| 1.2 \| \| 1.2 \| \| 1.2 \| \| 1.2 \| \| 1.2 \| \| 1.2 \| \| 1.2 \| \| 1.2 \| \| 1.2 \| \| 1.2 \| \| 1.2 \| \| 1.2 \| \| 1.2 \| \| 1.2 \| \| 1.2 \| \| 1.2 \| \| 1.2 \| \| 1.2 \| \| 1.2 \| \| 1.2 \| \| 1.2 \| \| 1.2 \| \| 1.2 \| \| 1.2 \| \| 1.2 \| \| 1.2 \| \| 1.2 \| \| 1.2 \| \| 1.2 \| \| 1.2 \| \| 1.2 \| \| 1.2 \| \| 1.2 \| \| 1.2 \| \| 1.2 \| \| 1.2 \| \| 1.2 \| \| 1.2 \| \| 1.2 \| \| 1.2 \| \| 1.2 \| \| 1.2 \| \| 1.2 \| \| 1.2 \| \| 1.2 \| \| 1.2 \| \| 1.2 \| \| 1.2 \| \| 1.2 \| \| 1.2 \| \| 1.2 \| \| 1.2 \| \| 1.2 \| \| 1.2 \| \| 1.2 \| \| 1.2 \| \| 1.2 \| \| 1.2 \| \| 1.2 \| \| 1.2 \| \| 1.2 \| \| 1.2 \| \| 1.2 \| \| 1.2 \| \| 1.2 \| \| 1.2 \| \| 1.2 \| \| 1.2 \| \| 1.2 \| \| 1.2 \| \| 1.2 \| \| 1.2 \| \| 1.2 \| \| 1.2 \| \| 1.2 \| \| 1.2 \| \| 1.2 \| \| 1.2 \| \| 1.2 \| \| 1.2 \| \| 1.2 \| \| 1.2 \| \| 1.2 \| \| 1.2 \| \| 1.2 \| \| 1.2 \| \| 1.2 \| \| 1.2 \| \| 1.2 \| \| 1.2 \| \| 1.2 \| \| 1.2 \| \| 1.2 \| \| 1.2 \| \| 1.2 \| \| 1.2 \| \| 1.2 \| \| 1.2 \| \| 1.2 \| \| 1.2 \| \| 1.2 \| \| 1.2 \| \| 1.2 \| \| 1.2 \| \| 1.2 \| \| 1.2 \| \| 1.2 \| \| 1.2 \| \| 1.2 \| \| 1.2 \| \| 1.2 \| \| 1.2 \| \| 1.2 \| \| 1.2 \| \| 1.2 \| \| 1.2 \| \| 1.2 \| \| 1.2 \| \| 1.2 \| \| 1.2 \| \| 1.2 \| \| 1.2 \| \| 1.2 \| \| 1.2 \| \| 1.2 \| \| 1.2 \| \| 1.2 \| \| 1.2 \| \| 1.2 \| \| 1.2 \| \| 1.2 \| \| 1.2 \| \| 1.2 \| \| 1.2 \| \| 1.2 \| \| 1.2 \| \| 1.2 \| \| 1.2 \| \| 1.2 \| \| 1.2 \| \| 1.2 \| \| 1.2 \| \| 1.2 \| \| 1.2 \| \| 1.2 \| \| 1.2 \| \| 1.2 \| \| 1.2 \| \| 1.2 \| \| 1.2 \| \| 1.2 \| \| 1.2 \| \| 1.2 \| \| 1.2 \| \| 1.2 \| \| 1.2 \| \| 1.2 \| \| 1.2 \| \| 1.2 \| \| 1.2 \| \| 1.2 \| \| 1.2 \| \| 1.2 \| \| 1.2 \| \| 1.2 \| \| 1.2 \| \| 1.2 \| \| 1.2 \| \| 1.2 \| \| 1.2 \| \| 1.2 \| \| 1.2 \| \| 1.2 \| \| 1.2 \| \| 1.2 \| \| 1.2 \| \| 1.2 \| \| 1.2 \| \| 1.2 \| \| 1.2 \| \| 1.2 \| \| 1.2 \| \| 1.2 \| \| 1.2 \| \| 1.2 \| \| 1.2 \| \| 1.2 \| \| 1.2 \| \| 1.2 \| \| 1.2 \| \| 1.2 \| \| 1.2 \| \| 1.2 \| \| 1.2 \| \| 1.2 \| \| 1.2 \| \| 1.2 \| \| 1.2 \| \| 1.2 \| \| 1.2 \| \| 1.2 \| \| 1.2 \| \| 1.2 \| \| 1.2 \| \| 1.2 \| \| 1.2 \| \| 1.2 \| \| 1.2 \| \| 1.2 \| \| 1.2 \| \| 1.2 \| \| 1.2 \| \| 1.2 \| \| 1.2 \| \| 1.2 \| \| 1.2 \| \| 1.2 \| \| 1.2 \| \| 1.2 \| \| 1.2 \| \| 1.2 \| \| 1.2 \| \| 1.2 \| \| 1.2 \| \| 1.2 \| \| 1.2 \| \| 1.2 \| \| 1.2 \| \| 1.2 \| \| 1.2 \| \| 1.2 \| \| 1.2 \| \| 1.2 \| \| 1.2 \| \| 1.2 \| \| 1.2 \| \| 1.2 \| \| 1.2 \| \| 1.2 \| \| 1.2 \| \| 1.2 \| \| 1.2 \| \| 1.2 \| \| 1.2 \| \| 1.2 \| \| 1.2 \| \| 1.2 \| \| 1.2 \| \| 1.2 \| \| 1.2 \| \| 1.2 \| \| 1.2 \| \| 1.2 \| \| 1.2 \| \| 1.2 \| \| 1.2 \| \| 1.2 \| \| 1.2 \| \| 1.2 \| \| 1.2 \| \| 1.2 \| \| 1.2 \| \| 1.2 \| \| 1.2 \| \| 1.2 \| \| 1.2 \| \| 1.2 \| \| 1.2 \| \| 1.2 \| \| 1.2 \| \| 1.2 \| \| 1.2 \| \| 1.2 \| \| 1.2 \| \| 1.2 \| \| 1.2 \| \| 1.2 \| \| 1.2 \| \| 1.2 \| \| 1.2 \| \| 1.2 \| \| 1.2 \| \| 1.2 \| \| 1.2 \| \| 1.2 \| \| 1.2 \| \| 1.2 \| \| 1.2 \| \| 1.2 \| \| 1.2 \| \| 1.2 \| \| 1.2 \| \| 1.2 \| \| 1.2 \| \| 1.2 \| \| 1.2 \| \| 1.2 \| \| 1.2 \| \| 1.2 \| \| 1.2 \| \| 1.2 \| \| 1.2 \| \| 1.2 \| \| 1.2 \| \| 1.2 \| \| 1.2 \| \| 1.2 \| \| 1.2 \| \| 1.2 \| \| 1.2 \| \| 1.2 \| \| 1.2 \| \| 1.2 \| \| 1.2 \| \| 1.2 \| \| 1.2 \| \| 1.2 \| \| 1.2 \| \| 1.2 \| \| 1.2 \| \| 1.1 \| \| 1.1 \| \| 1.1 \| \| 1.1 \| \| 1.1 \| \| 1.1 \| \| 1.1 \| \| 1.1 \| \| 1.1 \| \| 1.1 \| \| 1.1 \| \| 1.1 \| \| 1.1 \| \| 1.1 \| \| 1.1 \| \| 1.1 \| \| 1.1 \| \| 1.1 \| \| 1.1 \| \| 1.1 \| \| 1.1 \| \| 1.1 \| \| 1.1 \| \| 1.1 \| \| 1.1 \| \| 1.1 \| \| 1.1 \| \| 1.1 \| \| 1.1 \| \| 1.1 \| \| 1.1 \| \| 1.1 \| \| 1.1 \| \| 1.1 \| \| 1.1 \| \| 1.1 \| \| 1.1 \| \| 1.1 \| \| 1.1 \| \| 1.1 \| \| 1.1 \| \| 1.1 \| \| 1.1 \| \| 1.1 \| \| 1.1 \| \| 1.1 \| \| 1.1 \| \| 1.1 \| \| 1.1 \| \| 1.1 \| \| 1.1 \| \| 1.1 \| \| 1.1 \| \| 1.1 \| \| 1.1 \| \| 1.1 \| \| 1.1 \| \| 1.1 \| \| 1.1 \| \| 1.1 \| \| 1.1 \| \| 1.1 \| \| 1.1 \| \| 1.1 \| \| 1.1 \| \| 1.1 \| \| 1.1 \| \| 1.1 \| \| 1.1 \| \| 1.1 \| \| 1.1 \| \| 1.1 \| \| 1.1 \| \| 1.1 \| \| 1.1 \| \| 1.1 \| \| 1.1 \| \| 1.1 \| \| 1.1 \| \| 1.1 \| \| 1.1 \| \| 1.1 \| \| 1.1 \| \| 1.1 \| \| 1.1 \| \| 1.1 \| \| 1.1 \| \| 1.1 \| \| 1.1 \| \| 1.1 \| \| 1.1 \| \| 1.1 \| \| 1.1 \| \| 1.1 \| \| 1.1 \| \| 1.1 \| \| 1.1 \| \| 1.1 \| \| 1.1 \| \| 1.1 \| \| 1.1 \| \| 1.1 \| \| 1.1 \| \| 1.1 \| \| 1.1 \| \| 1.1 \| \| 1.1 \| \| 1.1 \| \| 1.1 \| \| 1.1 \| \| 1.1 \| \| 1.1 \| \| 1.1 \| \| 1.1 \| \| 1.1 \| \| 1.1 \| \| 1.1 \| \| 1.1 \| \| 1.1 \| \| 1.1 \| \| -1.1 \| \| -1.1 \| \| -1.1 \| \| -1.1 \| \| -1.1 \| \| -1.1 \| \| -1.1 \| \| -1.1 \| \| -1.1 \| \| -1.1 \| \| -1.1 \| \| -1.1 \| \| -1.1 \| \| -1.1 \| \| -1.1 \| \| -1.1 \| \| -1.1 \| \| -1.1 \| \| -1.1 \| \| -1.1 \| \| -1.1 \| \| -1.1 \| \| -1.1 \| \| -1.1 \| \| -1.1 \| \| -1.1 \| \| -1.1 \| \| -1.1 \| \| -1.1 \| \| -1.1 \| \| -1.1 \| \| -1.1 \| \| -1.1 \| \| -1.1 \| \| -1.1 \| \| -1.1 \| \| -1.1 \| \| -1.1 \| \| -1.1 \| \| -1.1 \| \| -1.1 \| \| -1.1 \| \| -1.1 \| \| -1.1 \| \| -1.1 \| \| -1.1 \| \| -1.1 \| \| -1.1 \| \| -1.1 \| \| -1.1 \| \| -1.1 \| \| -1.1 \| \| -1.1 \| \| -1.1 \| \| -1.1 \| \| -1.1 \| \| -1.1 \| \| -1.1 \| \| -1.1 \| \| -1.1 \| \| -1.1 \| \| -1.1 \| \| -1.1 \| \| -1.1 \| \| -1.1 \| \| -1.1 \| \| -1.1 \| \| -1.1 \| \| -1.1 \| \| -1.1 \| \| -1.1 \| \| -1.1 \| \| -1.1 \| \| -1.1 \| \| -1.1 \| \| -1.1 \| \| -1.1 \| \| -1.1 \| \| -1.1 \| \| -1.1 \| \| -1.1 \| \| -1.1 \| \| -1.1 \| \| -1.1 \| \| -1.1 \| \| -1.1 \| \| -1.1 \| \| -1.1 \| \| -1.1 \| \| -1.1 \| \| -1.1 \| \| -1.1 \| \| -1.1 \| \| -1.1 \| \| -1.1 \| \| -1.1 \| \| -1.1 \| \| -1.1 \| \| -1.1 \| \| -1.1 \| \| -1.1 \| \| -1.1 \| \| -1.1 \| \| -1.1 \| \| -1.1 \| \| -1.1 \| \| -1.1 \| \| -1.1 \| \| -1.1 \| \| -1.1 \| \| -1.1 \| \| -1.1 \| \| -1.1 \| \| -1.1 \| \| -1.1 \| \| -1.1 \| \| -1.1 \| \| -1.1 \| \| -1.1 \| \| -1.1 \| \| -1.1 \| \| -1.1 \| \| -1.1 \| \| -1.1 \| \| -1.1 \| \| -1.1 \| \| -1.1 \| \| -1.1 \| \| -1.1 \| \| -1.1 \| \| -1.1 \| \| -1.1 \| \| -1.1 \| \| -1.1 \| \| -1.1 \| \| -1.1 \| \| -1.1 \| \| -1.1 \| \| -1.1 \| \| -1.1 \| | \| 0.0352 \| \| --- \| \| 0.0040 \| \| 0.0256 \| \| 0.0353 \| \| 0.0002 \| \| 0.0118 \| \| 0.0023 \| \| 0.0240 \| \| 0.0014 \| \| 0.0042 \| \| 0.0491 \| \| 0.0021 \| \| 0.0025 \| \| 0.0036 \| \| 0.0015 \| \| 0.0006 \| \| 0.0004 \| \| 0.0165 \| \| 0.0111 \| \| 0.0060 \| \| 0.0191 \| \| 0.0137 \| \| 0.0009 \| \| 0.0137 \| \| 0.0009 \| \| 0.0165 \| \| 0.0217 \| \| 0.0005 \| \| 0.0470 \| \| 0.0057 \| \| 0.0053 \| \| 0.0183 \| \| 0.0001 \| \| 0.0069 \| \| 0.0280 \| \| 0.0325 \| \| 0.0131 \| \| 0.0171 \| \| 0.0197 \| \| 0.0056 \| \| 0.0227 \| \| 0.0494 \| \| 0.0020 \| \| 0.0113 \| \| 0.0018 \| \| 0.0301 \| \| 0.0086 \| \| 0.0088 \| \| 0.0381 \| \| 0.0091 \| \| 0.0196 \| \| 0.0156 \| \| 0.0200 \| \| 0.0179 \| \| 0.0129 \| \| 0.0110 \| \| 0.0011 \| \| 0.0496 \| \| 0.0209 \| \| 0.0136 \| \| 0.0365 \| \| 0.0071 \| \| 0.0100 \| \| 0.0031 \| \| 0.0113 \| \| 0.0122 \| \| 0.0137 \| \| 0.0334 \| \| 0.0226 \| \| 0.0191 \| \| 0.0187 \| \| 0.0448 \| \| 0.0034 \| \| 0.0165 \| \| 0.0023 \| \| 0.0337 \| \| 0.0191 \| \| 0.0006 \| \| 0.0177 \| \| 0.0070 \| \| 0.0104 \| \| 0.0284 \| \| 0.0348 \| \| 0.0089 \| \| 0.0005 \| \| 0.0260 \| \| 0.0114 \| \| 0.0307 \| \| 0.0298 \| \| 0.0082 \| \| 0.0474 \| \| 0.0111 \| \| 0.0002 \| \| 0.0297 \| \| 0.0148 \| \| 0.0138 \| \| 0.0499 \| \| 0.0099 \| \| 0.0072 \| \| 0.0220 \| \| 0.0018 \| \| 0.0060 \| \| 0.0181 \| \| 0.0033 \| \| 0.0039 \| \| 0.0195 \| \| 0.0038 \| \| 0.0120 \| \| 0.0021 \| \| 0.0136 \| \| 0.0009 \| \| 0.0393 \| \| 0.0002 \| \| 0.0055 \| \| 0.0306 \| \| 0.0365 \| \| 0.0177 \| \| 0.0150 \| \| 0.0141 \| \| 0.0035 \| \| 0.0444 \| \| 0.0002 \| \| 0.0429 \| \| 0.0026 \| \| 0.0146 \| \| 0.0114 \| \| 0.0374 \| \| 0.0494 \| \| 0.0294 \| \| 0.0045 \| \| 0.0396 \| \| 0.0087 \| \| 0.0364 \| \| 0.0360 \| \| 0.0315 \| \| 0.0180 \| \| 0.0089 \| \| 0.0076 \| \| 0.0023 \| \| 0.0317 \| \| 0.0130 \| \| 0.0293 \| \| 0.0207 \| \| 0.0124 \| \| 0.0305 \| \| 0.0079 \| \| 0.0026 \| \| 0.0221 \| \| 0.0011 \| \| 0.0030 \| \| 0.0160 \| \| 0.0146 \| \| 0.0397 \| \| 0.0045 \| \| 0.0367 \| \| 0.0013 \| \| 0.0227 \| \| 0.0270 \| \| 0.0434 \| \| 0.0426 \| \| 0.0289 \| \| 0.0057 \| \| 0.0049 \| \| 0.0052 \| \| 0.0186 \| \| 0.0271 \| \| 0.0000 \| \| 0.0300 \| \| 0.0159 \| \| 0.0339 \| \| 0.0021 \| \| 0.0107 \| \| 0.0247 \| \| 0.0013 \| \| 0.0235 \| \| 0.0235 \| \| 0.0004 \| \| 0.0106 \| \| 0.0205 \| \| 0.0245 \| \| 0.0320 \| \| 0.0214 \| \| 0.0125 \| \| 0.0269 \| \| 0.0055 \| \| 0.0271 \| \| 0.0021 \| \| 0.0018 \| \| 0.0128 \| \| 0.0002 \| \| 0.0299 \| \| 0.0161 \| \| 0.0481 \| \| 0.0318 \| \| 0.0412 \| \| 0.0169 \| \| 0.0392 \| \| 0.0014 \| \| 0.0251 \| \| 0.0013 \| \| 0.0062 \| \| 0.0101 \| \| 0.0014 \| \| 0.0080 \| \| 0.0295 \| \| 0.0113 \| \| 0.0247 \| \| 0.0327 \| \| 0.0237 \| \| 0.0049 \| \| 0.0074 \| \| 0.0012 \| \| 0.0042 \| \| 0.0166 \| \| 0.0458 \| \| 0.0359 \| \| 0.0372 \| \| 0.0055 \| \| 0.0018 \| \| 0.0048 \| \| 0.0095 \| \| 0.0328 \| \| 0.0293 \| \| 0.0187 \| \| 0.0311 \| \| 0.0182 \| \| 0.0271 \| \| 0.0170 \| \| 0.0286 \| \| 0.0338 \| \| 0.0028 \| \| 0.0041 \| \| 0.0353 \| \| 0.0330 \| \| 0.0089 \| \| 0.0271 \| \| 0.0061 \| \| 0.0189 \| \| 0.0452 \| \| 0.0262 \| \| 0.0434 \| \| 0.0026 \| \| 0.0114 \| \| 0.0008 \| \| 0.0020 \| \| 0.0046 \| \| 0.0203 \| \| 0.0208 \| \| 0.0164 \| \| 0.0204 \| \| 0.0030 \| \| 0.0085 \| \| 0.0091 \| \| 0.0333 \| \| 0.0040 \| \| 0.0306 \| \| 0.0296 \| \| 0.0169 \| \| 0.0066 \| \| 0.0224 \| \| 0.0207 \| \| 0.0021 \| \| 0.0348 \| \| 0.0001 \| \| 0.0013 \| \| 0.0083 \| \| 0.0251 \| \| 0.0251 \| \| 0.0215 \| \| 0.0232 \| \| 0.0017 \| \| 0.0194 \| \| 0.0442 \| \| 0.0058 \| \| 0.0239 \| \| 0.0212 \| \| 0.0039 \| \| 0.0357 \| \| 0.0289 \| \| 0.0030 \| \| 0.0063 \| \| 0.0441 \| \| 0.0146 \| \| 0.0060 \| \| 0.0344 \| \| 0.0483 \| \| 0.0255 \| \| 0.0244 \| \| 0.0031 \| \| 0.0263 \| \| 0.0209 \| \| 0.0078 \| \| 0.0214 \| \| 0.0272 \| \| 0.0083 \| \| 0.0028 \| \| 0.0496 \| \| 0.0228 \| \| 0.0259 \| \| 0.0218 \| \| 0.0251 \| \| 0.0268 \| \| 0.0116 \| \| 0.0001 \| \| 0.0002 \| \| 0.0096 \| \| 0.0178 \| \| 0.0030 \| \| 0.0107 \| \| 0.0391 \| \| 0.0112 \| \| 0.0253 \| \| 0.0096 \| \| 0.0344 \| \| 0.0414 \| \| 0.0088 \| \| 0.0382 \| \| 0.0024 \| \| 0.0113 \| \| 0.0422 \| \| 0.0144 \| \| 0.0289 \| \| 0.0202 \| \| 0.0105 \| \| 0.0000 \| \| 0.0287 \| \| 0.0475 \| \| 0.0068 \| \| 0.0449 \| \| 0.0269 \| \| 0.0025 \| \| 0.0290 \| \| 0.0085 \| \| 0.0149 \| \| 0.0010 \| \| 0.0430 \| \| 0.0065 \| \| 0.0029 \| \| 0.0246 \| \| 0.0267 \| \| 0.0276 \| \| 0.0396 \| \| 0.0156 \| \| 0.0037 \| \| 0.0186 \| \| 0.0040 \| \| 0.0159 \| \| 0.0132 \| \| 0.0113 \| \| 0.0040 \| \| 0.0058 \| \| 0.0128 \| \| 0.0193 \| \| 0.0468 \| \| 0.0113 \| \| 0.0339 \| \| 0.0421 \| \| 0.0280 \| \| 0.0169 \| \| 0.0406 \| \| 0.0101 \| \| 0.0472 \| \| 0.0105 \| \| 0.0126 \| \| 0.0193 \| \| 0.0148 \| \| 0.0098 \| \| 0.0007 \| \| 0.0084 \| \| 0.0301 \| \| 0.0289 \| \| 0.0171 \| \| 0.0284 \| \| 0.0201 \| \| 0.0176 \| \| 0.0090 \| \| 0.0133 \| \| 0.0087 \| \| 0.0465 \| \| 0.0054 \| \| 0.0269 \| \| 0.0356 \| \| 0.0029 \| \| 0.0088 \| \| 0.0133 \| \| 0.0460 \| \| 0.0030 \| \| 0.0112 \| \| 0.0341 \| \| 0.0040 \| \| 0.0316 \| \| 0.0162 \| \| 0.0110 \| \| 0.0193 \| \| 0.0354 \| \| 0.0066 \| \| 0.0320 \| \| 0.0226 \| \| 0.0347 \| \| 0.0084 \| \| 0.0153 \| \| 0.0339 \| \| 0.0013 \| \| 0.0131 \| \| 0.0363 \| \| 0.0126 \| \| 0.0079 \| \| 0.0103 \| \| 0.0134 \| \| 0.0108 \| \| 0.0403 \| \| 0.0184 \| \| 0.0260 \| \| 0.0009 \| \| 0.0028 \| \| 0.0012 \| \| 0.0233 \| \| 0.0131 \| \| 0.0227 \| \| 0.0458 \| \| 0.0169 \| \| 0.0119 \| \| 0.0332 \| \| 0.0052 \| \| 0.0322 \| \| 0.0171 \| \| 0.0224 \| \| 0.0160 \| \| 0.0021 \| \| 0.0444 \| \| 0.0220 \| \| 0.0078 \| \| 0.0182 \| \| 0.0148 \| \| 0.0288 \| \| 0.0183 \| \| 0.0328 \| \| 0.0156 \| \| 0.0015 \| \| 0.0128 \| \| 0.0104 \| \| 0.0010 \| \| 0.0461 \| \| 0.0198 \| \| 0.0447 \| \| 0.0134 \| \| 0.0115 \| \| 0.0168 \| \| 0.0082 \| \| 0.0310 \| \| 0.0412 \| \| 0.0011 \| \| 0.0140 \| \| 0.0389 \| \| 0.0000 \| \| 0.0123 \| \| 0.0259 \| \| 0.0454 \| \| 0.0278 \| \| 0.0113 \| \| 0.0250 \| \| 0.0220 \| \| 0.0082 \| \| 0.0110 \| \| 0.0221 \| \| 0.0216 \| \| 0.0173 \| \| 0.0018 \| \| 0.0202 \| \| 0.0250 \| \| 0.0255 \| \| 0.0217 \| \| 0.0010 \| \| 0.0077 \| \| 0.0307 \| \| 0.0387 \| \| 0.0121 \| \| 0.0133 \| \| 0.0033 \| \| 0.0098 \| \| 0.0321 \| \| 0.0108 \| \| 0.0077 \| \| 0.0111 \| \| 0.0385 \| \| 0.0356 \| \| 0.0043 \| \| 0.0260 \| \| 0.0061 \| \| 0.0141 \| \| 0.0094 \| \| 0.0096 \| \| 0.0077 \| \| 0.0148 \| \| 0.0199 \| \| 0.0405 \| \| 0.0029 \| \| 0.0378 \| \| 0.0020 \| \| 0.0431 \| \| 0.0054 \| \| 0.0096 \| \| 0.0182 \| \| 0.0139 \| \| 0.0374 \| \| 0.0098 \| \| 0.0130 \| \| 0.0444 \| \| 0.0458 \| \| 0.0186 \| \| 0.0431 \| \| 0.0193 \| \| 0.0158 \| \| 0.0112 \| \| 0.0165 \| \| 0.0247 \| \| 0.0271 \| \| 0.0126 \| \| 0.0321 \| \| 0.0454 \| \| 0.0389 \| \| 0.0018 \| \| 0.0017 \| \| 0.0370 \| \| 0.0233 \| \| 0.0310 \| \| 0.0255 \| \| 0.0051 \| \| 0.0053 \| \| 0.0273 \| \| 0.0419 \| \| 0.0072 \| \| 0.0316 \| \| 0.0046 \| \| 0.0478 \| \| 0.0481 \| \| 0.0069 \| \| 0.0039 \| \| 0.0233 \| \| 0.0241 \| \| 0.0256 \| \| 0.0392 \| \| 0.0366 \| \| 0.0353 \| \| 0.0410 \| \| 0.0315 \| \| 0.0111 \| \| 0.0284 \| \| 0.0409 \| \| 0.0142 \| \| 0.0017 \| \| 0.0141 \| \| 0.0423 \| \| 0.0166 \| \| 0.0057 \| \| 0.0449 \| \| 0.0162 \| \| 0.0372 \| \| 0.0227 \| \| 0.0234 \| \| 0.0259 \| \| 0.0196 \| \| 0.0017 \| \| 0.0151 \| \| 0.0170 \| \| 0.0465 \| \| 0.0075 \| \| 0.0004 \| \| 0.0015 \| \| 0.0423 \| \| 0.0103 \| \| 0.0216 \| \| 0.0155 \| \| 0.0274 \| \| 0.0092 \| \| 0.0184 \| \| 0.0178 \| \| 0.0183 \| \| 0.0214 \| \| 0.0065 \| \| 0.0228 \| \| 0.0271 \| \| 0.0260 \| \| 0.0156 \| \| 0.0059 \| \| 0.0411 \| \| 0.0477 \| \| 0.0015 \| \| 0.0270 \| \| 0.0155 \| \| 0.0136 \| \| 0.0392 \| \| 0.0431 \| \| 0.0342 \| \| 0.0226 \| \| 0.0181 \| \| 0.0331 \| \| 0.0271 \| \| 0.0214 \| \| 0.0331 \| \| 0.0341 \| \| 0.0205 \| \| 0.0108 \| \| 0.0298 \| \| 0.0366 \| \| 0.0343 \| \| 0.0319 \| \| 0.0060 \| \| 0.0288 \| \| 0.0344 \| \| 0.0024 \| \| 0.0072 \| \| 0.0128 \| \| 0.0274 \| \| 0.0053 \| \| 0.0160 \| \| 0.0368 \| \| 0.0408 \| \| 0.0078 \| \| 0.0359 \| \| 0.0319 \| \| 0.0121 \| \| 0.0142 \| \| 0.0062 \| \| 0.0135 \| \| 0.0262 \| \| 0.0006 \| \| 0.0284 \| \| 0.0250 \| \| 0.0467 \| \| 0.0354 \| \| 0.0046 \| \| 0.0071 \| \| 0.0010 \| \| 0.0059 \| \| 0.0015 \| \| 0.0438 \| \| 0.0297 \| \| 0.0422 \| \| 0.0468 \| \| 0.0466 \| \| 0.0310 \| \| 0.0254 \| \| 0.0101 \| \| 0.0194 \| \| 0.0131 \| \| 0.0200 \| \| 0.0386 \| \| 0.0194 \| \| 0.0231 \| \| 0.0444 \| \| 0.0366 \| \| 0.0019 \| \| 0.0490 \| \| 0.0314 \| \| 0.0449 \| \| 0.0170 \| \| 0.0161 \| \| 0.0024 \| \| 0.0212 \| \| 0.0273 \| \| 0.0131 \| \| 0.0151 \| \| 0.0243 \| \| 0.0319 \| \| 0.0076 \| \| 0.0328 \| \| 0.0230 \| \| 0.0236 \| \| 0.0474 \| \| 0.0048 \| \| 0.0210 \| \| 0.0491 \| \| 0.0448 \| \| 0.0237 \| \| 0.0002 \| \| 0.0127 \| \| 0.0029 \| \| 0.0469 \| \| 0.0113 \| \| 0.0274 \| \| 0.0370 \| \| 0.0438 \| \| 0.0452 \| \| 0.0088 \| \| 0.0282 \| \| 0.0009 \| \| 0.0298 \| \| 0.0202 \| \| 0.0219 \| \| 0.0348 \| \| 0.0411 \| \| 0.0394 \| \| 0.0282 \| \| 0.0105 \| \| 0.0104 \| \| 0.0182 \| \| 0.0237 \| \| 0.0452 \| \| 0.0060 \| \| 0.0202 \| \| 0.0181 \| \| 0.0280 \| \| 0.0230 \| \| 0.0324 \| \| 0.0490 \| \| 0.0051 \| \| 0.0115 \| \| 0.0281 \| \| 0.0461 \| \| 0.0174 \| \| 0.0406 \| \| 0.0405 \| \| 0.0370 \| \| 0.0307 \| \| 0.0393 \| \| 0.0322 \| \| 0.0406 \| \| 0.0170 \| \| 0.0200 \| \| 0.0207 \| \| 0.0077 \| \| 0.0124 \| \| 0.0450 \| \| 0.0413 \| \| 0.0109 \| \| 0.0265 \| \| 0.0490 \| \| 0.0211 \| \| 0.0174 \| \| 0.0051 \| \| 0.0237 \| \| 0.0221 \| \| 0.0065 \| \| 0.0139 \| \| 0.0092 \| \| 0.0339 \| \| 0.0444 \| \| 0.0129 \| \| 0.0019 \| \| 0.0123 \| \| 0.0010 \| \| 0.0459 \| \| 0.0157 \| \| 0.0321 \| \| 0.0067 \| \| 0.0119 \| \| 0.0050 \| \| 0.0191 \| \| 0.0239 \| \| 0.0176 \| \| 0.0291 \| \| 0.0115 \| \| 0.0397 \| \| 0.0164 \| \| 0.0269 \| \| 0.0324 \| \| 0.0018 \| \| 0.0358 \| \| 0.0421 \| \| 0.0464 \| \| 0.0244 \| \| 0.0231 \| \| 0.0498 \| \| 0.0005 \| \| 0.0078 \| \| 0.0449 \| \| 0.0271 \| \| 0.0205 \| \| 0.0415 \| \| 0.0456 \| \| 0.0012 \| \| 0.0152 \| \| 0.0079 \| \| 0.0404 \| \| 0.0243 \| \| 0.0149 \| \| 0.0336 \| \| 0.0406 \| \| 0.0077 \| \| 0.0127 \| \| 0.0203 \| \| 0.0489 \| \| 0.0130 \| \| 0.0324 \| \| 0.0440 \| \| 0.0462 \| \| 0.0335 \| \| 0.0314 \| \| 0.0250 \| \| 0.0213 \| \| 0.0484 \| \| 0.0389 \| \| 0.0352 \| \| 0.0047 \| \| 0.0237 \| \| 0.0164 \| \| 0.0030 \| \| 0.0217 \| \| 0.0397 \| \| 0.0340 \| \| 0.0334 \| \| 0.0264 \| \| 0.0084 \| \| 0.0294 \| \| 0.0120 \| \| 0.0286 \| \| 0.0240 \| \| 0.0416 \| \| 0.0318 \| \| 0.0384 \| \| 0.0348 \| \| 0.0299 \| \| 0.0159 \| \| 0.0327 \| \| 0.0057 \| \| 0.0341 \| \| 0.0241 \| \| 0.0277 \| \| 0.0375 \| \| 0.0304 \| \| 0.0264 \| \| 0.0041 \| \| 0.0349 \| \| 0.0229 \| \| 0.0218 \| \| 0.0259 \| \| 0.0296 \| \| 0.0486 \| \| 0.0404 \| \| 0.0426 \| \| 0.0244 \| \| 0.0117 \| \| 0.0031 \| \| 0.0358 \| \| 0.0385 \| \| 0.0246 \| \| 0.0497 \| \| 0.0499 \| \| 0.0101 \| \| 0.0366 \| \| 0.0379 \| \| 0.0155 \| \| 0.0467 \| \| 0.0359 \| \| 0.0498 \| \| 0.0336 \| \| 0.0173 \| \| 0.0205 \| \| 0.0167 \| \| 0.0200 \| \| 0.0430 \| \| 0.0047 \| \| 0.0113 \| \| 0.0454 \| \| 0.0231 \| \| 0.0322 \| \| 0.0158 \| \| 0.0296 \| \| 0.0289 \| \| 0.0034 \| \| 0.0432 \| \| 0.0270 \| \| 0.0379 \| \| 0.0045 \| \| 0.0125 \| \| 0.0014 \| \| 0.0135 \| \| 0.0360 \| \| 0.0069 \| \| 0.0115 \| \| 0.0414 \| \| 0.0323 \| \| 0.0307 \| \| 0.0248 \| \| 0.0209 \| \| 0.0227 \| \| 0.0195 \| \| 0.0265 \| \| 0.0021 \| \| 0.0374 \| \| 0.0421 \| \| 0.0035 \| \| 0.0187 \| \| 0.0150 \| \| 0.0066 \| \| 0.0313 \| \| 0.0037 \| \| 0.0056 \| \| 0.0182 \| \| 0.0382 \| \| 0.0435 \| \| 0.0066 \| \| 0.0370 \| \| 0.0287 \| \| 0.0221 \| \| 0.0094 \| \| 0.0167 \| \| 0.0415 \| \| 0.0265 \| \| 0.0128 \| \| 0.0440 \| \| 0.0321 \| \| 0.0159 \| \| 0.0483 \| \| 0.0392 \| \| 0.0388 \| \| 0.0278 \| \| 0.0083 \| \| 0.0187 \| \| 0.0405 \| \| 0.0459 \| \| 0.0382 \| \| 0.0226 \| \| 0.0042 \| \| 0.0127 \| \| 0.0483 \| \| 0.0486 \| \| 0.0184 \| \| 0.0288 \| \| 0.0194 \| \| 0.0035 \| \| 0.0327 \| \| 0.0268 \| \| 0.0259 \| \| 0.0264 \| \| 0.0401 \| \| 0.0078 \| \| 0.0347 \| \| 0.0148 \| \| 0.0080 \| \| 0.0417 \| \| 0.0047 \| \| 0.0496 \| \| 0.0088 \| \| 0.0466 \| \| 0.0074 \| \| 0.0225 \| \| 0.0075 \| \| 0.0181 \| \| 0.0357 \| \| 0.0332 \| \| 0.0476 \| \| 0.0163 \| \| 0.0441 \| \| 0.0146 \| \| 0.0337 \| \| 0.0360 \| \| 0.0475 \| \| 0.0291 \| \| 0.0018 \| \| 0.0247 \| \| 0.0188 \| \| 0.0143 \| \| 0.0169 \| \| 0.0457 \| \| 0.0445 \| \| 0.0324 \| \| 0.0222 \| \| 0.0455 \| \| 0.0147 \| \| 0.0499 \| \| 0.0454 \| \| 0.0258 \| \| 0.0189 \| \| 0.0399 \| \| 0.0439 \| \| 0.0127 \| \| 0.0291 \| \| 0.0322 \| \| 0.0075 \| \| 0.0065 \| \| 0.0254 \| \| 0.0493 \| \| 0.0156 \| \| 0.0176 \| \| 0.0386 \| \| 0.0252 \| \| 0.0239 \| \| 0.0095 \| \| 0.0176 \| \| 0.0192 \| \| 0.0315 \| \| 0.0348 \| \| 0.0325 \| \| 0.0307 \| \| 0.0073 \| \| 0.0368 \| \| 0.0331 \| \| 0.0157 \| \| 0.0281 \| \| 0.0288 \| \| 0.0308 \| \| 0.0310 \| \| 0.0082 \| \| 0.0035 \| \| 0.0425 \| \| 0.0455 \| \| 0.0233 \| \| 0.0029 \| \| 0.0293 \| \| 0.0423 \| \| 0.0248 \| \| 0.0278 \| \| 0.0112 \| \| 0.0239 \| \| 0.0239 \| \| 0.0427 \| \| 0.0386 \| \| 0.0400 \| \| 0.0473 \| \| 0.0164 \| \| 0.0171 \| \| 0.0433 \| \| 0.0069 \| \| 0.0201 \| \| 0.0335 \| \| 0.0131 \| \| 0.0147 \| \| 0.0151 \| \| 0.0283 \| \| 0.0434 \| \| 0.0346 \| \| 0.0075 \| \| 0.0365 \| \| 0.0488 \| \| 0.0107 \| \| 0.0359 \| \| 0.0259 \| \| 0.0325 \| \| 0.0426 \| \| 0.0403 \| \| 0.0231 \| \| 0.0325 \| \| 0.0238 \| \| 0.0039 \| \| 0.0304 \| \| 0.0292 \| \| 0.0326 \| \| 0.0461 \| \| 0.0312 \| \| 0.0042 \| \| 0.0440 \| \| 0.0275 \| \| 0.0318 \| \| 0.0101 \| \| 0.0295 \| \| 0.0150 \| \| 0.0373 \| \| 0.0487 \| \| 0.0188 \| \| 0.0475 \| \| 0.0478 \| \| 0.0359 \| \| 0.0446 \| \| 0.0493 \| \| 0.0173 \| \| 0.0471 \| \| 0.0168 \| \| 0.0396 \| \| 0.0353 \| \| 0.0377 \| \| 0.0341 \| \| 0.0374 \| \| 0.0171 \| \| 0.0337 \| \| 0.0407 \| \| 0.0149 \| \| 0.0192 \| \| 0.0053 \| \| 0.0098 \| \| 0.0239 \| \| 0.0215 \| \| 0.0395 \| \| 0.0220 \| \| 0.0363 \| \| 0.0064 \| \| 0.0171 \| \| 0.0357 \| \| 0.0178 \| \| 0.0319 \| \| 0.0110 \| \| 0.0239 \| \| 0.0028 \| \| 0.0264 \| \| 0.0164 \| \| 0.0110 \| \| 0.0437 \| \| 0.0385 \| \| 0.0364 \| \| 0.0319 \| \| 0.0175 \| \| 0.0317 \| \| 0.0400 \| \| 0.0159 \| \| 0.0305 \| \| 0.0421 \| \| 0.0370 \| \| 0.0200 \| \| 0.0262 \| \| 0.0344 \| \| 0.0222 \| \| 0.0471 \| \| 0.0149 \| \| 0.0264 \| \| 0.0399 \| \| 0.0476 \| \| 0.0357 \| \| 0.0199 \| \| 0.0075 \| \| 0.0390 \| \| 0.0355 \| \| 0.0391 \| \| 0.0124 \| \| 0.0336 \| \| 0.0172 \| \| 0.0355 \| \| 0.0378 \| \| 0.0059 \| \| 0.0252 \| \| 0.0232 \| \| 0.0023 \| \| 0.0163 \| \| 0.0284 \| \| 0.0063 \| \| 0.0367 \| \| 0.0415 \| \| 0.0390 \| \| 0.0218 \| \| 0.0427 \| \| 0.0136 \| \| 0.0465 \| \| 0.0053 \| \| 0.0176 \| \| 0.0408 \| \| 0.0467 \| \| 0.0365 \| \| 0.0235 \| \| 0.0418 \| \| 0.0205 \| \| 0.0295 \| \| 0.0488 \| \| 0.0165 \| \| 0.0358 \| \| 0.0384 \| \| 0.0315 \| \| 0.0219 \| \| 0.0414 \| \| 0.0445 \| \| 0.0348 \| \| 0.0277 \| \| 0.0325 \| \| 0.0122 \| \| 0.0282 \| \| 0.0330 \| \| 0.0152 \| \| 0.0365 \| \| 0.0308 \| \| 0.0471 \| \| 0.0152 \| \| 0.0114 \| \| 0.0336 \| \| 0.0185 \| \| 0.0031 \| \| 0.0429 \| \| 0.0308 \| \| 0.0112 \| \| 0.0097 \| \| 0.0263 \| \| 0.0334 \| \| 0.0292 \| \| 0.0461 \| \| 0.0291 \| \| 0.0478 \| \| 0.0093 \| \| 0.0491 \| \| 0.0309 \| \| 0.0137 \| \| 0.0288 \| \| 0.0497 \| \| 0.0370 \| \| 0.0399 \| \| 0.0090 \| \| 0.0213 \| \| 0.0258 \| \| 0.0404 \| \| 0.0357 \| \| 0.0411 \| \| 0.0471 \| \| 0.0436 \| \| 0.0495 \| \| 0.0424 \| \| 0.0096 \| \| 0.0055 \| \| 0.0245 \| \| 0.0448 \| \| 0.0473 \| \| 0.0390 \| \| 0.0168 \| \| 0.0184 \| \| 0.0251 \| \| 0.0251 \| \| 0.0171 \| \| 0.0495 \| \| 0.0193 \| \| 0.0167 \| \| 0.0363 \| \| 0.0137 \| \| 0.0406 \| \| 0.0090 \| \| 0.0322 \| \| 0.0433 \| \| 0.0203 \| \| 0.0163 \| \| 0.0308 \| \| 0.0195 \| \| 0.0249 \| \| 0.0166 \| \| 0.0434 \| \| 0.0448 \| \| 0.0255 \| \| 0.0220 \| \| 0.0253 \| \| 0.0451 \| \| 0.0059 \| \| 0.0057 \| \| 0.0191 \| \| 0.0442 \| \| 0.0486 \| \| 0.0155 \| \| 0.0265 \| \| 0.0439 \| \| 0.0395 \| \| 0.0314 \| \| 0.0156 \| \| 0.0401 \| \| 0.0181 \| \| 0.0460 \| \| 0.0264 \| \| 0.0162 \| \| 0.0390 \| \| 0.0410 \| \| 0.0224 \| \| 0.0447 \| \| 0.0328 \| \| 0.0407 \| \| 0.0463 \| \| 0.0206 \| \| 0.0349 \| \| 0.0254 \| \| 0.0312 \| \| 0.0344 \| \| 0.0430 \| \| 0.0413 \| \| 0.0490 \| \| 0.0436 \| \| 0.0361 \| \| 0.0063 \| \| 0.0243 \| \| 0.0476 \| \| 0.0397 \| \| 0.0338 \| \| 0.0031 \| \| 0.0374 \| \| 0.0351 \| \| 0.0304 \| \| 0.0438 \| \| 0.0491 \| \| 0.0347 \| \| 0.0105 \| \| 0.0319 \| \| 0.0151 \| \| 0.0407 \| \| 0.0290 \| \| 0.0453 \| \| 0.0171 \| \| 0.0360 \| \| 0.0075 \| \| 0.0448 \| \| 0.0370 \| \| 0.0144 \| \| 0.0363 \| \| 0.0117 \| \| 0.0440 \| \| 0.0440 \| \| 0.0243 \| \| 0.0287 \| \| 0.0460 \| \| 0.0298 \| \| 0.0170 \| \| 0.0291 \| \| 0.0351 \| \| 0.0305 \| \| 0.0463 \| \| 0.0461 \| \| 0.0257 \| \| 0.0221 \| \| 0.0432 \| \| 0.0169 \| \| 0.0235 \| \| 0.0278 \| \| 0.0296 \| \| 0.0440 \| \| 0.0294 \| \| 0.0296 \| \| 0.0477 \| \| 0.0270 \| \| 0.0163 \| \| 0.0398 \| \| 0.0434 \| \| 0.0449 \| \| 0.0488 \| \| 0.0349 \| \| 0.0033 \| \| 0.0415 \| \| 0.0092 \| \| 0.0428 \| \| 0.0368 \| \| 0.0385 \| \| 0.0294 \| \| 0.0446 \| \| 0.0409 \| \| 0.0456 \| \| 0.0206 \| \| 0.0475 \| \| 0.0181 \| \| 0.0265 \| \| 0.0353 \| \| 0.0220 \| \| 0.0434 \| \| 0.0459 \| \| 0.0154 \| \| 0.0155 \| \| 0.0376 \| \| 0.0372 \| \| 0.0191 \| \| 0.0062 \| \| 0.0405 \| \| 0.0487 \| \| 0.0154 \| \| 0.0143 \| \| 0.0148 \| \| 0.0461 \| \| 0.0302 \| \| 0.0049 \| \| 0.0384 \| \| 0.0484 \| \| 0.0121 \| \| 0.0495 \| \| 0.0361 \| \| 0.0048 \| \| 0.0367 \| \| 0.0191 \| \| 0.0016 \| \| 0.0496 \| \| 0.0406 \| \| 0.0112 \| \| 0.0481 \| \| 0.0335 \| \| 0.0227 \| \| 0.0162 \| \| 0.0465 \| \| 0.0172 \| \| 0.0107 \| \| 0.0158 \| \| 0.0234 \| \| 0.0442 \| \| 0.0445 \| \| 0.0313 \| \| 0.0307 \| \| 0.0290 \| \| 0.0381 \| \| 0.0446 \| \| 0.0135 \| \| 0.0164 \| \| 0.0302 \| \| 0.0468 \| \| 0.0414 \| \| 0.0387 \| \| 0.0255 \| \| 0.0435 \| \| 0.0435 \| \| 0.0329 \| \| 0.0213 \| \| 0.0255 \| \| 0.0481 \| \| 0.0437 \| \| 0.0470 \| \| 0.0452 \| \| 0.0488 \| \| 0.0171 \| \| 0.0348 \| \| 0.0348 \| \| 0.0404 \| \| 0.0326 \| \| 0.0412 \| \| 0.0271 \| \| 0.0464 \| \| 0.0398 \| \| 0.0240 \| \| 0.0374 \| \| 0.0326 \| \| 0.0461 \| \| 0.0117 \| \| 0.0490 \| \| 0.0326 \| \| 0.0495 \| \| 0.0322 \| \| 0.0421 \| \| 0.0130 \| \| 0.0333 \| \| 0.0447 \| \| 0.0441 \| \| 0.0031 \| \| 0.0473 \| \| 0.0396 \| \| 0.0396 \| \| 0.0377 \| \| 0.0252 \| \| 0.0004 \| \| 0.0151 \| \| 0.0009 \| \| 0.0085 \| \| 0.0492 \| \| 0.0159 \| \| 0.0192 \| \| 0.0269 \| \| 0.0312 \| \| 0.0079 \| \| 0.0311 \| \| 0.0394 \| \| 0.0262 \| \| 0.0251 \| \| 0.0475 \| \| 0.0488 \| \| 0.0097 \| \| 0.0495 \| \| 0.0293 \| \| 0.0131 \| \| 0.0303 \| \| 0.0477 \| \| 0.0041 \| \| 0.0402 \| \| 0.0086 \| \| 0.0307 \| \| 0.0151 \| \| 0.0144 \| \| 0.0434 \| \| 0.0454 \| \| 0.0302 \| \| 0.0288 \| \| 0.0263 \| \| 0.0358 \| \| 0.0495 \| \| 0.0189 \| \| 0.0052 \| \| 0.0285 \| \| 0.0230 \| \| 0.0148 \| \| 0.0066 \| \| 0.0350 \| \| 0.0300 \| \| 0.0251 \| \| 0.0234 \| \| 0.0190 \| \| 0.0428 \| \| 0.0474 \| \| 0.0394 \| \| 0.0232 \| \| 0.0467 \| \| 0.0455 \| \| 0.0233 \| \| 0.0120 \| \| 0.0487 \| \| 0.0384 \| \| 0.0001 \| \| 0.0144 \| \| 0.0490 \| \| 0.0477 \| \| 0.0343 \| \| 0.0310 \| \| 0.0119 \| \| 0.0322 \| \| 0.0396 \| \| 0.0060 \| \| 0.0457 \| \| 0.0220 \| \| 0.0331 \| \| 0.0370 \| \| 0.0079 \| \| 0.0273 \| \| 0.0290 \| \| 0.0378 \| \| 0.0403 \| \| 0.0492 \| \| 0.0159 \| \| 0.0439 \| \| 0.0420 \| \| 0.0487 \| \| 0.0498 \| \| 0.0354 \| \| 0.0411 \| \| 0.0479 \| \| 0.0372 \| \| 0.0428 \| \| 0.0064 \| \| 0.0453 \| \| 0.0308 \| \| 0.0285 \| \| 0.0356 \| \| 0.0007 \| \| 0.0236 \| \| 0.0090 \| \| 0.0391 \| \| 0.0347 \| \| 0.0142 \| \| 0.0260 \| \| 0.0193 \| \| 0.0366 \| \| 0.0328 \| \| 0.0345 \| \| 0.0408 \| \| 0.0217 \| \| 0.0417 \| \| 0.0389 \| \| 0.0182 \| \| 0.0410 \| \| 0.0261 \| \| 0.0354 \| \| 0.0294 \| \| 0.0190 \| \| 0.0311 \| \| 0.0447 \| \| 0.0159 \| \| 0.0290 \| \| 0.0143 \| \| 0.0484 \| \| 0.0281 \| \| 0.0317 \| \| 0.0335 \| \| 0.0367 \| | \| SF3B5 \| \| --- \| \| NSMAF \| \| A830082I02RIK \| \| ICMT \| \| GM566 \| \| TMUB1 \| \| ARV1 \| \| CCDC135 \| \| A330046P14RIK \| \| PEO1 \| \| EG627844 \| \| PPP1R1C \| \| NCDN \| \| RAB7L1 \| \| D830015B12RIK \| \| LOC223645 \| \| JOSD2 \| \| COL9A3 \| \| A230046K03RIK \| \| 5730427N09RIK \| \| D430029G22RIK \| \| BC030396 \| \| NSMCE1 \| \| THOP1 \| \| ATG5 \| \| SOHLH2 \| \| TANK \| \| PSMC3 \| \| 4932441K18RIK \| \| LEPRE1 \| \| IFITM7 \| \| LOC100041864 \| \| B230334I05RIK \| \| TMEM120A \| \| KEAP1 \| \| BC051142 \| \| TATDN1 \| \| ZFP64 \| \| GPHN \| \| SLC35E3 \| \| HNRNPAB \| \| SH2B1 \| \| ZFY1 \| \| ZBTB17 \| \| NCSTN \| \| MPV17L \| \| CHIC1 \| \| 2310007F21RIK \| \| DHDDS \| \| UBE2CBP \| \| 4632412I06RIK \| \| GPS2 \| \| LOC100047674 \| \| NUBP2 \| \| 3110009N10RIK \| \| WDR61 \| \| PML \| \| PPIA \| \| SNAPC4 \| \| LOC675709 \| \| OLFR906 \| \| DDX31 \| \| INVS \| \| SIX1 \| \| SHFM1 \| \| GPER \| \| AKR1C21 \| \| DPM2 \| \| ZRANB2 \| \| HIST1H2AI \| \| HELZ \| \| LOC386514 \| \| SAMD8 \| \| RRAGB \| \| 1700029I15RIK \| \| LOC100048733 \| \| OSBPL8 \| \| 1810007E14RIK \| \| LOC234374 \| \| SLC25A44 \| \| XRCC6BP1 \| \| FXN \| \| 2700097O09RIK \| \| TXNDC15 \| \| NDUFB2 \| \| AW123240 \| \| CTSS \| \| GM1821 \| \| ZFP64 \| \| MYO1B \| \| RPS10 \| \| CYP4F39 \| \| 2310008H09RIK \| \| PSMB1 \| \| 2810410M20RIK \| \| PURB \| \| LOC384985 \| \| APLF \| \| PRDX4 \| \| CSNK1D \| \| BC018507 \| \| LOC100047053 \| \| SMC1A \| \| RDBP \| \| TCF4 \| \| RPP30 \| \| ENPP2 \| \| DUSP13 \| \| SOD1 \| \| 9530014D17RIK \| \| LOC100047091 \| \| BZW1 \| \| HAAO \| \| AI314180 \| \| A930025D01RIK \| \| ATF2 \| \| MFSD10 \| \| MRPL20 \| \| GTF3C1 \| \| GM687 \| \| LGTN \| \| DCLK1 \| \| APPBP1 \| \| BXDC1 \| \| NAPG \| \| KCTD15 \| \| 2410015N17RIK \| \| E130009M23RIK \| \| 4921509B22RIK \| \| 4931436F15RIK \| \| DPH2L1 \| \| B430209F14RIK \| \| VARS2 \| \| 2310040G24RIK \| \| DEXI \| \| B230339M05RIK \| \| SIRT4 \| \| PARD6A \| \| RNPEP \| \| EDG8 \| \| ABCA7 \| \| INVS \| \| RPS21 \| \| HGF \| \| NAPG \| \| NEK3 \| \| C530025M17RIK \| \| KIF13B \| \| CCDC123 \| \| 9530068E07RIK \| \| ZDHHC24 \| \| ORC5L \| \| ACNAT2 \| \| B230339M05RIK \| \| GFM \| \| UXT \| \| MED8 \| \| TMEM53 \| \| TRIB3 \| \| ANGEL1 \| \| WIPI2 \| \| KCTD10 \| \| 9630013D21RIK \| \| SLCO1B2 \| \| LOC233437 \| \| LOC100038949 \| \| CHRNB1 \| \| FAM116B \| \| LDB2 \| \| PSMD4 \| \| MAPKAP1 \| \| MGC18837 \| \| CNOT3 \| \| KRTAP16-1 \| \| MORN3 \| \| SCL0003799.1_2 \| \| PNKP \| \| RDBP \| \| SMARCE1 \| \| LHX4 \| \| SCOTIN \| \| LOC242025 \| \| TRIM11 \| \| CYLD \| \| SIGLECH \| \| ZFP820 \| \| LOC223827 \| \| RNF31 \| \| NAT6 \| \| PTRF \| \| CLK2 \| \| PHOX2A \| \| SLAIN2 \| \| PEX16 \| \| OLFR1029 \| \| CTSZ \| \| PAFAH2 \| \| LOC100047393 \| \| 2610101N10RIK \| \| 7530408C15RIK \| \| CRIP2 \| \| NRAP \| \| EG240327 \| \| FOXA3 \| \| BC049762 \| \| D12ERTD647E \| \| 1600002K03RIK \| \| 9130221H12RIK \| \| 1810048J11RIK \| \| GRTP1 \| \| ZFP292 \| \| RABIF \| \| SRPRB \| \| HNRPM \| \| LOC677205 \| \| MTVR2 \| \| IGFBP2 \| \| AIPL1 \| \| A530075A22RIK \| \| AA407659 \| \| OLFR1502 \| \| CDK7 \| \| 1810041L15RIK \| \| 4930563E18RIK \| \| 1700013G24RIK \| \| UBTF \| \| SLC2A2 \| \| PPP4C \| \| TUBA1A \| \| A430106B04RIK \| \| MAPKAPK3 \| \| 4930589O11RIK \| \| TRPV2 \| \| OLFR1186 \| \| GPRC5B \| \| REM1 \| \| CNIH4 \| \| GM459 \| \| PRKCZ \| \| CHRNB4 \| \| ZFP637 \| \| CTNNBL1 \| \| KIF7 \| \| LOC383955 \| \| SCL0001849.1_223 \| \| SIDT2 \| \| SLC10A3 \| \| CAPN10 \| \| SERPINA1A \| \| DCUN1D4 \| \| TMEM87A \| \| ORMDL3 \| \| C130074O09RIK \| \| LOC668164 \| \| 3110023E09RIK \| \| ISY1 \| \| TMEM5 \| \| A430060D24RIK \| \| SLC7A10 \| \| ACADL \| \| POP1 \| \| RDBP \| \| D130064A21RIK \| \| CHMP2A \| \| CCR10 \| \| PHF20L1 \| \| UBE2W \| \| 1810026B05RIK \| \| 4930488I03RIK \| \| CHMP2A \| \| BC003331 \| \| LSM1 \| \| FBP2 \| \| GDPD1 \| \| ARF2 \| \| ZFP213 \| \| CECR2 \| \| FBXL18 \| \| FXC1 \| \| ADAM28 \| \| AIFM2 \| \| SMARCAL1 \| \| PSMB2 \| \| LIN28B \| \| YEATS2 \| \| EIF4A2 \| \| HOXB5 \| \| PCSK4 \| \| TNFSF13B \| \| 4930471M23RIK \| \| LOC100045981 \| \| XPR1 \| \| 2610528M18RIK \| \| ABCA3 \| \| PBRM1 \| \| CAMK2N2 \| \| REPS1 \| \| KLRA7 \| \| NOS3 \| \| MAPKAP1 \| \| 5730409G15RIK \| \| NUDCD2 \| \| LOC330240 \| \| 1110039B18RIK \| \| TYK2 \| \| MPHOSPH6 \| \| EG667410 \| \| 2410018G20RIK \| \| RNF113A1 \| \| DDI2 \| \| TBX4 \| \| LOC218889 \| \| 4930526H21RIK \| \| 4930455J15RIK \| \| PCDHGA4 \| \| SLC35A5 \| \| SLMAP \| \| C330046G03RIK \| \| IRGM1 \| \| ZFP791 \| \| USP50 \| \| ARHGEF1 \| \| RFFL \| \| LOC383741 \| \| TMEM127 \| \| TSPAN15 \| \| 2610524H06RIK \| \| NDFIP2 \| \| 2810025M15RIK \| \| UHRF1BP1 \| \| 9430015L11RIK \| \| 5730559C18RIK \| \| 4930563P21RIK \| \| GNAQ \| \| LOC232745 \| \| COTL1 \| \| TRAPPC4 \| \| DTX4 \| \| NES \| \| RGS7 \| \| RGS12 \| \| FZD8 \| \| UBE3A \| \| PSMD12 \| \| UBOX5 \| \| FAM113B \| \| GOLGA3 \| \| HSD3B2 \| \| 5830467E07RIK \| \| ZFP277 \| \| 1700060J05RIK \| \| 2410124H12RIK \| \| 1200015F23RIK \| \| NOL6 \| \| PSIP1 \| \| D130007C19RIK \| \| TRAM1 \| \| CHIA \| \| 4932438A13RIK \| \| LOC100047579 \| \| NCF4 \| \| LBCL1 \| \| EARS2 \| \| DEFB9 \| \| LOC664956 \| \| FAIM \| \| LOC381174 \| \| 2010204N08RIK \| \| PPP1R3E \| \| TRAPPC4 \| \| CSGALNACT1 \| \| ICK \| \| NCDN \| \| COASY \| \| A230077I10RIK \| \| TAPT1 \| \| MFAP3 \| \| GJA7 \| \| LOC100044779 \| \| LOC381185 \| \| 2410019G02RIK \| \| PNPLA2 \| \| KBTBD10 \| \| 5730449L18RIK \| \| FAM169B \| \| D14ERTD668E \| \| 2700062C07RIK \| \| D730045A05RIK \| \| PJA1 \| \| DNMT1 \| \| GPATCH4 \| \| RPGRIP1 \| \| D11WSU99E \| \| RNPEP \| \| LOC434019 \| \| HIST1H2AE \| \| SMARCA3 \| \| TBX10 \| \| CNIH4 \| \| MRPS26 \| \| D11WSU99E \| \| B930097L24RIK \| \| ENTPD7 \| \| CNTN2 \| \| PRUNE \| \| PPIL2 \| \| A430059D01RIK \| \| PRB1 \| \| BICD2 \| \| LOC384410 \| \| ADAMTS1 \| \| FBXO21 \| \| 9530058B02RIK \| \| MCAT \| \| PSMC3IP \| \| TERF2 \| \| PFKL \| \| RANBP1 \| \| 9630024J24RIK \| \| ACOT4 \| \| SETX \| \| PMM1 \| \| STAT2 \| \| AMIGO2 \| \| 4921506I22RIK \| \| LOC236831 \| \| MCM5 \| \| GATA6 \| \| WHDC1 \| \| LOC100039751 \| \| RAD23B \| \| MSCP-PENDING \| \| BC004728 \| \| NKX2-1 \| \| 6430550D23RIK \| \| 1700038P13RIK \| \| PPP2R2B \| \| 4931429L15RIK \| \| SMN1 \| \| MS4A2 \| \| FBXO45 \| \| 1110036O03RIK \| \| 2510009E07RIK \| \| D330018I10RIK \| \| LOC380884 \| \| LOC272693 \| \| GM1527 \| \| LOC384130 \| \| SLC9A7 \| \| TMEM51 \| \| EG432825 \| \| MALT-1 \| \| CD63 \| \| F730014I05RIK \| \| 6820408C15RIK \| \| BCDIN3D \| \| H2-T23 \| \| C230042N14RIK \| \| IPO4 \| \| LOC100047915 \| \| SCGB2B1 \| \| 9130014M22RIK \| \| BEND5 \| \| A930033C23RIK \| \| LOC385679 \| \| LOC384417 \| \| CYP2A4 \| \| GNGT2 \| \| METTL1 \| \| 4930568H22RIK \| \| PCBD2 \| \| TAX1BP3 \| \| UBE1X \| \| OLFR1140 \| \| C130018J17RIK \| \| CUL1 \| \| WDR45 \| \| EXOC3L \| \| TPR \| \| DTX2 \| \| RPL29 \| \| RSPO1 \| \| SLC35B4 \| \| C230077B03RIK \| \| HIST1H3F \| \| NDOR1 \| \| 2010110I21RIK \| \| NUDT5 \| \| CYP3A57 \| \| PLEC1 \| \| D430039N05RIK \| \| PEX6 \| \| CDK4 \| \| C630030D09RIK \| \| TRFP \| \| HECTD3 \| \| MBTPS2 \| \| LOC100041500 \| \| RRAS \| \| RAVER1 \| \| ZBP1 \| \| CHCHD4 \| \| SYTL3 \| \| V1RC16 \| \| MED31 \| \| 7330434K15RIK \| \| 4930405K06RIK \| \| ABHD4 \| \| SLC19A1 \| \| HDAC10 \| \| NAGK \| \| RNF40 \| \| 4933407H18RIK \| \| PSMA5 \| \| PSMG3 \| \| LOC635470 \| \| SH3BP5L \| \| ATG16L2 \| \| GALNT10 \| \| D830044I01RIK \| \| TMEM144 \| \| SGCG \| \| UNC119B \| \| SEL1L \| \| BCL2L2 \| \| CDH20 \| \| MMP1A \| \| 4933414I15RIK \| \| D430031C12RIK \| \| DYSF \| \| GNL3 \| \| LAS1L \| \| ZSWIM1 \| \| SAP130 \| \| A430107D22RIK \| \| WARS2 \| \| LOC100047888 \| \| RPL19 \| \| CLDN15 \| \| LOC219049 \| \| ANXA4 \| \| 9330154K18RIK \| \| PSMB10 \| \| HIST2H3C1 \| \| A930004K21RIK \| \| DHX37 \| \| MAGOH \| \| 1110049B09RIK \| \| TIGD3 \| \| PPAP2A \| \| ATP2B4 \| \| ARF6 \| \| HOXB9 \| \| HEY2 \| \| KCTD12 \| \| ACTR1B \| \| NDOR1 \| \| ALDH5A1 \| \| 1700025E21RIK \| \| LYSMD1 \| \| HIST2H3B \| \| TMEM66 \| \| TMEM107 \| \| MAGMAS \| \| PRPF19 \| \| 2310046O06RIK \| \| SSH3 \| \| TJP2 \| \| ZDHHC13 \| \| LTBR \| \| DIAP3 \| \| CLCA5 \| \| LOC384593 \| \| ARHGEF1 \| \| SSU72 \| \| 4930556L07RIK \| \| YY2 \| \| ARMC5 \| \| SCOTIN \| \| APBB1IP \| \| MLLT3 \| \| CPB2 \| \| PFKL \| \| UBFD1 \| \| GOLPH3L \| \| CHAT \| \| SLC9A8 \| \| PLA2G3 \| \| 2310009E04RIK \| \| NIN \| \| RRP1B \| \| HEG1 \| \| SLITRK5 \| \| LOC385634 \| \| ACCN3 \| \| DPH3 \| \| 7330410H16RIK \| \| THADA \| \| WIPI1 \| \| FGA \| \| ING4 \| \| ELP2 \| \| GTPBP3 \| \| LOC386054 \| \| QTRT1 \| \| SSBP3 \| \| JAM2 \| \| LOC380906 \| \| BRAF \| \| PARP2 \| \| SUMF2 \| \| DTWD1 \| \| 2810003K23RIK \| \| LOC236223 \| \| LOC385267 \| \| LOC100044185 \| \| PLA2G6 \| \| SLC22A12 \| \| EIF3S10 \| \| V1RA3 \| \| PHLDB1 \| \| LOC382883 \| \| PLAGL1 \| \| SETD1B \| \| CTPS \| \| NEK3 \| \| RXFP2 \| \| RNF182 \| \| TPRKB \| \| NMD3 \| \| RFC1 \| \| GHITM \| \| C130066B05RIK \| \| ENO2 \| \| JARID2 \| \| EGF \| \| CHCHD8 \| \| A530050N04RIK \| \| POLR3B \| \| ARNT \| \| LOC100046800 \| \| PRUNE \| \| EG627311 \| \| SLC25A24 \| \| LOC380947 \| \| CNN2 \| \| CTNNBIP1 \| \| MKIAA0250 \| \| SLC2A9 \| \| ISYNA1 \| \| TAX1BP1 \| \| 9630013D22RIK \| \| PSMD11 \| \| LOC100047184 \| \| BMP4 \| \| 4930535I16RIK \| \| LOC386027 \| \| LOC100045947 \| \| LOC383819 \| \| GLG1 \| \| TSEN54 \| \| 1700016L04RIK \| \| LOC668160 \| \| MGLL \| \| PSMD11 \| \| OLFR1423 \| \| LOC383428 \| \| SGSM3 \| \| 6330408J11RIK \| \| A730059B08RIK \| \| MYO18B \| \| 0610039K10RIK \| \| 0610009B22RIK \| \| 9627020_131 \| \| LOC381215 \| \| COX19 \| \| PSMC4 \| \| D2BWG1423E \| \| OTTMUSG010136 \| \| P2RX5 \| \| PRMT5 \| \| LOC215538 \| \| SLC29A3 \| \| EG384187 \| \| FAM18A \| \| ELAC1 \| \| LOC386364 \| \| LOC243833 \| \| ZFPM2 \| \| AZIN1 \| \| COG1 \| \| SPAG16 \| \| MYBBP1A \| \| EIF3S1 \| \| NOLA1 \| \| CAPN2 \| \| OLFR1113 \| \| DNCIC2 \| \| SEMA3F \| \| STAT6 \| \| SRD5A3 \| \| MTA3 \| \| ZDHHC14 \| \| 1700041G16RIK \| \| HEATR1 \| \| DLX4 \| \| 1110008F13RIK \| \| 2810012D02RIK \| \| NRARP \| \| EXOC1 \| \| SSR2 \| \| RILPL2 \| \| 2310065K24RIK \| \| A730036E13RIK \| \| KIF13A \| \| UCK1 \| \| SLC16A13 \| \| MVD \| \| RDH5 \| \| UNC84A \| \| 1700023L04RIK \| \| LOC236356 \| \| 4930511N13RIK \| \| ASL \| \| LOC100048504 \| \| MYEF2 \| \| A630020I15RIK \| \| LOC230592 \| \| SIRPB1 \| \| GMFG \| \| CNOT6L \| \| PRPF19 \| \| LOC381365 \| \| SMPD4 \| \| PNRC2 \| \| H2-T23 \| \| SOX13 \| \| HSPA4L \| \| 9130221F21 \| \| UBQLN3 \| \| TNFSF13B \| \| BTRC \| \| RREB1 \| \| A530065E19RIK \| \| GMFG \| \| INSL5 \| \| NOS1AP \| \| OLFR320 \| \| MRPL51 \| \| GRAMD2 \| \| LOC641178 \| \| PPP1R7 \| \| TRPM5 \| \| PKMYT1 \| \| KTI12 \| \| PCDHB4 \| \| CCDC126 \| \| BC004728 \| \| 1700128E19RIK \| \| ZDHHC12 \| \| CYLD \| \| KIF1B \| \| VARS \| \| LRRC59 \| \| MGC118210 \| \| CDC42EP2 \| \| TFB1M \| \| TRIM6 \| \| STK33 \| \| E130308J18RIK \| \| NEF3 \| \| E130112N23RIK \| \| SLC16A9 \| \| BOP1 \| \| A830080D01RIK \| \| GNPTAB \| \| LOC193403 \| \| OLFR76 \| \| GPSM1 \| \| AW555464 \| \| HSPB3 \| \| F830001A22RIK \| \| CD302 \| \| PLEKHM3 \| \| RFX1 \| \| ZFP524 \| \| HABP2 \| \| CHMP5 \| \| G3BP1 \| \| 9626123_206 \| \| PLDN \| \| NAGK \| \| 4921521F21RIK \| \| YWHAZ \| \| LOC383376 \| \| CHERP \| \| AHSG \| \| 6030400N17RIK \| \| FARP2 \| \| LOC384829 \| \| COX4I2 \| \| PAK4 \| \| NTHL1 \| \| IL16 \| \| A230050P20RIK \| \| EG330070 \| \| OLFR1066 \| \| LYSMD2 \| \| PIGF \| \| LOC384601 \| \| EG547109 \| \| 9830143E02RIK \| \| TSEN15 \| \| RBL1 \| \| LYSMD1 \| \| TNC \| \| 2410091C18RIK \| \| ARID3B \| \| D630024O11RIK \| \| PHKB \| \| 5330411L03RIK \| \| SH3BGRL3 \| \| NOC2L \| \| VPS37C \| \| EG270499 \| \| LOC675899 \| \| TJAP1 \| \| 9030411M15RIK \| \| LOC269134 \| \| MAP3K14 \| \| F830005D05RIK \| \| LOC382908 \| \| TMEM202 \| \| L3MBTL \| \| SMC5L1 \| \| LOC545369 \| \| MEIS1 \| \| LOC216605 \| \| DYRK1A \| \| DNHD1 \| \| E330023P07RIK \| \| MRPS35 \| \| AA409316 \| \| LOC100048508 \| \| 4921530F17RIK \| \| LOC671641 \| \| B230312L03RIK \| \| GGT1 \| \| CSMD3 \| \| TCF12 \| \| GNA11 \| \| DMRTC1A \| \| NFIX \| \| LOC100045484 \| \| EPB4.1L4A \| \| SCL0003499.1_101 \| \| RREB1 \| \| 4831426I19RIK \| \| LOC381925 \| \| LOC245475 \| \| 5730589K01RIK \| \| 1700012H05RIK \| \| LOC627424 \| \| PSMB5 \| \| ANAPC1 \| \| 7530416G11RIK \| \| LOC193533 \| \| D630047N04RIK \| \| COL11A2 \| \| XRCC1 \| \| BC024479 \| \| TSPAN4 \| \| STFA2 \| \| WDR78 \| \| PLSCR4 \| \| D9ERTD392E \| \| CREBL1 \| \| GALNT2 \| \| FBXW11 \| \| ULK3 \| \| ZFP41 \| \| LOC277278 \| \| LOC238662 \| \| 1700010M22RIK \| \| OLFR1508 \| \| TRMT1 \| \| STARD3NL \| \| EPX \| \| RNF113A2 \| \| BC057170 \| \| UPF1 \| \| POMT2 \| \| 8430438D04RIK \| \| FBXO2 \| \| 5031409G22RIK \| \| LOC100047749 \| \| DIP2A \| \| 3110057O12RIK \| \| KIF18A \| \| 2900045G02RIK \| \| 1600012H06RIK \| \| MRPL49 \| \| TAAR7D \| \| FIP1L1 \| \| PAQR9 \| \| 2310005G13RIK \| \| 8030481M12RIK \| \| LRCH1 \| \| D030050C19RIK \| \| AHCY \| \| AV249152 \| \| DPH1 \| \| IFITM1 \| \| TREX1 \| \| E030034P13RIK \| \| KRTAP5-2 \| \| B130024G19RIK \| \| PPP1R1A \| \| LOC654494 \| \| LOC385791 \| \| EPHA7 \| \| 3010026O09RIK \| \| 6030458C11RIK \| \| MJ-8000-191_7593 \| \| SNAPC3 \| \| LOC245015 \| \| MALL \| \| LOC276837 \| \| PHF13 \| \| OAS2 \| \| 9030619P08RIK \| \| ARV1 \| \| UNC93B1 \| \| EIF2B4 \| \| 2810429I04RIK \| \| 4833409A17RIK \| \| 5730419I09RIK \| \| RBPJ \| \| CDC40 \| \| PLEKHM1 \| \| LOC100042540 \| \| C130071E11RIK \| \| TDPOZ1 \| \| RBM13 \| \| KLHL15 \| \| 9830141J12RIK \| \| SEMA3G \| \| 4733401I05RIK \| \| D630041L13RIK \| \| 2410131K14RIK \| \| BLM \| \| IGHV1S136_IG_154 \| \| ZWINT \| \| MTFMT \| \| SEC22A \| \| LOC381386 \| \| LOC239516 \| \| E130009J12RIK \| \| TAL1 \| \| 9430095K15RIK \| \| IGFBP3 \| \| LOC381792 \| \| ZFP365 \| \| VPS33B \| \| CHD4 \| \| BXDC5 \| \| FBLIM1 \| \| UTY \| \| LOC384659 \| \| CEP164 \| \| PFDN4 \| \| ANKK1 \| \| GIMAP5 \| \| LOC626583 \| \| ARMC10 \| \| KLF17 \| \| GEMIN4 \| \| DSE \| \| 6720458F09RIK \| \| DYSF \| \| TMEM33 \| \| BBS10 \| \| OLFR747 \| \| A030005L19RIK \| \| ROR1 \| \| IFT80 \| \| TTC30B \| \| ITGA4 \| \| GABRD \| \| UNC13A \| \| RAD23B \| \| PPP1R1B \| \| GCS1 \| \| AKIRIN1 \| \| ZFYVE1 \| \| FANCE \| \| D930048N14RIK \| \| KNS2 \| \| POLR1C \| \| 0610005I04 \| \| OLFR635 \| \| A730049B06RIK \| \| HYAL6 \| \| PIGC \| \| 2010312A17RIK \| \| STK32C \| \| ITGA7 \| \| LOC100040016 \| \| RHOD \| \| LOC100039649 \| \| CNPY2 \| \| D430002C13RIK \| \| SPN \| \| UBE2U \| \| 2900016J09RIK \| \| OLFR1462 \| \| OLFR1033 \| \| WDR6 \| \| 5031434O11RIK \| \| DEFB7 \| \| OTTMUSG0005523 \| \| LOC236435 \| \| SAMD11 \| \| A730023M06RIK \| \| E2F5 \| \| STAT1 \| \| ACTR3 \| \| 2410018G20RIK \| \| NR2C2AP \| \| TIMM9 \| \| PHLDB1 \| \| TUBA8 \| \| NCLN \| \| EG333830 \| \| ARID1A \| \| CALN1 \| \| ANAPC7 \| \| OLFR706 \| \| SLCO4A1 \| \| LOC382362 \| \| LOC381208 \| \| CIC \| \| 4921537P18RIK \| \| PSG28 \| \| FEZ1 \| \| IRGQ \| \| EG383815 \| \| D10627 \| \| ST7L \| \| ARFIP2 \| \| LTV1 \| \| INTS4 \| \| LOC383027 \| \| A930019E22RIK \| \| DPM3 \| \| MCM9 \| \| CDSN \| \| LOC385982 \| \| NFIC \| \| KLC1 \| \| CADPS2 \| \| H2-Q5 \| \| ARHGAP4 \| \| ACOT10 \| \| LOC381668 \| \| LGALS7 \| \| OLFR1442 \| \| MGLL \| \| PLEC1 \| \| CCNJ \| \| 1190009E20RIK \| \| 1810010M01RIK \| \| PML \| \| LOC381387 \| \| TUBA8 \| \| CORO2B \| \| 1300003B13RIK \| \| FLNB \| \| NGB \| \| CAV1 \| \| 5730449L18RIK \| \| GRASP \| \| MOCOS \| \| LOC100047273 \| \| GLTSCR2 \| \| MLLT1 \| \| LOC329416 \| \| ZFYVE28 \| \| PPP4R1 \| \| MSX2 \| \| ENAH \| \| 9930016I07RIK \| \| C130074G19RIK \| \| RRP12 \| \| BC037034 \| \| UGDH \| \| 4931429I11RIK \| \| HOXB7 \| \| GZMD \| \| DOCK10 \| \| CTSM \| \| FAF1 \| \| ETNK2 \| \| 9630033F20RIK \| \| MAPKAPK3 \| \| D330028D13RIK \| \| NHLRC1 \| \| TTYH2 \| \| OLFR1176 \| \| 4833444L21RIK \| \| LOC383491 \| \| 9630020E24RIK \| \| MSH4 \| \| USP20 \| \| SIAH1B \| \| POLR1E \| \| IFITM1 \| \| IFIT2 \| \| FBXW16 \| \| TMBIM1 \| \| B530045E10RIK \| \| AGTR2 \| \| SENP5 \| \| 4933400C05RIK \| \| 4930430K04RIK \| \| TSPAN18 \| \| D6ERTD474E \| \| ALS2 \| \| TIMM10 \| \| SPNB1 \| \| NRN1 \| \| CD93 \| \| LHX6 \| \| GALR3 \| \| 5930400O15RIK \| \| IGHV1S28_IG_13 \| \| 2810441K11RIK \| \| OLFML2A \| \| SMARCD1 \| \| RIBC1 \| \| LOC380924 \| \| OLFR1256 \| \| GM962 \| \| IL20RB \| \| UBE4B \| \| TMEM66 \| \| ALS2CR12 \| \| SAMD9L \| \| LOC380706 \| \| 5330403J18RIK \| \| 4930433I11RIK \| \| KLHL15 \| \| CTSZ \| \| ADCY4 \| \| 2900064A13RIK \| \| NAPEPLD \| \| 5330420J21RIK \| \| CDH26 \| \| LOC381273 \| \| C330014O21RIK \| \| 1700019E19RIK \| \| ZFP82 \| \| PPAP2A \| \| FBXO10 \| \| RPS6KB1 \| \| OFCC1 \| \| SLC6A20B \| \| SLC12A1 \| \| HIBCH \| \| PLCL2 \| \| TRIM23 \| \| OLFR628 \| \| A330017A19RIK \| \| LOC380929 \| \| SLC16A12 \| \| ABCA17 \| \| SSTR2 \| \| IDS \| \| C330049P11RIK \| \| SLC16A8 \| \| PCDHA5 \| \| LOC381002 \| \| D430015D21RIK \| \| 6330503K22RIK \| \| DDX20 \| \| IL28RA \| \| RIPK4 \| \| A830086L01RIK \| \| PSMC2 \| \| 1700009P17RIK \| \| CPSF2 \| \| HEBP2 \| \| 4831437C03RIK \| \| EGFR \| \| GNA11 \| \| PYGL \| \| TLN2 \| \| TNRC18 \| \| ORF9 \| \| SPRED3 \| \| CBX6 \| \| AZI1 \| \| CD68 \| \| METTL8 \| \| NUMB \| \| CCDC9 \| \| OMT2B \| \| CYP26A1 \| \| ANKRD45 \| \| VAV3 \| \| KCTD17 \| \| AI317223 \| \| 2010001E11RIK \| \| TMEM53 \| \| SLAM \| \| WIZ \| \| LOC239770 \| \| SCL0002023.1_27 \| \| NPBWR1 \| \| TK1 \| \| TRAT1 \| \| MYO18B \| \| LOC223672 \| \| 5330404D22RIK \| \| DDX27 \| \| LOC385249 \| \| AI661453 \| \| PSRC1 \| \| 2310051N18RIK \| \| LMX1A \| \| SLC6A9 \| \| EDNRA \| \| RIN1 \| \| TROVE2 \| \| CAT \| \| TCIRG1 \| \| ADRM1 \| \| LOC630729 \| \| GM484 \| \| A030003K21RIK \| \| LY6E \| \| SNX24 \| \| SSXB2 \| \| 5830418G11RIK \| \| MOSPD4 \| \| PHIP \| \| RSC1A1 \| \| GM62 \| \| DPH4 \| \| PAPSS2 \| \| MARCKSL1 \| \| OLFR114 \| \| 1110007L15RIK \| \| NMB \| \| GM347 \| \| 4930418I18RIK \| \| CREM \| \| 2610018G03RIK \| \| C330023M02RIK \| \| PRMT6 \| \| PRHOXNB \| \| MKS1 \| \| NOL14 \| \| E2F4 \| \| CD1D2 \| \| E130311K13RIK \| \| HBB-Y \| \| LOC100047606 \| \| MSH6 \| \| MTMR11 \| \| 4930455F16RIK \| \| LOC381190 \| \| ADCY4 \| \| PRL2A1 \| \| GFRA3 \| \| IL17F \| \| AQP7 \| \| PLVAP \| \| ALG6 \| \| 1810058I24RIK \| \| IFT81 \| \| PROSC \| \| C130046B21RIK \| \| LOC245538 \| \| B430205M18RIK \| \| DENND1B \| \| TJAP1 \| \| PDGFB \| \| LOC333637 \| \| LY96 \| \| ZFP169 \| \| COG8 \| \| UBE2Q2 \| \| AHNAK \| \| OLFR670 \| \| CPNE2 \| \| WDR77 \| \| APBB1 \| \| RNF213 \| \| MMP15 \| \| COL11A1 \| \| GRIK1 \| \| CPSF4L \| \| PSMD1 \| \| PANX1 \| \| BC053749 \| \| LOC381888 \| \| LOC271788 \| \| OLFR453 \| \| 4933403G14RIK \| \| NFKBIE \| \| EPHB4 \| \| GM1077 \| \| FNBP1 \| \| ZFP617 \| \| RANBP6 \| \| MMRN2 \| \| 4831428F09RIK \| \| PRRX1 \| \| SIGLEC1 \| \| NIT1 \| \| IL1A \| \| D630040I23RIK \| \| OLFR1454 \| \| LOC381277 \| \| 1500005K14RIK \| \| VSIG2 \| \| LOC381557 \| \| DACH1 \| \| OLFR16 \| \| 9330177P20RIK \| \| 1700129L04RIK \| \| LIPE \| \| TUBB2C \| \| ADAM8 \| \| 4833413D08RIK \| \| AI413582 \| \| ENPP3 \| \| MAP3K5 \| \| LY6E \| \| HIST1H2BN \| \| AB112350 \| \| RFX1 \| \| GREB1 \| \| NUMB \| \| OLFR154 \| \| SRI \| \| CAR5B \| \| GGA2 \| \| CHRNA9 \| \| L3MBTL2 \| \| ACTR1B \| \| PALM \| \| 6230412A12RIK \| \| NUDT11 \| \| DSG3 \| \| LOC638050 \| \| TXN1 \| \| TAAR6 \| \| SH3RF2 \| \| CORO1B \| \| MMP10 \| \| TRIB2 \| \| GGA2 \| \| JTV1 \| \| HIST1H2BJ \| \| MEST \| \| DMC1 \| \| LOC627424 \| \| LGALS12 \| \| HIST1H2BE \| \| PKMYT1 \| \| SLC27A5 \| \| TEC \| \| 2610035D17RIK \| \| TMEM46 \| \| 2810409M01RIK \| \| ZFP607 \| \| TAPBPL \| \| DSCR3 \| \| 6330562C20RIK \| \| AY761185 \| \| LOC665281 \| \| NUDT14 \| \| IL1RAP \| \| TYRP1 \| \| DDX21 \| \| SEL1L2 \| \| P2RX1 \| \| HIST1H2AC \| \| LOC239502 \| \| DUX \| \| DYSFIP1 \| \| OLFR781 \| \| TTC24 \| \| LASS1 \| \| 2900073G15RIK \| \| CDKAL1 \| \| OLFR1317 \| \| EHD4 \| \| EAR12 \| \| CITED2 \| \| GPR12 \| \| LOC385009 \| \| CARD14 \| \| SLC12A3 \| \| LOC383025 \| \| HIST1H2BH \| \| DBPHT2 \| \| LRRC2 \| \| ABCD4 \| \| APOC1 \| \| NECAB1 \| \| EPHA5 \| \| IL1RAP \| \| C730023J07RIK \| \| LOC226972 \| \| RNF6 \| \| 2010111I01RIK \| \| OLFR1255 \| \| DNASE1L3 \| \| SMR1 \| \| ZFP364 \| \| A230083H22RIK \| \| 2410091N08RIK \| \| PDLIM3 \| \| HOOK2 \| \| EG434758 \| \| SV2B \| \| NAIP2 \| \| PSMD8 \| \| TNFSF14 \| \| PSMD12 \| \| PRLPL \| \| LOC237769 \| \| PCDHA2 \| \| MYO18A \| \| TEX12 \| \| MBC2 \| \| CSAD \| \| LOC100048537 \| \| DPYSL2 \| \| PADI6 \| \| THAP7 \| \| A930010I20RIK \| \| OTUB2 \| \| ABO \| \| LOC100047126 \| \| JTV1 \| \| TNFRSF19 \| \| RAD52 \| \| INMT \| \| USH1C \| \| LOC244417 \| \| EVI2A \| \| HIST1H2BM \| \| CCND1 \| \| DHRS9 \| \| NPLOC4 \| \| CXCR7 \| \| D830014E11RIK \| \| ITGB1BP3 \| \| AI661453 \| \| PDLIM3 \| \| ALDH3A1 \| \| EG627636 \| \| ASXL3 \| \| ST6GALNAC4 \| \| MKNK2 \| \| GPIHBP1 \| \| ABHD2 \| \| 2310014F06RIK \| \| LOC382531 \| \| HGS \| \| MICAL3 \| \| INMT \| \| PPP1R14D \| \| INSL3 \| \| OTUB2 \| \| FGF3 \| \| ZC3H6 \| \| SEMA6A \| \| GSTA3 \| \| 5033425G24RIK \| \| STX6 \| \| D930046M07RIK \| \| LOC100045625 \| \| BC043934 \| \| 8430438E03RIK \| \| ST3GAL4 \| \| HIST1H2BE \| \| SH3RF2 \| \| LRBA \| \| LST1 \| \| MBOAT1 \| \| NCAN \| \| SHISA2 \| \| 9930004G02RIK \| \| SPAG5 \| \| H1F0 \| \| CNGA3 \| \| LOC385977 \| \| ANKRD37 \| \| HIST1H2BC \| \| TTLL10 \| \| ASPM \| \| LOC100038882 \| \| IFIT3 \| \| OASL2 \| \| 2310043N10RIK \| \| HIST1H2BK \| \| B930041G04 \| \| TAT \| \| ASPH \| \| HIST1H2BC \| \| SPSB2 \| \| ST6GALNAC4 \| \| UBE1L \| \| BEX4 \| \| DDIT4L \| \| WDR45L \| \| NOL3 \| \| ACOT1 \| \| DDC \| \| HIST1H1C \| \| RBP7 \| \| PANX1 \| \| HIST1H1C \| \| C920004C08RIK \| \| 1190002H23RIK \| \| HSPE1 \| \| FLNC \| \| 9330132O05RIK \| \| FBXL13 \| \| LOC638892 \| \| LOC100046120 \| \| HSPB7 \| \| ADORA1 \| \| MUSTN1 \| \| MUSTN1 \| | \| -1.1 \| \| --- \| \| -1.1 \| \| -1.1 \| \| -1.1 \| \| -1.1 \| \| -1.1 \| \| -1.2 \| \| -1.2 \| \| -1.2 \| \| -1.2 \| \| -1.2 \| \| -1.2 \| \| -1.2 \| \| -1.2 \| \| -1.2 \| \| -1.2 \| \| -1.2 \| \| -1.2 \| \| -1.2 \| \| -1.2 \| \| -1.2 \| \| -1.2 \| \| -1.2 \| \| -1.2 \| \| -1.2 \| \| -1.2 \| \| -1.2 \| \| -1.2 \| \| -1.2 \| \| -1.2 \| \| -1.2 \| \| -1.2 \| \| -1.2 \| \| -1.2 \| \| -1.2 \| \| -1.2 \| \| -1.2 \| \| -1.2 \| \| -1.2 \| \| -1.2 \| \| -1.2 \| \| -1.2 \| \| -1.2 \| \| -1.2 \| \| -1.2 \| \| -1.2 \| \| -1.2 \| \| -1.2 \| \| -1.2 \| \| -1.2 \| \| -1.2 \| \| -1.2 \| \| -1.2 \| \| -1.2 \| \| -1.2 \| \| -1.2 \| \| -1.2 \| \| -1.2 \| \| -1.2 \| \| -1.2 \| \| -1.2 \| \| -1.2 \| \| -1.2 \| \| -1.2 \| \| -1.2 \| \| -1.2 \| \| -1.2 \| \| -1.2 \| \| -1.2 \| \| -1.2 \| \| -1.2 \| \| -1.2 \| \| -1.2 \| \| -1.2 \| \| -1.2 \| \| -1.2 \| \| -1.2 \| \| -1.2 \| \| -1.2 \| \| -1.2 \| \| -1.2 \| \| -1.2 \| \| -1.2 \| \| -1.2 \| \| -1.2 \| \| -1.2 \| \| -1.2 \| \| -1.2 \| \| -1.2 \| \| -1.2 \| \| -1.2 \| \| -1.2 \| \| -1.2 \| \| -1.2 \| \| -1.2 \| \| -1.2 \| \| -1.2 \| \| -1.2 \| \| -1.2 \| \| -1.2 \| \| -1.2 \| \| -1.2 \| \| -1.2 \| \| -1.2 \| \| -1.2 \| \| -1.2 \| \| -1.2 \| \| -1.2 \| \| -1.2 \| \| -1.2 \| \| -1.2 \| \| -1.2 \| \| -1.2 \| \| -1.2 \| \| -1.2 \| \| -1.2 \| \| -1.2 \| \| -1.2 \| \| -1.2 \| \| -1.2 \| \| -1.2 \| \| -1.2 \| \| -1.2 \| \| -1.2 \| \| -1.2 \| \| -1.2 \| \| -1.2 \| \| -1.2 \| \| -1.2 \| \| -1.2 \| \| -1.2 \| \| -1.2 \| \| -1.2 \| \| -1.2 \| \| -1.2 \| \| -1.2 \| \| -1.2 \| \| -1.2 \| \| -1.2 \| \| -1.2 \| \| -1.2 \| \| -1.2 \| \| -1.2 \| \| -1.2 \| \| -1.2 \| \| -1.2 \| \| -1.2 \| \| -1.2 \| \| -1.2 \| \| -1.2 \| \| -1.2 \| \| -1.2 \| \| -1.2 \| \| -1.2 \| \| -1.2 \| \| -1.2 \| \| -1.2 \| \| -1.2 \| \| -1.2 \| \| -1.2 \| \| -1.2 \| \| -1.2 \| \| -1.2 \| \| -1.2 \| \| -1.2 \| \| -1.2 \| \| -1.2 \| \| -1.2 \| \| -1.2 \| \| -1.2 \| \| -1.2 \| \| -1.2 \| \| -1.2 \| \| -1.2 \| \| -1.2 \| \| -1.2 \| \| -1.2 \| \| -1.2 \| \| -1.2 \| \| -1.2 \| \| -1.2 \| \| -1.2 \| \| -1.2 \| \| -1.2 \| \| -1.2 \| \| -1.2 \| \| -1.2 \| \| -1.2 \| \| -1.2 \| \| -1.2 \| \| -1.2 \| \| -1.2 \| \| -1.2 \| \| -1.2 \| \| -1.2 \| \| -1.2 \| \| -1.2 \| \| -1.2 \| \| -1.2 \| \| -1.2 \| \| -1.2 \| \| -1.2 \| \| -1.2 \| \| -1.2 \| \| -1.2 \| \| -1.2 \| \| -1.2 \| \| -1.2 \| \| -1.2 \| \| -1.2 \| \| -1.2 \| \| -1.2 \| \| -1.2 \| \| -1.2 \| \| -1.2 \| \| -1.2 \| \| -1.2 \| \| -1.2 \| \| -1.2 \| \| -1.2 \| \| -1.2 \| \| -1.2 \| \| -1.2 \| \| -1.2 \| \| -1.2 \| \| -1.2 \| \| -1.2 \| \| -1.2 \| \| -1.2 \| \| -1.2 \| \| -1.2 \| \| -1.2 \| \| -1.2 \| \| -1.2 \| \| -1.2 \| \| -1.2 \| \| -1.2 \| \| -1.2 \| \| -1.2 \| \| -1.2 \| \| -1.2 \| \| -1.2 \| \| -1.2 \| \| -1.2 \| \| -1.2 \| \| -1.2 \| \| -1.2 \| \| -1.2 \| \| -1.2 \| \| -1.2 \| \| -1.2 \| \| -1.2 \| \| -1.2 \| \| -1.2 \| \| -1.2 \| \| -1.2 \| \| -1.2 \| \| -1.2 \| \| -1.2 \| \| -1.2 \| \| -1.2 \| \| -1.2 \| \| -1.2 \| \| -1.2 \| \| -1.2 \| \| -1.2 \| \| -1.2 \| \| -1.2 \| \| -1.2 \| \| -1.2 \| \| -1.2 \| \| -1.2 \| \| -1.2 \| \| -1.2 \| \| -1.2 \| \| -1.2 \| \| -1.2 \| \| -1.2 \| \| -1.2 \| \| -1.2 \| \| -1.2 \| \| -1.2 \| \| -1.2 \| \| -1.2 \| \| -1.2 \| \| -1.2 \| \| -1.2 \| \| -1.2 \| \| -1.2 \| \| -1.2 \| \| -1.2 \| \| -1.2 \| \| -1.2 \| \| -1.2 \| \| -1.2 \| \| -1.2 \| \| -1.2 \| \| -1.2 \| \| -1.2 \| \| -1.2 \| \| -1.2 \| \| -1.2 \| \| -1.2 \| \| -1.2 \| \| -1.2 \| \| -1.2 \| \| -1.2 \| \| -1.2 \| \| -1.2 \| \| -1.2 \| \| -1.2 \| \| -1.2 \| \| -1.2 \| \| -1.2 \| \| -1.2 \| \| -1.2 \| \| -1.2 \| \| -1.2 \| \| -1.2 \| \| -1.2 \| \| -1.2 \| \| -1.2 \| \| -1.2 \| \| -1.2 \| \| -1.2 \| \| -1.2 \| \| -1.2 \| \| -1.2 \| \| -1.2 \| \| -1.2 \| \| -1.2 \| \| -1.2 \| \| -1.2 \| \| -1.2 \| \| -1.2 \| \| -1.2 \| \| -1.2 \| \| -1.2 \| \| -1.2 \| \| -1.2 \| \| -1.2 \| \| -1.2 \| \| -1.2 \| \| -1.2 \| \| -1.2 \| \| -1.2 \| \| -1.2 \| \| -1.2 \| \| -1.2 \| \| -1.2 \| \| -1.2 \| \| -1.2 \| \| -1.2 \| \| -1.2 \| \| -1.2 \| \| -1.2 \| \| -1.2 \| \| -1.2 \| \| -1.2 \| \| -1.2 \| \| -1.2 \| \| -1.2 \| \| -1.2 \| \| -1.2 \| \| -1.2 \| \| -1.2 \| \| -1.2 \| \| -1.2 \| \| -1.2 \| \| -1.2 \| \| -1.2 \| \| -1.2 \| \| -1.2 \| \| -1.2 \| \| -1.2 \| \| -1.2 \| \| -1.2 \| \| -1.2 \| \| -1.2 \| \| -1.2 \| \| -1.2 \| \| -1.2 \| \| -1.2 \| \| -1.2 \| \| -1.2 \| \| -1.2 \| \| -1.2 \| \| -1.2 \| \| -1.2 \| \| -1.2 \| \| -1.2 \| \| -1.2 \| \| -1.2 \| \| -1.2 \| \| -1.2 \| \| -1.2 \| \| -1.2 \| \| -1.2 \| \| -1.2 \| \| -1.2 \| \| -1.2 \| \| -1.2 \| \| -1.2 \| \| -1.2 \| \| -1.2 \| \| -1.2 \| \| -1.2 \| \| -1.2 \| \| -1.2 \| \| -1.2 \| \| -1.2 \| \| -1.2 \| \| -1.2 \| \| -1.2 \| \| -1.2 \| \| -1.2 \| \| -1.2 \| \| -1.2 \| \| -1.2 \| \| -1.2 \| \| -1.2 \| \| -1.2 \| \| -1.2 \| \| -1.2 \| \| -1.2 \| \| -1.2 \| \| -1.2 \| \| -1.2 \| \| -1.2 \| \| -1.2 \| \| -1.2 \| \| -1.2 \| \| -1.2 \| \| -1.2 \| \| -1.2 \| \| -1.2 \| \| -1.2 \| \| -1.2 \| \| -1.2 \| \| -1.2 \| \| -1.2 \| \| -1.2 \| \| -1.2 \| \| -1.2 \| \| -1.2 \| \| -1.2 \| \| -1.2 \| \| -1.2 \| \| -1.2 \| \| -1.2 \| \| -1.2 \| \| -1.2 \| \| -1.2 \| \| -1.2 \| \| -1.2 \| \| -1.2 \| \| -1.2 \| \| -1.2 \| \| -1.2 \| \| -1.2 \| \| -1.2 \| \| -1.2 \| \| -1.2 \| \| -1.2 \| \| -1.2 \| \| -1.2 \| \| -1.2 \| \| -1.2 \| \| -1.2 \| \| -1.2 \| \| -1.2 \| \| -1.2 \| \| -1.2 \| \| -1.2 \| \| -1.2 \| \| -1.2 \| \| -1.2 \| \| -1.2 \| \| -1.2 \| \| -1.2 \| \| -1.2 \| \| -1.2 \| \| -1.2 \| \| -1.2 \| \| -1.2 \| \| -1.2 \| \| -1.2 \| \| -1.2 \| \| -1.2 \| \| -1.2 \| \| -1.2 \| \| -1.2 \| \| -1.2 \| \| -1.2 \| \| -1.2 \| \| -1.2 \| \| -1.2 \| \| -1.2 \| \| -1.2 \| \| -1.2 \| \| -1.2 \| \| -1.2 \| \| -1.2 \| \| -1.2 \| \| -1.2 \| \| -1.2 \| \| -1.2 \| \| -1.2 \| \| -1.2 \| \| -1.2 \| \| -1.2 \| \| -1.2 \| \| -1.2 \| \| -1.2 \| \| -1.2 \| \| -1.2 \| \| -1.2 \| \| -1.2 \| \| -1.2 \| \| -1.2 \| \| -1.2 \| \| -1.2 \| \| -1.2 \| \| -1.2 \| \| -1.2 \| \| -1.2 \| \| -1.2 \| \| -1.2 \| \| -1.2 \| \| -1.2 \| \| -1.2 \| \| -1.2 \| \| -1.2 \| \| -1.2 \| \| -1.2 \| \| -1.2 \| \| -1.2 \| \| -1.2 \| \| -1.2 \| \| -1.2 \| \| -1.2 \| \| -1.2 \| \| -1.2 \| \| -1.2 \| \| -1.2 \| \| -1.2 \| \| -1.2 \| \| -1.2 \| \| -1.2 \| \| -1.2 \| \| -1.2 \| \| -1.2 \| \| -1.2 \| \| -1.2 \| \| -1.2 \| \| -1.2 \| \| -1.2 \| \| -1.2 \| \| -1.2 \| \| -1.2 \| \| -1.2 \| \| -1.2 \| \| -1.2 \| \| -1.2 \| \| -1.2 \| \| -1.2 \| \| -1.2 \| \| -1.2 \| \| -1.2 \| \| -1.2 \| \| -1.2 \| \| -1.2 \| \| -1.2 \| \| -1.2 \| \| -1.2 \| \| -1.2 \| \| -1.2 \| \| -1.2 \| \| -1.2 \| \| -1.2 \| \| -1.2 \| \| -1.2 \| \| -1.2 \| \| -1.2 \| \| -1.2 \| \| -1.2 \| \| -1.2 \| \| -1.2 \| \| -1.2 \| \| -1.2 \| \| -1.2 \| \| -1.2 \| \| -1.2 \| \| -1.2 \| \| -1.2 \| \| -1.2 \| \| -1.2 \| \| -1.2 \| \| -1.2 \| \| -1.2 \| \| -1.2 \| \| -1.2 \| \| -1.2 \| \| -1.2 \| \| -1.2 \| \| -1.2 \| \| -1.2 \| \| -1.2 \| \| -1.2 \| \| -1.2 \| \| -1.2 \| \| -1.2 \| \| -1.2 \| \| -1.2 \| \| -1.2 \| \| -1.2 \| \| -1.2 \| \| -1.2 \| \| -1.2 \| \| -1.2 \| \| -1.2 \| \| -1.2 \| \| -1.2 \| \| -1.2 \| \| -1.2 \| \| -1.2 \| \| -1.2 \| \| -1.2 \| \| -1.2 \| \| -1.2 \| \| -1.2 \| \| -1.2 \| \| -1.2 \| \| -1.2 \| \| -1.2 \| \| -1.2 \| \| -1.2 \| \| -1.2 \| \| -1.2 \| \| -1.2 \| \| -1.2 \| \| -1.2 \| \| -1.2 \| \| -1.2 \| \| -1.2 \| \| -1.2 \| \| -1.2 \| \| -1.2 \| \| -1.2 \| \| -1.2 \| \| -1.2 \| \| -1.2 \| \| -1.2 \| \| -1.2 \| \| -1.2 \| \| -1.2 \| \| -1.2 \| \| -1.2 \| \| -1.2 \| \| -1.2 \| \| -1.2 \| \| -1.2 \| \| -1.2 \| \| -1.2 \| \| -1.2 \| \| -1.2 \| \| -1.2 \| \| -1.2 \| \| -1.2 \| \| -1.2 \| \| -1.2 \| \| -1.2 \| \| -1.2 \| \| -1.2 \| \| -1.2 \| \| -1.2 \| \| -1.2 \| \| -1.2 \| \| -1.2 \| \| -1.2 \| \| -1.2 \| \| -1.2 \| \| -1.2 \| \| -1.2 \| \| -1.2 \| \| -1.2 \| \| -1.2 \| \| -1.2 \| \| -1.2 \| \| -1.2 \| \| -1.2 \| \| -1.2 \| \| -1.2 \| \| -1.2 \| \| -1.2 \| \| -1.2 \| \| -1.2 \| \| -1.2 \| \| -1.2 \| \| -1.2 \| \| -1.2 \| \| -1.2 \| \| -1.2 \| \| -1.2 \| \| -1.2 \| \| -1.2 \| \| -1.2 \| \| -1.2 \| \| -1.2 \| \| -1.2 \| \| -1.2 \| \| -1.2 \| \| -1.2 \| \| -1.2 \| \| -1.2 \| \| -1.2 \| \| -1.2 \| \| -1.2 \| \| -1.2 \| \| -1.2 \| \| -1.2 \| \| -1.2 \| \| -1.2 \| \| -1.2 \| \| -1.2 \| \| -1.2 \| \| -1.2 \| \| -1.2 \| \| -1.2 \| \| -1.2 \| \| -1.2 \| \| -1.2 \| \| -1.2 \| \| -1.2 \| \| -1.2 \| \| -1.2 \| \| -1.2 \| \| -1.2 \| \| -1.2 \| \| -1.2 \| \| -1.2 \| \| -1.2 \| \| -1.2 \| \| -1.2 \| \| -1.2 \| \| -1.2 \| \| -1.2 \| \| -1.2 \| \| -1.2 \| \| -1.2 \| \| -1.2 \| \| -1.2 \| \| -1.2 \| \| -1.2 \| \| -1.2 \| \| -1.2 \| \| -1.2 \| \| -1.2 \| \| -1.2 \| \| -1.2 \| \| -1.2 \| \| -1.2 \| \| -1.2 \| \| -1.2 \| \| -1.2 \| \| -1.2 \| \| -1.2 \| \| -1.2 \| \| -1.2 \| \| -1.2 \| \| -1.2 \| \| -1.2 \| \| -1.2 \| \| -1.3 \| \| -1.3 \| \| -1.3 \| \| -1.3 \| \| -1.3 \| \| -1.3 \| \| -1.3 \| \| -1.3 \| \| -1.3 \| \| -1.3 \| \| -1.3 \| \| -1.3 \| \| -1.3 \| \| -1.3 \| \| -1.3 \| \| -1.3 \| \| -1.3 \| \| -1.3 \| \| -1.3 \| \| -1.3 \| \| -1.3 \| \| -1.3 \| \| -1.3 \| \| -1.3 \| \| -1.3 \| \| -1.3 \| \| -1.3 \| \| -1.3 \| \| -1.3 \| \| -1.3 \| \| -1.3 \| \| -1.3 \| \| -1.3 \| \| -1.3 \| \| -1.3 \| \| -1.3 \| \| -1.3 \| \| -1.3 \| \| -1.3 \| \| -1.3 \| \| -1.3 \| \| -1.3 \| \| -1.3 \| \| -1.3 \| \| -1.3 \| \| -1.3 \| \| -1.3 \| \| -1.3 \| \| -1.3 \| \| -1.3 \| \| -1.3 \| \| -1.3 \| \| -1.3 \| \| -1.3 \| \| -1.3 \| \| -1.3 \| \| -1.3 \| \| -1.3 \| \| -1.3 \| \| -1.3 \| \| -1.3 \| \| -1.3 \| \| -1.3 \| \| -1.3 \| \| -1.3 \| \| -1.3 \| \| -1.3 \| \| -1.3 \| \| -1.3 \| \| -1.3 \| \| -1.3 \| \| -1.3 \| \| -1.3 \| \| -1.3 \| \| -1.3 \| \| -1.3 \| \| -1.3 \| \| -1.3 \| \| -1.3 \| \| -1.3 \| \| -1.3 \| \| -1.3 \| \| -1.3 \| \| -1.3 \| \| -1.3 \| \| -1.3 \| \| -1.3 \| \| -1.3 \| \| -1.3 \| \| -1.3 \| \| -1.3 \| \| -1.3 \| \| -1.3 \| \| -1.3 \| \| -1.3 \| \| -1.3 \| \| -1.3 \| \| -1.3 \| \| -1.3 \| \| -1.3 \| \| -1.3 \| \| -1.3 \| \| -1.3 \| \| -1.3 \| \| -1.3 \| \| -1.3 \| \| -1.3 \| \| -1.3 \| \| -1.3 \| \| -1.3 \| \| -1.3 \| \| -1.3 \| \| -1.3 \| \| -1.3 \| \| -1.3 \| \| -1.3 \| \| -1.3 \| \| -1.3 \| \| -1.3 \| \| -1.3 \| \| -1.3 \| \| -1.3 \| \| -1.3 \| \| -1.3 \| \| -1.3 \| \| -1.3 \| \| -1.3 \| \| -1.3 \| \| -1.3 \| \| -1.3 \| \| -1.3 \| \| -1.3 \| \| -1.3 \| \| -1.3 \| \| -1.3 \| \| -1.3 \| \| -1.3 \| \| -1.3 \| \| -1.3 \| \| -1.3 \| \| -1.3 \| \| -1.3 \| \| -1.3 \| \| -1.3 \| \| -1.3 \| \| -1.3 \| \| -1.3 \| \| -1.3 \| \| -1.3 \| \| -1.3 \| \| -1.3 \| \| -1.3 \| \| -1.3 \| \| -1.3 \| \| -1.3 \| \| -1.3 \| \| -1.3 \| \| -1.3 \| \| -1.3 \| \| -1.3 \| \| -1.3 \| \| -1.3 \| \| -1.3 \| \| -1.3 \| \| -1.3 \| \| -1.3 \| \| -1.3 \| \| -1.3 \| \| -1.3 \| \| -1.3 \| \| -1.3 \| \| -1.3 \| \| -1.3 \| \| -1.3 \| \| -1.3 \| \| -1.3 \| \| -1.3 \| \| -1.3 \| \| -1.3 \| \| -1.3 \| \| -1.3 \| \| -1.3 \| \| -1.3 \| \| -1.3 \| \| -1.3 \| \| -1.3 \| \| -1.3 \| \| -1.3 \| \| -1.3 \| \| -1.3 \| \| -1.3 \| \| -1.3 \| \| -1.3 \| \| -1.3 \| \| -1.3 \| \| -1.3 \| \| -1.3 \| \| -1.3 \| \| -1.3 \| \| -1.3 \| \| -1.3 \| \| -1.3 \| \| -1.3 \| \| -1.3 \| \| -1.3 \| \| -1.3 \| \| -1.3 \| \| -1.3 \| \| -1.3 \| \| -1.3 \| \| -1.3 \| \| -1.3 \| \| -1.3 \| \| -1.3 \| \| -1.3 \| \| -1.3 \| \| -1.3 \| \| -1.3 \| \| -1.3 \| \| -1.3 \| \| -1.3 \| \| -1.3 \| \| -1.3 \| \| -1.3 \| \| -1.3 \| \| -1.3 \| \| -1.3 \| \| -1.3 \| \| -1.3 \| \| -1.3 \| \| -1.3 \| \| -1.3 \| \| -1.3 \| \| -1.3 \| \| -1.3 \| \| -1.3 \| \| -1.3 \| \| -1.3 \| \| -1.3 \| \| -1.3 \| \| -1.3 \| \| -1.3 \| \| -1.3 \| \| -1.3 \| \| -1.3 \| \| -1.3 \| \| -1.3 \| \| -1.3 \| \| -1.3 \| \| -1.3 \| \| -1.3 \| \| -1.3 \| \| -1.3 \| \| -1.3 \| \| -1.3 \| \| -1.3 \| \| -1.3 \| \| -1.3 \| \| -1.3 \| \| -1.3 \| \| -1.3 \| \| -1.3 \| \| -1.3 \| \| -1.3 \| \| -1.3 \| \| -1.3 \| \| -1.3 \| \| -1.3 \| \| -1.3 \| \| -1.3 \| \| -1.3 \| \| -1.3 \| \| -1.3 \| \| -1.3 \| \| -1.3 \| \| -1.3 \| \| -1.3 \| \| -1.3 \| \| -1.3 \| \| -1.3 \| \| -1.3 \| \| -1.3 \| \| -1.3 \| \| -1.3 \| \| -1.3 \| \| -1.3 \| \| -1.3 \| \| -1.3 \| \| -1.3 \| \| -1.3 \| \| -1.3 \| \| -1.3 \| \| -1.3 \| \| -1.3 \| \| -1.3 \| \| -1.3 \| \| -1.3 \| \| -1.3 \| \| -1.3 \| \| -1.3 \| \| -1.3 \| \| -1.3 \| \| -1.3 \| \| -1.3 \| \| -1.3 \| \| -1.3 \| \| -1.3 \| \| -1.3 \| \| -1.3 \| \| -1.3 \| \| -1.3 \| \| -1.3 \| \| -1.3 \| \| -1.3 \| \| -1.3 \| \| -1.3 \| \| -1.3 \| \| -1.3 \| \| -1.3 \| \| -1.3 \| \| -1.3 \| \| -1.3 \| \| -1.3 \| \| -1.3 \| \| -1.3 \| \| -1.3 \| \| -1.3 \| \| -1.3 \| \| -1.3 \| \| -1.3 \| \| -1.3 \| \| -1.3 \| \| -1.3 \| \| -1.3 \| \| -1.3 \| \| -1.3 \| \| -1.3 \| \| -1.3 \| \| -1.3 \| \| -1.3 \| \| -1.3 \| \| -1.3 \| \| -1.3 \| \| -1.3 \| \| -1.3 \| \| -1.3 \| \| -1.3 \| \| -1.3 \| \| -1.3 \| \| -1.3 \| \| -1.3 \| \| -1.3 \| \| -1.3 \| \| -1.3 \| \| -1.3 \| \| -1.3 \| \| -1.3 \| \| -1.3 \| \| -1.3 \| \| -1.3 \| \| -1.3 \| \| -1.3 \| \| -1.3 \| \| -1.3 \| \| -1.3 \| \| -1.3 \| \| -1.3 \| \| -1.3 \| \| -1.3 \| \| -1.3 \| \| -1.3 \| \| -1.3 \| \| -1.3 \| \| -1.3 \| \| -1.3 \| \| -1.3 \| \| -1.3 \| \| -1.3 \| \| -1.3 \| \| -1.3 \| \| -1.3 \| \| -1.3 \| \| -1.3 \| \| -1.3 \| \| -1.3 \| \| -1.3 \| \| -1.3 \| \| -1.3 \| \| -1.3 \| \| -1.3 \| \| -1.3 \| \| -1.3 \| \| -1.3 \| \| -1.3 \| \| -1.3 \| \| -1.3 \| \| -1.3 \| \| -1.3 \| \| -1.3 \| \| -1.3 \| \| -1.3 \| \| -1.3 \| \| -1.3 \| \| -1.3 \| \| -1.3 \| \| -1.3 \| \| -1.3 \| \| -1.3 \| \| -1.3 \| \| -1.3 \| \| -1.3 \| \| -1.3 \| \| -1.3 \| \| -1.3 \| \| -1.3 \| \| -1.3 \| \| -1.3 \| \| -1.3 \| \| -1.3 \| \| -1.3 \| \| -1.3 \| \| -1.3 \| \| -1.3 \| \| -1.3 \| \| -1.3 \| \| -1.3 \| \| -1.3 \| \| -1.3 \| \| -1.3 \| \| -1.3 \| \| -1.3 \| \| -1.3 \| \| -1.3 \| \| -1.3 \| \| -1.3 \| \| -1.3 \| \| -1.3 \| \| -1.3 \| \| -1.3 \| \| -1.3 \| \| -1.3 \| \| -1.3 \| \| -1.3 \| \| -1.3 \| \| -1.3 \| \| -1.3 \| \| -1.3 \| \| -1.3 \| \| -1.3 \| \| -1.3 \| \| -1.3 \| \| -1.3 \| \| -1.3 \| \| -1.3 \| \| -1.3 \| \| -1.3 \| \| -1.3 \| \| -1.3 \| \| -1.3 \| \| -1.3 \| \| -1.3 \| \| -1.3 \| \| -1.3 \| \| -1.3 \| \| -1.3 \| \| -1.3 \| \| -1.3 \| \| -1.3 \| \| -1.3 \| \| -1.3 \| \| -1.3 \| \| -1.3 \| \| -1.3 \| \| -1.4 \| \| -1.4 \| \| -1.4 \| \| -1.4 \| \| -1.4 \| \| -1.4 \| \| -1.4 \| \| -1.4 \| \| -1.4 \| \| -1.4 \| \| -1.4 \| \| -1.4 \| \| -1.4 \| \| -1.4 \| \| -1.4 \| \| -1.4 \| \| -1.4 \| \| -1.4 \| \| -1.4 \| \| -1.4 \| \| -1.4 \| \| -1.4 \| \| -1.4 \| \| -1.4 \| \| -1.4 \| \| -1.4 \| \| -1.4 \| \| -1.4 \| \| -1.4 \| \| -1.4 \| \| -1.4 \| \| -1.4 \| \| -1.4 \| \| -1.4 \| \| -1.4 \| \| -1.4 \| \| -1.4 \| \| -1.4 \| \| -1.4 \| \| -1.4 \| \| -1.4 \| \| -1.4 \| \| -1.4 \| \| -1.4 \| \| -1.4 \| \| -1.4 \| \| -1.4 \| \| -1.4 \| \| -1.4 \| \| -1.4 \| \| -1.4 \| \| -1.4 \| \| -1.4 \| \| -1.4 \| \| -1.4 \| \| -1.4 \| \| -1.4 \| \| -1.4 \| \| -1.4 \| \| -1.4 \| \| -1.4 \| \| -1.4 \| \| -1.4 \| \| -1.4 \| \| -1.4 \| \| -1.4 \| \| -1.4 \| \| -1.4 \| \| -1.4 \| \| -1.4 \| \| -1.4 \| \| -1.4 \| \| -1.4 \| \| -1.4 \| \| -1.4 \| \| -1.4 \| \| -1.4 \| \| -1.4 \| \| -1.4 \| \| -1.4 \| \| -1.4 \| \| -1.4 \| \| -1.4 \| \| -1.4 \| \| -1.4 \| \| -1.4 \| \| -1.4 \| \| -1.4 \| \| -1.4 \| \| -1.4 \| \| -1.4 \| \| -1.4 \| \| -1.4 \| \| -1.4 \| \| -1.4 \| \| -1.4 \| \| -1.4 \| \| -1.4 \| \| -1.4 \| \| -1.4 \| \| -1.4 \| \| -1.4 \| \| -1.4 \| \| -1.4 \| \| -1.4 \| \| -1.4 \| \| -1.4 \| \| -1.4 \| \| -1.4 \| \| -1.4 \| \| -1.4 \| \| -1.4 \| \| -1.4 \| \| -1.4 \| \| -1.4 \| \| -1.4 \| \| -1.4 \| \| -1.4 \| \| -1.4 \| \| -1.4 \| \| -1.4 \| \| -1.4 \| \| -1.4 \| \| -1.4 \| \| -1.4 \| \| -1.4 \| \| -1.4 \| \| -1.4 \| \| -1.4 \| \| -1.4 \| \| -1.4 \| \| -1.4 \| \| -1.4 \| \| -1.4 \| \| -1.4 \| \| -1.4 \| \| -1.4 \| \| -1.4 \| \| -1.4 \| \| -1.4 \| \| -1.4 \| \| -1.4 \| \| -1.4 \| \| -1.4 \| \| -1.4 \| \| -1.4 \| \| -1.4 \| \| -1.4 \| \| -1.4 \| \| -1.4 \| \| -1.4 \| \| -1.4 \| \| -1.4 \| \| -1.4 \| \| -1.4 \| \| -1.4 \| \| -1.4 \| \| -1.4 \| \| -1.4 \| \| -1.4 \| \| -1.4 \| \| -1.4 \| \| -1.4 \| \| -1.4 \| \| -1.4 \| \| -1.4 \| \| -1.4 \| \| -1.4 \| \| -1.4 \| \| -1.4 \| \| -1.4 \| \| -1.4 \| \| -1.4 \| \| -1.4 \| \| -1.4 \| \| -1.4 \| \| -1.4 \| \| -1.4 \| \| -1.4 \| \| -1.4 \| \| -1.4 \| \| -1.4 \| \| -1.4 \| \| -1.4 \| \| -1.4 \| \| -1.4 \| \| -1.4 \| \| -1.4 \| \| -1.4 \| \| -1.4 \| \| -1.4 \| \| -1.4 \| \| -1.5 \| \| -1.5 \| \| -1.5 \| \| -1.5 \| \| -1.5 \| \| -1.5 \| \| -1.5 \| \| -1.5 \| \| -1.5 \| \| -1.5 \| \| -1.5 \| \| -1.5 \| \| -1.5 \| \| -1.5 \| \| -1.5 \| \| -1.5 \| \| -1.5 \| \| -1.5 \| \| -1.5 \| \| -1.5 \| \| -1.5 \| \| -1.5 \| \| -1.5 \| \| -1.5 \| \| -1.5 \| \| -1.5 \| \| -1.5 \| \| -1.5 \| \| -1.5 \| \| -1.5 \| \| -1.5 \| \| -1.5 \| \| -1.5 \| \| -1.5 \| \| -1.5 \| \| -1.5 \| \| -1.5 \| \| -1.5 \| \| -1.5 \| \| -1.5 \| \| -1.5 \| \| -1.5 \| \| -1.5 \| \| -1.5 \| \| -1.5 \| \| -1.5 \| \| -1.5 \| \| -1.5 \| \| -1.5 \| \| -1.5 \| \| -1.5 \| \| -1.5 \| \| -1.5 \| \| -1.5 \| \| -1.5 \| \| -1.5 \| \| -1.5 \| \| -1.5 \| \| -1.5 \| \| -1.5 \| \| -1.6 \| \| -1.6 \| \| -1.6 \| \| -1.6 \| \| -1.6 \| \| -1.6 \| \| -1.6 \| \| -1.6 \| \| -1.6 \| \| -1.6 \| \| -1.6 \| \| -1.6 \| \| -1.6 \| \| -1.6 \| \| -1.6 \| \| -1.6 \| \| -1.6 \| \| -1.6 \| \| -1.6 \| \| -1.7 \| \| -1.7 \| \| -1.7 \| \| -1.7 \| \| -1.7 \| \| -1.7 \| \| -1.8 \| \| -1.8 \| \| -1.8 \| \| -1.8 \| \| -1.8 \| \| -1.9 \| \| -2.0 \| \| -2.0 \| \| -2.1 \| \| -2.1 \| \| -2.2 \| \| -2.2 \| \| -2.6 \| \| -2.8 \| \| -3.4 \| \| -3.7 \| \| -3.7 \| | \| 0.0101 \| \| --- \| \| 0.0441 \| \| 0.0364 \| \| 0.0085 \| \| 0.0162 \| \| 0.0479 \| \| 0.0167 \| \| 0.0183 \| \| 0.0376 \| \| 0.0313 \| \| 0.0161 \| \| 0.0211 \| \| 0.0422 \| \| 0.0239 \| \| 0.0473 \| \| 0.0402 \| \| 0.0413 \| \| 0.0347 \| \| 0.0390 \| \| 0.0165 \| \| 0.0080 \| \| 0.0473 \| \| 0.0192 \| \| 0.0100 \| \| 0.0481 \| \| 0.0465 \| \| 0.0426 \| \| 0.0129 \| \| 0.0497 \| \| 0.0138 \| \| 0.0118 \| \| 0.0361 \| \| 0.0460 \| \| 0.0273 \| \| 0.0496 \| \| 0.0490 \| \| 0.0398 \| \| 0.0181 \| \| 0.0279 \| \| 0.0316 \| \| 0.0229 \| \| 0.0437 \| \| 0.0491 \| \| 0.0411 \| \| 0.0448 \| \| 0.0453 \| \| 0.0352 \| \| 0.0495 \| \| 0.0479 \| \| 0.0305 \| \| 0.0298 \| \| 0.0343 \| \| 0.0409 \| \| 0.0473 \| \| 0.0314 \| \| 0.0474 \| \| 0.0457 \| \| 0.0389 \| \| 0.0335 \| \| 0.0361 \| \| 0.0331 \| \| 0.0482 \| \| 0.0497 \| \| 0.0492 \| \| 0.0490 \| \| 0.0212 \| \| 0.0042 \| \| 0.0074 \| \| 0.0499 \| \| 0.0453 \| \| 0.0198 \| \| 0.0377 \| \| 0.0219 \| \| 0.0241 \| \| 0.0370 \| \| 0.0443 \| \| 0.0274 \| \| 0.0484 \| \| 0.0309 \| \| 0.0288 \| \| 0.0336 \| \| 0.0159 \| \| 0.0155 \| \| 0.0231 \| \| 0.0430 \| \| 0.0365 \| \| 0.0359 \| \| 0.0419 \| \| 0.0261 \| \| 0.0406 \| \| 0.0118 \| \| 0.0391 \| \| 0.0419 \| \| 0.0071 \| \| 0.0089 \| \| 0.0032 \| \| 0.0222 \| \| 0.0300 \| \| 0.0458 \| \| 0.0333 \| \| 0.0404 \| \| 0.0292 \| \| 0.0187 \| \| 0.0127 \| \| 0.0297 \| \| 0.0024 \| \| 0.0420 \| \| 0.0160 \| \| 0.0313 \| \| 0.0280 \| \| 0.0134 \| \| 0.0443 \| \| 0.0209 \| \| 0.0358 \| \| 0.0080 \| \| 0.0450 \| \| 0.0472 \| \| 0.0176 \| \| 0.0332 \| \| 0.0395 \| \| 0.0134 \| \| 0.0364 \| \| 0.0255 \| \| 0.0372 \| \| 0.0234 \| \| 0.0104 \| \| 0.0221 \| \| 0.0440 \| \| 0.0419 \| \| 0.0350 \| \| 0.0124 \| \| 0.0357 \| \| 0.0340 \| \| 0.0239 \| \| 0.0255 \| \| 0.0368 \| \| 0.0448 \| \| 0.0049 \| \| 0.0214 \| \| 0.0499 \| \| 0.0423 \| \| 0.0488 \| \| 0.0025 \| \| 0.0225 \| \| 0.0292 \| \| 0.0471 \| \| 0.0239 \| \| 0.0172 \| \| 0.0461 \| \| 0.0078 \| \| 0.0037 \| \| 0.0205 \| \| 0.0356 \| \| 0.0233 \| \| 0.0011 \| \| 0.0428 \| \| 0.0209 \| \| 0.0415 \| \| 0.0451 \| \| 0.0451 \| \| 0.0277 \| \| 0.0063 \| \| 0.0477 \| \| 0.0401 \| \| 0.0081 \| \| 0.0003 \| \| 0.0483 \| \| 0.0123 \| \| 0.0282 \| \| 0.0447 \| \| 0.0092 \| \| 0.0249 \| \| 0.0490 \| \| 0.0203 \| \| 0.0288 \| \| 0.0294 \| \| 0.0459 \| \| 0.0374 \| \| 0.0282 \| \| 0.0211 \| \| 0.0365 \| \| 0.0195 \| \| 0.0291 \| \| 0.0183 \| \| 0.0007 \| \| 0.0376 \| \| 0.0393 \| \| 0.0029 \| \| 0.0299 \| \| 0.0473 \| \| 0.0160 \| \| 0.0302 \| \| 0.0412 \| \| 0.0131 \| \| 0.0363 \| \| 0.0235 \| \| 0.0115 \| \| 0.0294 \| \| 0.0228 \| \| 0.0339 \| \| 0.0229 \| \| 0.0171 \| \| 0.0079 \| \| 0.0492 \| \| 0.0114 \| \| 0.0040 \| \| 0.0333 \| \| 0.0201 \| \| 0.0353 \| \| 0.0257 \| \| 0.0220 \| \| 0.0466 \| \| 0.0429 \| \| 0.0187 \| \| 0.0331 \| \| 0.0145 \| \| 0.0484 \| \| 0.0329 \| \| 0.0227 \| \| 0.0090 \| \| 0.0408 \| \| 0.0318 \| \| 0.0032 \| \| 0.0380 \| \| 0.0130 \| \| 0.0199 \| \| 0.0201 \| \| 0.0431 \| \| 0.0481 \| \| 0.0465 \| \| 0.0234 \| \| 0.0131 \| \| 0.0266 \| \| 0.0273 \| \| 0.0219 \| \| 0.0379 \| \| 0.0468 \| \| 0.0285 \| \| 0.0342 \| \| 0.0449 \| \| 0.0033 \| \| 0.0394 \| \| 0.0126 \| \| 0.0153 \| \| 0.0167 \| \| 0.0044 \| \| 0.0387 \| \| 0.0267 \| \| 0.0213 \| \| 0.0125 \| \| 0.0283 \| \| 0.0427 \| \| 0.0257 \| \| 0.0386 \| \| 0.0435 \| \| 0.0257 \| \| 0.0355 \| \| 0.0380 \| \| 0.0266 \| \| 0.0212 \| \| 0.0486 \| \| 0.0113 \| \| 0.0110 \| \| 0.0010 \| \| 0.0462 \| \| 0.0396 \| \| 0.0339 \| \| 0.0345 \| \| 0.0078 \| \| 0.0266 \| \| 0.0390 \| \| 0.0317 \| \| 0.0362 \| \| 0.0074 \| \| 0.0100 \| \| 0.0210 \| \| 0.0050 \| \| 0.0285 \| \| 0.0385 \| \| 0.0150 \| \| 0.0284 \| \| 0.0213 \| \| 0.0256 \| \| 0.0340 \| \| 0.0280 \| \| 0.0410 \| \| 0.0214 \| \| 0.0119 \| \| 0.0382 \| \| 0.0433 \| \| 0.0149 \| \| 0.0351 \| \| 0.0326 \| \| 0.0161 \| \| 0.0152 \| \| 0.0472 \| \| 0.0329 \| \| 0.0436 \| \| 0.0257 \| \| 0.0236 \| \| 0.0294 \| \| 0.0487 \| \| 0.0018 \| \| 0.0305 \| \| 0.0145 \| \| 0.0111 \| \| 0.0455 \| \| 0.0279 \| \| 0.0281 \| \| 0.0462 \| \| 0.0120 \| \| 0.0473 \| \| 0.0150 \| \| 0.0083 \| \| 0.0194 \| \| 0.0007 \| \| 0.0328 \| \| 0.0494 \| \| 0.0041 \| \| 0.0148 \| \| 0.0207 \| \| 0.0134 \| \| 0.0310 \| \| 0.0112 \| \| 0.0294 \| \| 0.0445 \| \| 0.0329 \| \| 0.0218 \| \| 0.0411 \| \| 0.0189 \| \| 0.0171 \| \| 0.0471 \| \| 0.0355 \| \| 0.0063 \| \| 0.0122 \| \| 0.0468 \| \| 0.0277 \| \| 0.0254 \| \| 0.0477 \| \| 0.0447 \| \| 0.0308 \| \| 0.0375 \| \| 0.0182 \| \| 0.0005 \| \| 0.0246 \| \| 0.0442 \| \| 0.0443 \| \| 0.0226 \| \| 0.0279 \| \| 0.0077 \| \| 0.0348 \| \| 0.0276 \| \| 0.0353 \| \| 0.0031 \| \| 0.0097 \| \| 0.0124 \| \| 0.0202 \| \| 0.0476 \| \| 0.0260 \| \| 0.0047 \| \| 0.0232 \| \| 0.0255 \| \| 0.0186 \| \| 0.0183 \| \| 0.0201 \| \| 0.0358 \| \| 0.0217 \| \| 0.0122 \| \| 0.0493 \| \| 0.0421 \| \| 0.0286 \| \| 0.0024 \| \| 0.0327 \| \| 0.0324 \| \| 0.0462 \| \| 0.0346 \| \| 0.0043 \| \| 0.0129 \| \| 0.0494 \| \| 0.0147 \| \| 0.0442 \| \| 0.0403 \| \| 0.0140 \| \| 0.0065 \| \| 0.0329 \| \| 0.0261 \| \| 0.0064 \| \| 0.0377 \| \| 0.0370 \| \| 0.0103 \| \| 0.0436 \| \| 0.0419 \| \| 0.0131 \| \| 0.0033 \| \| 0.0263 \| \| 0.0490 \| \| 0.0486 \| \| 0.0487 \| \| 0.0359 \| \| 0.0064 \| \| 0.0164 \| \| 0.0257 \| \| 0.0450 \| \| 0.0290 \| \| 0.0248 \| \| 0.0138 \| \| 0.0267 \| \| 0.0417 \| \| 0.0219 \| \| 0.0375 \| \| 0.0378 \| \| 0.0073 \| \| 0.0328 \| \| 0.0174 \| \| 0.0200 \| \| 0.0292 \| \| 0.0097 \| \| 0.0005 \| \| 0.0228 \| \| 0.0487 \| \| 0.0457 \| \| 0.0014 \| \| 0.0053 \| \| 0.0242 \| \| 0.0458 \| \| 0.0025 \| \| 0.0057 \| \| 0.0459 \| \| 0.0422 \| \| 0.0048 \| \| 0.0258 \| \| 0.0383 \| \| 0.0165 \| \| 0.0329 \| \| 0.0304 \| \| 0.0425 \| \| 0.0060 \| \| 0.0472 \| \| 0.0451 \| \| 0.0458 \| \| 0.0320 \| \| 0.0012 \| \| 0.0299 \| \| 0.0280 \| \| 0.0239 \| \| 0.0481 \| \| 0.0017 \| \| 0.0431 \| \| 0.0321 \| \| 0.0371 \| \| 0.0081 \| \| 0.0443 \| \| 0.0172 \| \| 0.0370 \| \| 0.0497 \| \| 0.0258 \| \| 0.0425 \| \| 0.0486 \| \| 0.0044 \| \| 0.0313 \| \| 0.0136 \| \| 0.0437 \| \| 0.0191 \| \| 0.0364 \| \| 0.0386 \| \| 0.0271 \| \| 0.0071 \| \| 0.0426 \| \| 0.0046 \| \| 0.0293 \| \| 0.0200 \| \| 0.0405 \| \| 0.0446 \| \| 0.0439 \| \| 0.0130 \| \| 0.0440 \| \| 0.0004 \| \| 0.0420 \| \| 0.0415 \| \| 0.0277 \| \| 0.0083 \| \| 0.0284 \| \| 0.0193 \| \| 0.0264 \| \| 0.0340 \| \| 0.0060 \| \| 0.0141 \| \| 0.0137 \| \| 0.0435 \| \| 0.0347 \| \| 0.0026 \| \| 0.0093 \| \| 0.0204 \| \| 0.0035 \| \| 0.0009 \| \| 0.0191 \| \| 0.0440 \| \| 0.0225 \| \| 0.0181 \| \| 0.0303 \| \| 0.0345 \| \| 0.0246 \| \| 0.0095 \| \| 0.0070 \| \| 0.0281 \| \| 0.0027 \| \| 0.0156 \| \| 0.0222 \| \| 0.0267 \| \| 0.0119 \| \| 0.0218 \| \| 0.0173 \| \| 0.0070 \| \| 0.0251 \| \| 0.0089 \| \| 0.0356 \| \| 0.0145 \| \| 0.0291 \| \| 0.0086 \| \| 0.0171 \| \| 0.0443 \| \| 0.0198 \| \| 0.0128 \| \| 0.0155 \| \| 0.0029 \| \| 0.0069 \| \| 0.0236 \| \| 0.0470 \| \| 0.0078 \| \| 0.0259 \| \| 0.0426 \| \| 0.0172 \| \| 0.0151 \| \| 0.0115 \| \| 0.0401 \| \| 0.0350 \| \| 0.0176 \| \| 0.0458 \| \| 0.0113 \| \| 0.0021 \| \| 0.0007 \| \| 0.0372 \| \| 0.0116 \| \| 0.0474 \| \| 0.0125 \| \| 0.0403 \| \| 0.0067 \| \| 0.0130 \| \| 0.0052 \| \| 0.0349 \| \| 0.0137 \| \| 0.0221 \| \| 0.0263 \| \| 0.0322 \| \| 0.0218 \| \| 0.0219 \| \| 0.0357 \| \| 0.0220 \| \| 0.0068 \| \| 0.0194 \| \| 0.0304 \| \| 0.0205 \| \| 0.0075 \| \| 0.0157 \| \| 0.0066 \| \| 0.0278 \| \| 0.0314 \| \| 0.0355 \| \| 0.0439 \| \| 0.0186 \| \| 0.0254 \| \| 0.0269 \| \| 0.0074 \| \| 0.0211 \| \| 0.0125 \| \| 0.0245 \| \| 0.0172 \| \| 0.0363 \| \| 0.0278 \| \| 0.0312 \| \| 0.0290 \| \| 0.0431 \| \| 0.0272 \| \| 0.0147 \| \| 0.0141 \| \| 0.0351 \| \| 0.0148 \| \| 0.0389 \| \| 0.0037 \| \| 0.0233 \| \| 0.0233 \| \| 0.0425 \| \| 0.0477 \| \| 0.0465 \| \| 0.0392 \| \| 0.0155 \| \| 0.0079 \| \| 0.0027 \| \| 0.0312 \| \| 0.0410 \| \| 0.0267 \| \| 0.0427 \| \| 0.0188 \| \| 0.0229 \| \| 0.0417 \| \| 0.0183 \| \| 0.0196 \| \| 0.0457 \| \| 0.0338 \| \| 0.0256 \| \| 0.0095 \| \| 0.0359 \| \| 0.0348 \| \| 0.0134 \| \| 0.0153 \| \| 0.0276 \| \| 0.0145 \| \| 0.0325 \| \| 0.0485 \| \| 0.0126 \| \| 0.0018 \| \| 0.0398 \| \| 0.0212 \| \| 0.0196 \| \| 0.0109 \| \| 0.0363 \| \| 0.0392 \| \| 0.0173 \| \| 0.0224 \| \| 0.0190 \| \| 0.0190 \| \| 0.0406 \| \| 0.0490 \| \| 0.0368 \| \| 0.0044 \| \| 0.0112 \| \| 0.0316 \| \| 0.0414 \| \| 0.0241 \| \| 0.0210 \| \| 0.0052 \| \| 0.0135 \| \| 0.0045 \| \| 0.0218 \| \| 0.0217 \| \| 0.0387 \| \| 0.0143 \| \| 0.0429 \| \| 0.0417 \| \| 0.0430 \| \| 0.0020 \| \| 0.0300 \| \| 0.0228 \| \| 0.0104 \| \| 0.0199 \| \| 0.0496 \| \| 0.0451 \| \| 0.0057 \| \| 0.0331 \| \| 0.0372 \| \| 0.0384 \| \| 0.0110 \| \| 0.0208 \| \| 0.0066 \| \| 0.0049 \| \| 0.0129 \| \| 0.0167 \| \| 0.0159 \| \| 0.0309 \| \| 0.0342 \| \| 0.0208 \| \| 0.0479 \| \| 0.0452 \| \| 0.0247 \| \| 0.0277 \| \| 0.0276 \| \| 0.0139 \| \| 0.0075 \| \| 0.0310 \| \| 0.0446 \| \| 0.0014 \| \| 0.0355 \| \| 0.0467 \| \| 0.0364 \| \| 0.0303 \| \| 0.0465 \| \| 0.0305 \| \| 0.0064 \| \| 0.0183 \| \| 0.0295 \| \| 0.0234 \| \| 0.0245 \| \| 0.0064 \| \| 0.0416 \| \| 0.0122 \| \| 0.0046 \| \| 0.0429 \| \| 0.0244 \| \| 0.0111 \| \| 0.0081 \| \| 0.0209 \| \| 0.0315 \| \| 0.0279 \| \| 0.0075 \| \| 0.0485 \| \| 0.0108 \| \| 0.0334 \| \| 0.0263 \| \| 0.0255 \| \| 0.0482 \| \| 0.0164 \| \| 0.0099 \| \| 0.0173 \| \| 0.0216 \| \| 0.0474 \| \| 0.0250 \| \| 0.0026 \| \| 0.0206 \| \| 0.0173 \| \| 0.0425 \| \| 0.0400 \| \| 0.0355 \| \| 0.0314 \| \| 0.0402 \| \| 0.0034 \| \| 0.0034 \| \| 0.0026 \| \| 0.0110 \| \| 0.0355 \| \| 0.0339 \| \| 0.0483 \| \| 0.0155 \| \| 0.0141 \| \| 0.0174 \| \| 0.0135 \| \| 0.0220 \| \| 0.0276 \| \| 0.0118 \| \| 0.0009 \| \| 0.0402 \| \| 0.0130 \| \| 0.0311 \| \| 0.0088 \| \| 0.0474 \| \| 0.0393 \| \| 0.0222 \| \| 0.0256 \| \| 0.0454 \| \| 0.0038 \| \| 0.0387 \| \| 0.0472 \| \| 0.0307 \| \| 0.0419 \| \| 0.0130 \| \| 0.0266 \| \| 0.0346 \| \| 0.0119 \| \| 0.0134 \| \| 0.0184 \| \| 0.0070 \| \| 0.0220 \| \| 0.0096 \| \| 0.0145 \| \| 0.0499 \| \| 0.0402 \| \| 0.0123 \| \| 0.0080 \| \| 0.0200 \| \| 0.0331 \| \| 0.0421 \| \| 0.0147 \| \| 0.0413 \| \| 0.0453 \| \| 0.0403 \| \| 0.0419 \| \| 0.0350 \| \| 0.0262 \| \| 0.0476 \| \| 0.0087 \| \| 0.0363 \| \| 0.0370 \| \| 0.0388 \| \| 0.0353 \| \| 0.0166 \| \| 0.0061 \| \| 0.0063 \| \| 0.0244 \| \| 0.0151 \| \| 0.0413 \| \| 0.0083 \| \| 0.0106 \| \| 0.0010 \| \| 0.0385 \| \| 0.0434 \| \| 0.0023 \| \| 0.0021 \| \| 0.0452 \| \| 0.0046 \| \| 0.0026 \| \| 0.0311 \| \| 0.0059 \| \| 0.0291 \| \| 0.0189 \| \| 0.0273 \| \| 0.0368 \| \| 0.0263 \| \| 0.0171 \| \| 0.0105 \| \| 0.0159 \| \| 0.0223 \| \| 0.0146 \| \| 0.0453 \| \| 0.0011 \| \| 0.0132 \| \| 0.0471 \| \| 0.0469 \| \| 0.0346 \| \| 0.0038 \| \| 0.0106 \| \| 0.0045 \| \| 0.0193 \| \| 0.0366 \| \| 0.0002 \| \| 0.0112 \| \| 0.0265 \| \| 0.0059 \| \| 0.0011 \| \| 0.0496 \| \| 0.0355 \| \| 0.0293 \| \| 0.0039 \| \| 0.0368 \| \| 0.0154 \| \| 0.0091 \| \| 0.0444 \| \| 0.0012 \| \| 0.0359 \| \| 0.0179 \| \| 0.0233 \| \| 0.0139 \| \| 0.0347 \| \| 0.0383 \| \| 0.0004 \| \| 0.0097 \| \| 0.0269 \| \| 0.0189 \| \| 0.0282 \| \| 0.0157 \| \| 0.0135 \| \| 0.0436 \| \| 0.0057 \| \| 0.0030 \| \| 0.0387 \| \| 0.0077 \| \| 0.0003 \| \| 0.0404 \| \| 0.0060 \| \| 0.0202 \| \| 0.0293 \| \| 0.0085 \| \| 0.0409 \| \| 0.0049 \| \| 0.0287 \| \| 0.0125 \| \| 0.0215 \| \| 0.0428 \| \| 0.0057 \| \| 0.0218 \| \| 0.0045 \| \| 0.0016 \| \| 0.0178 \| \| 0.0004 \| \| 0.0235 \| \| 0.0332 \| \| 0.0104 \| \| 0.0071 \| \| 0.0122 \| \| 0.0298 \| \| 0.0399 \| \| 0.0430 \| \| 0.0048 \| \| 0.0461 \| \| 0.0222 \| \| 0.0123 \| \| 0.0077 \| \| 0.0020 \| \| 0.0041 \| \| 0.0456 \| \| 0.0191 \| \| 0.0070 \| \| 0.0476 \| \| 0.0373 \| \| 0.0211 \| \| 0.0301 \| \| 0.0016 \| \| 0.0335 \| \| 0.0305 \| \| 0.0467 \| \| 0.0256 \| \| 0.0387 \| \| 0.0222 \| \| 0.0269 \| \| 0.0165 \| \| 0.0408 \| \| 0.0224 \| \| 0.0157 \| \| 0.0054 \| \| 0.0307 \| \| 0.0251 \| \| 0.0424 \| \| 0.0192 \| \| 0.0004 \| \| 0.0280 \| \| 0.0053 \| \| 0.0248 \| \| 0.0044 \| \| 0.0101 \| \| 0.0265 \| \| 0.0143 \| \| 0.0087 \| \| 0.0391 \| \| 0.0111 \| \| 0.0154 \| \| 0.0439 \| \| 0.0314 \| \| 0.0284 \| \| 0.0136 \| \| 0.0321 \| \| 0.0429 \| \| 0.0487 \| \| 0.0360 \| \| 0.0040 \| \| 0.0251 \| \| 0.0172 \| \| 0.0429 \| \| 0.0232 \| \| 0.0489 \| \| 0.0082 \| \| 0.0117 \| \| 0.0254 \| \| 0.0190 \| \| 0.0131 \| \| 0.0478 \| \| 0.0191 \| \| 0.0349 \| \| 0.0258 \| \| 0.0018 \| \| 0.0139 \| \| 0.0303 \| \| 0.0132 \| \| 0.0348 \| \| 0.0218 \| \| 0.0231 \| \| 0.0416 \| \| 0.0085 \| \| 0.0489 \| \| 0.0126 \| \| 0.0328 \| \| 0.0441 \| \| 0.0248 \| \| 0.0460 \| \| 0.0222 \| \| 0.0219 \| \| 0.0082 \| \| 0.0017 \| \| 0.0107 \| \| 0.0186 \| \| 0.0121 \| \| 0.0476 \| \| 0.0030 \| \| 0.0353 \| \| 0.0350 \| \| 0.0075 \| \| 0.0481 \| \| 0.0220 \| \| 0.0347 \| \| 0.0436 \| \| 0.0111 \| \| 0.0245 \| \| 0.0470 \| \| 0.0143 \| \| 0.0079 \| \| 0.0366 \| \| 0.0347 \| \| 0.0171 \| \| 0.0243 \| \| 0.0336 \| \| 0.0014 \| \| 0.0209 \| \| 0.0214 \| \| 0.0291 \| \| 0.0442 \| \| 0.0305 \| \| 0.0116 \| \| 0.0274 \| \| 0.0065 \| \| 0.0134 \| \| 0.0056 \| \| 0.0260 \| \| 0.0269 \| \| 0.0454 \| \| 0.0297 \| \| 0.0079 \| \| 0.0264 \| \| 0.0181 \| \| 0.0219 \| \| 0.0059 \| \| 0.0235 \| \| 0.0311 \| \| 0.0291 \| \| 0.0026 \| \| 0.0283 \| \| 0.0347 \| \| 0.0181 \| \| 0.0113 \| \| 0.0108 \| \| 0.0315 \| \| 0.0468 \| \| 0.0431 \| \| 0.0155 \| \| 0.0072 \| \| 0.0023 \| \| 0.0133 \| \| 0.0279 \| \| 0.0494 \| \| 0.0177 \| \| 0.0381 \| \| 0.0065 \| \| 0.0137 \| \| 0.0031 \| \| 0.0477 \| \| 0.0293 \| \| 0.0302 \| \| 0.0078 \| \| 0.0296 \| \| 0.0318 \| \| 0.0151 \| \| 0.0116 \| \| 0.0314 \| \| 0.0265 \| \| 0.0455 \| \| 0.0149 \| \| 0.0198 \| \| 0.0306 \| \| 0.0244 \| \| 0.0153 \| \| 0.0234 \| \| 0.0006 \| \| 0.0052 \| \| 0.0217 \| \| 0.0062 \| \| 0.0028 \| \| 0.0425 \| \| 0.0181 \| \| 0.0150 \| \| 0.0223 \| \| 0.0126 \| \| 0.0310 \| \| 0.0066 \| \| 0.0032 \| \| 0.0398 \| \| 0.0292 \| \| 0.0217 \| \| 0.0206 \| \| 0.0239 \| \| 0.0399 \| \| 0.0202 \| \| 0.0132 \| \| 0.0383 \| \| 0.0197 \| \| 0.0045 \| \| 0.0495 \| \| 0.0212 \| \| 0.0160 \| \| 0.0296 \| \| 0.0046 \| \| 0.0212 \| \| 0.0025 \| \| 0.0143 \| \| 0.0139 \| \| 0.0041 \| \| 0.0312 \| \| 0.0070 \| \| 0.0171 \| \| 0.0166 \| \| 0.0243 \| \| 0.0062 \| \| 0.0216 \| \| 0.0414 \| \| 0.0248 \| \| 0.0174 \| \| 0.0421 \| \| 0.0106 \| \| 0.0067 \| \| 0.0088 \| \| 0.0079 \| \| 0.0008 \| \| 0.0223 \| \| 0.0487 \| \| 0.0044 \| \| 0.0459 \| \| 0.0216 \| \| 0.0131 \| \| 0.0170 \| \| 0.0061 \| \| 0.0004 \| \| 0.0357 \| \| 0.0256 \| \| 0.0120 \| \| 0.0028 \| \| 0.0485 \| \| 0.0282 \| \| 0.0444 \| \| 0.0043 \| \| 0.0330 \| \| 0.0429 \| \| 0.0299 \| \| 0.0225 \| \| 0.0077 \| \| 0.0103 \| \| 0.0360 \| \| 0.0424 \| \| 0.0301 \| \| 0.0190 \| \| 0.0113 \| \| 0.0108 \| \| 0.0419 \| \| 0.0147 \| \| 0.0144 \| \| 0.0393 \| \| 0.0296 \| \| 0.0040 \| \| 0.0382 \| \| 0.0155 \| \| 0.0065 \| \| 0.0164 \| \| 0.0016 \| \| 0.0189 \| \| 0.0479 \| \| 0.0268 \| \| 0.0161 \| \| 0.0188 \| \| 0.0119 \| \| 0.0114 \| \| 0.0028 \| \| 0.0133 \| \| 0.0060 \| \| 0.0404 \| \| 0.0271 \| \| 0.0476 \| \| 0.0407 \| \| 0.0425 \| \| 0.0028 \| \| 0.0337 \| \| 0.0102 \| \| 0.0159 \| \| 0.0044 \| \| 0.0361 \| \| 0.0215 \| \| 0.0113 \| \| 0.0275 \| \| 0.0461 \| \| 0.0197 \| \| 0.0449 \| \| 0.0102 \| \| 0.0147 \| \| 0.0262 \| \| 0.0197 \| \| 0.0404 \| \| 0.0046 \| \| 0.0379 \| \| 0.0309 \| \| 0.0027 \| \| 0.0134 \| \| 0.0133 \| \| 0.0260 \| \| 0.0278 \| \| 0.0318 \| \| 0.0365 \| \| 0.0016 \| \| 0.0311 \| \| 0.0101 \| \| 0.0272 \| \| 0.0096 \| \| 0.0095 \| \| 0.0495 \| \| 0.0188 \| \| 0.0062 \| \| 0.0448 \| \| 0.0129 \| \| 0.0126 \| \| 0.0055 \| \| 0.0049 \| \| 0.0098 \| \| 0.0057 \| \| 0.0352 \| \| 0.0258 \| \| 0.0042 \| \| 0.0473 \| \| 0.0220 \| \| 0.0068 \| \| 0.0002 \| \| 0.0288 \| \| 0.0064 \| \| 0.0014 \| \| 0.0217 \| \| 0.0091 \| \| 0.0067 \| \| 0.0289 \| \| 0.0054 \| \| 0.0110 \| \| 0.0342 \| \| 0.0124 \| \| 0.0099 \| \| 0.0442 \| \| 0.0198 \| \| 0.0090 \| \| 0.0061 \| \| 0.0072 \| \| 0.0021 \| \| 0.0497 \| \| 0.0026 \| \| 0.0090 \| \| 0.0089 \| \| 0.0221 \| \| 0.0488 \| \| 0.0372 \| \| 0.0076 \| \| 0.0053 \| \| 0.0017 \| \| 0.0236 \| \| 0.0100 \| \| 0.0215 \| \| 0.0148 \| \| 0.0004 \| \| 0.0325 \| \| 0.0005 \| \| 0.0144 \| \| 0.0416 \| \| 0.0092 \| \| 0.0169 \| \| 0.0104 \| \| 0.0261 \| \| 0.0144 \| \| 0.0062 \| \| 0.0345 \| \| 0.0031 \| \| 0.0063 \| \| 0.0035 \| \| 0.0008 \| \| 0.0306 \| \| 0.0319 \| \| 0.0007 \| \| 0.0025 \| \| 0.0195 \| \| 0.0012 \| \| 0.0000 \| \| 0.0032 \| \| 0.0027 \| \| 0.0332 \| \| 0.0144 \| \| 0.0169 \| \| 0.0186 \| \| 0.0045 \| \| 0.0485 \| \| 0.0265 \| \| 0.0268 \| \| 0.0078 \| \| 0.0257 \| \| 0.0163 \| \| 0.0189 \| \| 0.0384 \| \| 0.0435 \| \| 0.0090 \| \| 0.0291 \| \| 0.0269 \| \| 0.0318 \| \| 0.0053 \| \| 0.0355 \| \| 0.0060 \| \| 0.0446 \| \| 0.0331 \| \| 0.0053 \| \| 0.0064 \| \| 0.0097 \| \| 0.0073 \| \| 0.0177 \| \| 0.0149 \| \| 0.0191 \| \| 0.0057 \| \| 0.0094 \| \| 0.0169 \| \| 0.0132 \| \| 0.0155 \| \| 0.0277 \| \| 0.0404 \| \| 0.0084 \| \| 0.0218 \| \| 0.0382 \| \| 0.0030 \| \| 0.0019 \| \| 0.0142 \| \| 0.0060 \| \| 0.0191 \| \| 0.0222 \| \| 0.0248 \| \| 0.0159 \| \| 0.0065 \| \| 0.0154 \| \| 0.0104 \| \| 0.0227 \| \| 0.0042 \| \| 0.0043 \| \| 0.0123 \| \| 0.0069 \| \| 0.0032 \| \| 0.0126 \| \| 0.0104 \| \| 0.0385 \| \| 0.0250 \| \| 0.0253 \| \| 0.0019 \| \| 0.0069 \| \| 0.0091 \| \| 0.0095 \| \| 0.0116 \| \| 0.0240 \| \| 0.0111 \| \| 0.0096 \| \| 0.0392 \| \| 0.0220 \| \| 0.0108 \| \| 0.0240 \| \| 0.0460 \| \| 0.0135 \| \| 0.0232 \| \| 0.0080 \| \| 0.0152 \| \| 0.0221 \| \| 0.0111 \| \| 0.0271 \| \| 0.0033 \| \| 0.0332 \| \| 0.0291 \| \| 0.0026 \| \| 0.0008 \| \| 0.0175 \| \| 0.0008 \| \| 0.0078 \| \| 0.0155 \| \| 0.0018 \| \| 0.0005 \| \| 0.0213 \| \| 0.0096 \| \| 0.0063 \| \| 0.0108 \| \| 0.0370 \| \| 0.0085 \| \| 0.0223 \| \| 0.0487 \| \| 0.0424 \| \| 0.0485 \| \| 0.0471 \| \| 0.0016 \| \| 0.0190 \| \| 0.0385 \| \| 0.0323 \| \| 0.0198 \| \| 0.0266 \| \| 0.0380 \| \| 0.0222 \| \| 0.0026 \| \| 0.0024 \| \| 0.0000 \| \| 0.0400 \| \| 0.0085 \| \| 0.0017 \| \| 0.0318 \| \| 0.0211 \| \| 0.0037 \| \| 0.0392 \| \| 0.0331 \| \| 0.0001 \| \| 0.0329 \| \| 0.0359 \| \| 0.0476 \| \| 0.0114 \| \| 0.0220 \| \| 0.0037 \| \| 0.0036 \| \| 0.0186 \| \| 0.0482 \| \| 0.0416 \| \| 0.0475 \| \| 0.0344 \| \| 0.0314 \| \| 0.0010 \| \| 0.0073 \| \| 0.0493 \| \| 0.0264 \| \| 0.0008 \| \| 0.0466 \| \| 0.0185 \| \| 0.0225 \| \| 0.0186 \| \| 0.0008 \| \| 0.0163 \| \| 0.0331 \| \| 0.0393 \| \| 0.0097 \| \| 0.0289 \| \| 0.0335 \| \| 0.0126 \| \| 0.0033 \| \| 0.0451 \| \| 0.0036 \| \| 0.0004 \| \| 0.0001 \| \| 0.0294 \| \| 0.0296 \| \| 0.0326 \| \| 0.0093 \| \| 0.0014 \| \| 0.0256 \| \| 0.0105 \| \| 0.0004 \| \| 0.0192 \| \| 0.0457 \| \| 0.0336 \| \| 0.0344 \| \| 0.0056 \| \| 0.0121 \| \| 0.0006 \| \| 0.0276 \| \| 0.0080 \| \| 0.0441 \| \| 0.0007 \| \| 0.0046 \| \| 0.0026 \| \| 0.0189 \| \| 0.0097 \| \| 0.0162 \| \| 0.0048 \| \| 0.0020 \| \| 0.0110 \| \| 0.0162 \| \| 0.0014 \| \| 0.0256 \| \| 0.0090 \| \| 0.0190 \| \| 0.0119 \| \| 0.0059 \| \| 0.0256 \| \| 0.0121 \| \| 0.0124 \| \| 0.0114 \| \| 0.0468 \| \| 0.0483 \| \| 0.0002 \| \| 0.0151 \| \| 0.0161 \| \| 0.0328 \| \| 0.0021 \| \| 0.0000 \| \| 0.0073 \| \| 0.0351 \| \| 0.0130 \| \| 0.0018 \| \| 0.0051 \| \| 0.0052 \| \| 0.0149 \| \| 0.0054 \| \| 0.0090 \| \| 0.0006 \| \| 0.0086 \| \| 0.0065 \| \| 0.0035 \| \| 0.0008 \| \| 0.0051 \| \| 0.0034 \| \| 0.0023 \| \| 0.0133 \| \| 0.0235 \| \| 0.0058 \| \| 0.0001 \| \| 0.0206 \| \| 0.0092 \| \| 0.0081 \| \| 0.0053 \| \| 0.0043 \| \| 0.0025 \| \| 0.0417 \| \| 0.0021 \| \| 0.0056 \| \| 0.0050 \| \| 0.0449 \| \| 0.0220 \| \| 0.0228 \| \| 0.0017 \| \| 0.0005 \| \| 0.0294 \| \| 0.0020 \| \| 0.0185 \| \| 0.0011 \| \| 0.0445 \| \| 0.0019 \| \| 0.0144 \| \| 0.0061 \| \| 0.0009 \| \| 0.0060 \| \| 0.0008 \| \| 0.0098 \| \| 0.0012 \| \| 0.0108 \| \| 0.0021 \| \| 0.0171 \| \| 0.0003 \| \| 0.0169 \| \| 0.0008 \| \| 0.0332 \| \| 0.0396 \| \| 0.0007 \| \| 0.0481 \| \| 0.0130 \| \| 0.0068 \| \| 0.0037 \| \| 0.0003 \| \| 0.0030 \| \| 0.0154 \| \| 0.0419 \| \| 0.0331 \| \| 0.0084 \| \| 0.0022 \| \| 0.0104 \| \| 0.0291 \| \| 0.0332 \| \| 0.0012 \| \| 0.0389 \| \| 0.0007 \| \| 0.0008 \| |
