# Supplementary material for: An Exploration of Heat Tolerance in Mice Utilizing mRNA and microRNA Expression Analysis
Source: PLoS One. 2013 Aug 15;8(8):e72258. doi: 10.1371/journal.pone.0072258 (PMC3744453; doi:10.1371/journal.pone.0072258)
Supplement: Table S2 — List of 61 miRNA seed sequences identified using microarray to be differentially expressed in TOL mice compared to INT mice with respective fold change and p value of significance. (DOCX) [file pone.0072258.s002.docx]

**Table S2. List of 61 miRNA seed sequences identified using microarray to be differentially expressed in TOL mice compared to INT mice with respective fold change and p value of significance.**

| **miRNA seed sequences** | **Microarray fold change** | **P value** | **miRNA seed sequences** | **Microarray fold change** | **P value** |
| --- | --- | --- | --- | --- | --- |
| miR-1945  miR-211  miR-669f  miR-879-star  miR-671-5p  miR-708-star  miR-466k  miR-344  miR-136  miR-293  miR-874  miR-30c-2  miR-363  miR-2136  miR-494  miR-351  miR-1964  miR-1954  miR-369-3p  miR-669m-2  miR-376b-star  miR-376a-star  miR-29b-1  miR-669l  miR-377  miR-34a-5p  miR-375  miR-1939  miR-467d-star  miR-153  miR-698 | 1.2  1.1  -1.2  -1.2  -1.6  1.2  1.1  1.2  1.1  -1.1  -1.1  1.1  1.2  1.2  1.2  -1.5  1.2  -1.1  1.1  -1.1  1.1  1.1  1.1  1.1  1.1  -1.2  1.1  1.1  -1.4  1.2  -1.1 | 0.0024  0.0028  0.0040  0.0049  0.0057  0.0061  0.0087  0.0093  0.0109  0.0116  0.0124  0.0127  0.0127  0.0136  0.0143  0.0169  0.0187  0.0193  0.0195  0.0197  0.0199  0.0218  0.0221  0.0230  0.0231  0.0236  0.0255  0.0277  0.0288  0.0305  0.0322 | mIR-7f-2  miR-1190  miR-9-3  miR-23a  miR-702  miR-1950  miR-302a  miR-467a  miR-1937c  miR-1962  miR-30e  miR-138  miR-673-3p  miR-23b  miR-151  miR-1944  miR-669o  miR-329  miR-10a-star  miR-300  miR-692  miR-27a  miR-30b  miR-483  miR-199a-3p  miR-let-7a-2  miR-467a-1-star  miR-466b-2  miR-331-5p  miR-467f | -1.1  1.2  1.1  -1.1  -1.2  -1.1  -1.1  -1.1  -1.1  1.1  1.1  1.2  1.3  -1.1  -1.1  1.3  -1.3  -1.1  1.2  -1.1  1.1  1.1  1.1  -1.2  1.1  1.1  -1.2  1.1  -1.1  -1.3 | 0.0332  0.0334  0.0344  0.0349  0.0351  0.0357  0.0363  0.0364  0.0365  0.0366  0.0373  0.0385  0.0395  0.0404  0.0412  0.0423  0.0423  0.0424  0.0433  0.0437  0.0440  0.0451  0.0467  0.0472  0.0472  0.0477  0.0480  0.0480  0.0490  0.0500 |
